# An intestinal T_H_17 cell-derived subset can initiate cancer

**DOI:** 10.1038/s41590-024-01909-7

**Published:** 2024-07-26

**Authors:** Olivier Fesneau, Valentin Thevin, Valérie Pinet, Chloe Goldsmith, Baptiste Vieille, Saïdi M’Homa Soudja, Rossano Lattanzio, Michael Hahne, Valérie Dardalhon, Hector Hernandez-Vargas, Nicolas Benech, Julien C. Marie

**Affiliations:** 1 Cancer Research Center of Lyon (CRCL) INSERM U 1052, CNRS UMR 5286, Centre Léon Bérard, Claude Bernard Lyon 1 University, Lyon, France; 2 Institut de Génétique Moléculaire de Montpellier (IGMM), Université de Montpellier, CNRS, Montpellier, France; 3 Department of Innovative Technologies in Medicine & Dentistry, Center for Advanced Studies and Technology (CAST), G. d’Annunzio University of Chieti–Pescara, Chieti, Italy; 4 Hospices Civils de Lyon, Service d’Hépato-Gastroentérologie, Croix Rousse Hospital, Lyon, France; 5 Equipe Labellisée Ligue Nationale Contre le Cancer, Lyon, France

**Keywords:** Chronic inflammation, Tumour immunology, T-helper 17 cells

## Abstract

Approximately 25% of cancers are preceded by chronic inflammation that occurs at the site of tumor development. However, whether this multifactorial oncogenic process, which commonly occurs in the intestines, can be initiated by a specific immune cell population is unclear. Here, we show that an intestinal T cell subset, derived from interleukin-17 (IL-17)-producing helper T (T_H_17) cells, induces the spontaneous transformation of the intestinal epithelium. This subset produces inflammatory cytokines, and its tumorigenic potential is not dependent on IL-17 production but on the transcription factors KLF6 and T-BET and interferon-γ. The development of this cell type is inhibited by transforming growth factor-β1 (TGFβ1) produced by intestinal epithelial cells. TGFβ signaling acts on the pretumorigenic T_H_17 cell subset, preventing its progression to the tumorigenic stage by inhibiting KLF6-dependent T-BET expression. This study therefore identifies an intestinal T cell subset initiating cancer.

## Abstract (web summary)

Here, the authors show that there is a pretumorigenic T_H_17 subset in the intestines that can convert to being tumorigenic under the control of KLF6 and that this process can be prevented by TGFβ1 production from intestinal epithelial cells.

## Main

Chronic inflammation enables cancer through DNA damage, mutations and the modulation of oncogenes and tumor suppressor gene expression^1^. This chronic inflammation-associated cancer (CIAC) often occurs in the intestinal epithelium at the site of subsequent cancer development. This local inflammation is established long before any sign of transformation of the intestinal epithelial cells (IECs) is detected, and it likely involves several factors and cells, including immune cells^1,2^. However, whether IEC transformation in response to CIAC can be initiated by a specific immune cell population is unknown. Identifying such a population and the factors controlling its development is essential not only for our understanding of the early steps of CIAC but also for the development of prophylactic therapies against cancer.

In the mammalian intestine, interleukin-17 (IL-17)-producing CD4^+^ helper αβT (T_H_17) cells constitute the largest population of effector T cells of the small intestine lamina propria (SILP)^3^. Their optimal differentiation from naive cells occurs mostly in the mesenteric lymph nodes (mLNs)^4^ and requires the presence of at least two cytokines: IL-6 and transforming growth factor-β1 (TGFβ1)^3^. IL-6 activates STAT3 signaling and induces the expression of two transcriptional factors essential for the T_H_17 cell differentiation program, RORα and RORγt, and inhibits that of FOXP3 crucial for regulatory T (T_reg_) cell differentiation^5,6^. TGFβ exists as three forms, TGFβ1, TGFβ2 and TGFβ3, with TGFβ1 prevalent in the intestine and in the mLNs^7^. All three forms bind and activate TGFβ receptor 2 (TGFβR2), which in turn phosphorylates the kinase domain of TGFβR1. TGFβR1 can then trigger a signaling cascade through the phosphorylation of SMAD2 and SMAD3 proteins, which can bind to SMAD4 and/or TRIM33 and regulate the expression of several target genes controlling naive/T_H_17 cell differentiation^8,9^. Of note, TGFβRs can also signal independently of SMAD4 and TRIM33 through a noncanonical branch involving ERK/MAP kinase pathways^10^.

Following differentiation from naive T cells in the mLN, T_H_17 cells migrate to the SILP, to some extent to the colon and to Peyer’s patches (PP)^4^. Hence, T_H_17 cells are mainly localized in the intestine, where they contribute to intestinal homeostasis, tissue repair and bacterial protection. T_H_17 cells are also linked to several inflammatory pathologies inside and outside the intestine, and IL-17 can have either positive or negative effects on the progression of already established tumors depending on the type of cancer^11^. This ability of T_H_17 cells to exert both beneficial and pathogenic functions under different conditions and tissue environments suggests that this effector T cell type could be very heterogenous^12^.

Here, we identify a T_H_17 cell subpopulation that can initiate intestinal CIAC, leading to adenocarcinoma from a nonpremutated tissue. This tumorigenic subset develops from a pretumorigenic T_H_17 cell population present at the steady state in the intestine. The switch from pretumorigenic to a tumorigenic stage is promoted by the transcription factor (TF) KLF6 and is impaired by the effects of IEC-produced TGFβ1 on already differentiated T_H_17 cells. Hence, our work describes a cellular mechanism that can protect the intestine from cancer initiation based on an interplay between IECs and specific intestinal T_H_17 cells.

## Results

### TGFβ prevents T_H_17 cells from initiating intestinal cancer

**Fig. 1:**
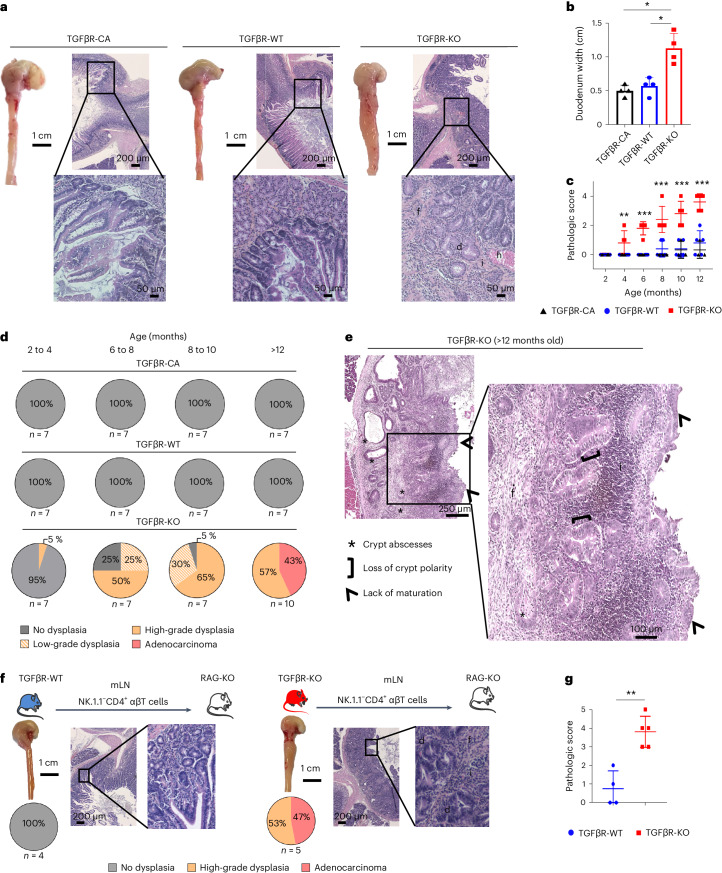
TGFβ signaling in differentiated T_H_17 cells prevents spontaneous cancer development. Spontaneous cancer development was evaluated over time in the duodenum of TGFβR-CA, TGFβR-KO and TGFβR-WT mice. **a**, Representative gross lesions and hematoxylin and eosin (H&E) histology of the duodenum of 10-month-old mice; d, dysplasia; f, fibrosis, h, hemorrhagia, i, immune cell infiltrates. **b**, The width of the duodenum of 10-month-old mice was measured (mean ± s.d.). The experiment was repeated four times (*P* = 0.0286). **c**, Graph illustrating the pathologic score of the duodenum across the life of the animal (mean ± s.d.). The experiment was repeated four times. **d**, Percentage of animals with different grades of dysplasia and cancer in the duodenum between 2 and 12 months of age. **e**, Representative H&E histology of adenocarcinoma observed at the duodenal bulb of TGFβR-KO animals after 12 months of age. **f**,**g**, NK1.1^–^CD4^+^TCRβ^+^ cells were purified from the mLNs of either TGFβR-KO or TGFβR-WT mice and injected into RAG-KO mice. Eight months later, duodenums of recipient animals were collected and analyzed. The experiment was repeated twice with three and four transfers of TGFβR-WT cells and TGFβR-KO cells, respectively, for each experiment. **f**, Representative gross lesions, H&E histology staining and percentage of recipient animals with duodenal adenocarcinoma. **g**, Pathologic scores (mean ± s.d.). The experiment was repeated three times; *P < 0.05; **P < 0.01; ***P < 0.0001. Data were analyzed by two-tailed Student’s *t-*test (**b** and **c**) or two-tailed Mann–Whitney test (**g**). Source data

**Extended Data Fig. 1:**
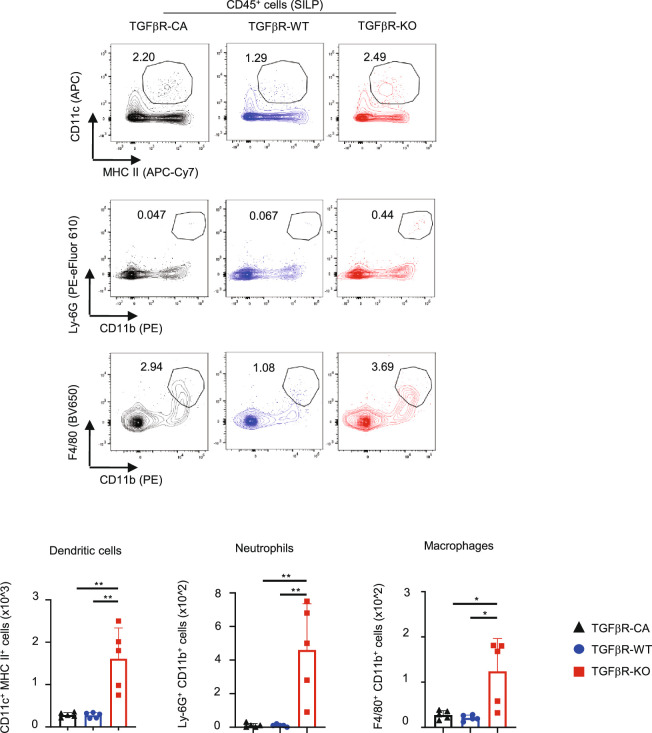
Analysis of the small intestine infiltrating innate immune cells. CD45^Pos^ cells isolated from the SLIP of the duodenum of 6 month-old TGFβR-KO and TGFβR-WT and TGFβR-CA mice were analyzed by flow cytometry. Counter plots represent the staining strategies and histogram graphs illustrate the numbers of cells per gramme of fresh tissues (mean + SD). Data are representative of 5 animals per groups in 3 independent experiments. Statistical analysis were performed using a two-tailed Student’s t test.*P<0.05; **P<0.01; ***P<0.001. Source data

Given that once differentiated, T_H_17 cells sustain their expression of TGFβRs^13^, we first analyzed whether intestinal T_H_17 cells still respond to TGFβ1. We observed that they kept responding to TGFβ1, as monitored by the phosphorylation of SMAD2/SMAD3 (Supplementary Fig. 1), suggesting that, in addition to its role in naive/T_H_17 cell differentiation, TGFβ signaling could also play a role in T_H_17 cells after differentiation. We thus aimed to investigate this role. To selectively distinguish the contribution of TGFβ signaling in T_H_17 cell differentiation versus in already differentiated T_H_17 cells, we crossed *Il17a-cre* mice^14^ with mice bearing either floxed alleles of *Tgfbr2* (TGFβR-KO)^15^ or a constitutively active (CA) form of *Tgfbr1* (TGFβR-CA) after a *stop-lox* cassette^16^. Strikingly, by 5 months of age, the gross analysis of the intestine of TGFβR-KO mice revealed an impressive enlargement and erythematous appearance of both the pyloric antrum and the proximal part of the duodenum (Fig. 1a,b). Histology of the duodenum revealed blunting of the villi to complete loss of villous structures in TGFβR-KO mice and progressive infiltration from 4 months of age of mono- and polymorphonuclear cells, including macrophages, dendritic cells and neutrophils, in the mucosa, submucosa and transmural tissue in association with fibrosis (Fig. 1a,c and Extended Data Fig. 1). This chronic inflammation was associated with signs of progressive malignancy near the pyloric region, at the bulb, with adenomatous changes of IECs characterized by nuclear enlargement and stratification, loss of cell polarity and loss of mucinous differentiation (Fig. 1d). Remarkably, by 10 months of age, nearly all TGFβR-KO mice (95%) had developed at least low-grade dysplasia, and 65% had developed high-grade dysplasia. After 1 year, 45–50% of the animals exhibited advanced adenocarcinoma of the duodenum (Fig. 1d,e). Of note, the transformation was at the bulb, a region where the vast majority of small intestine cancers are observed in humans^17^. Interestingly, histology of the colon revealed that the colonic lamina propria (CLP) of TGFβR-KO mice was also infiltrated by mono- and polymorphonuclear cells but without any sign of IEC transformation (Supplementary Fig. 2a,b). Importantly, analysis of the composition of the bacterial microbiota did not show any difference between TGFβR-KO mice and wild-type (WT) TGFβR (TGFβR-WT) mice in both α- and β-diversity, excluding a specific dysbiosis in the pathology (Supplementary Fig. 3). Of note this CIAC was still observed when T_H_17 cells were deficient for SMAD4, TRIM33 or both (Supplementary Fig. 4). Thus, in contrast to the role depicted for SMAD4 and TRIM33 in controlling the differentiation of naive cells to T_H_17 cells^8,9^, a contribution of these two TGFβ signaling actors in differentiated T_H_17 cells to prevent CIAC was unlikely.

**Extended Data Fig. 2:**
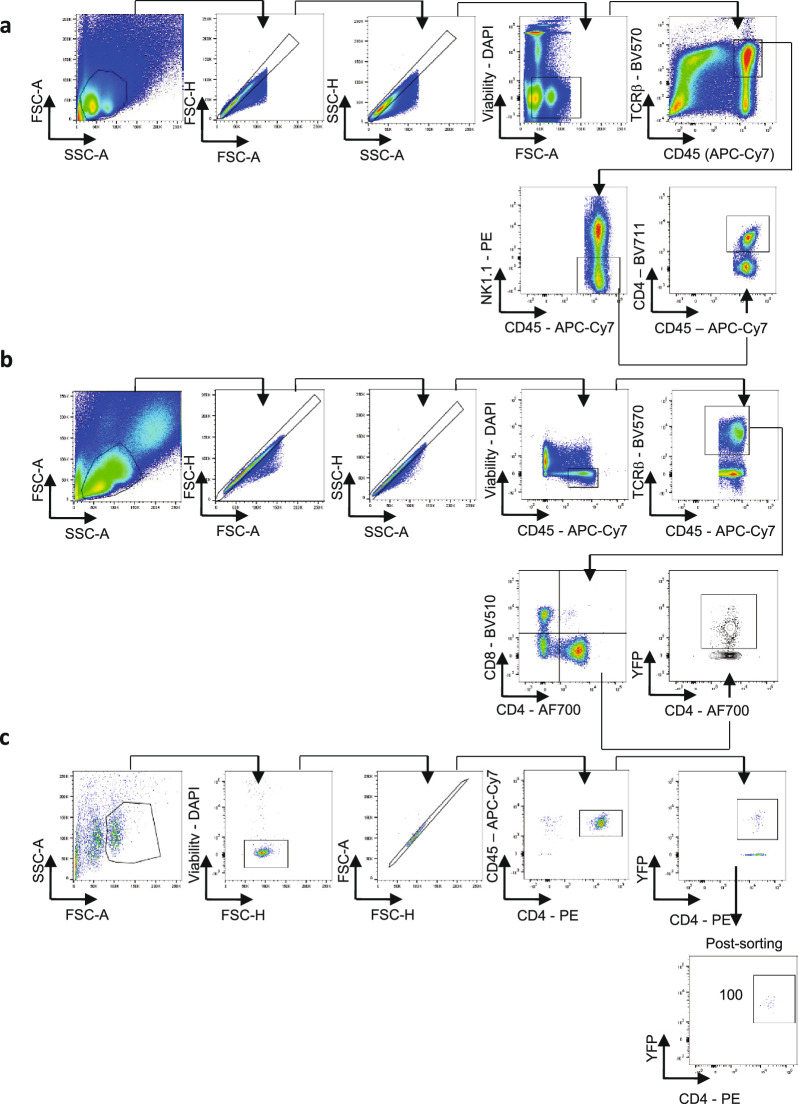
Gating strategy used for YFP^pos^ cells analysis and cell-sorting by flow cytometry. **(a)** Representative gating strategy used to select YFP^pos^ CD4 TCRβ cells after eliminating doublet cells and dead cells for adoptive transfer of cells from the mLN **(b)** Cells were analyzed after isolation from the LP. Gating strategy used for YFP CD4 T cell analysis is illustrated **(c)** Gating strategy used to cell-sort the YFP^pos^ CD4 TCRβ cells subjected to scRNAseq, scATACseq, sc TCR seq and other chromatin analyses. Representative post sort purity efficacy is illustrated.

A fraction of invariant natural killer T cells, mucosal-associated invariant T cells and γδT cells express *Il17a* and thus can be targeted by the *Il17a-cre* system^14^. Taking into consideration that these IL-17^+^ invariant T cells are absent in the mLNs^18,19^, where the differentiation of naive cells into T_H_17 cells is mainly initiated^4^, we thus adoptively transferred NK1.1^–^CD4^+^T cell antigen receptor-αβ^+^ (TCRαβ^+^) cells from the mLNs of TGFβR-KO mice to *Rag2*-KO (RAG-KO) mice to create a source of T_H_17 cells escaping TGFβ signaling control (Fig. 1f and Extended Data Fig. 2a). Clearly, this adoptive transfer was sufficient to fully recapitulate the clinical signs found in TGFβR-KO mice, confirming that TGFβ signaling in T_H_17 cells is crucial to prevent them from initiating CIAC (Fig. 1f,g). Given the absence of spontaneous colonic transformation in TGFβR-KO mice, we evaluated the effects TGFβ signaling in T_H_17 cells when colonic transformation was already induced by exposure to the genotoxic compound azoxymethane (AOM) followed by dextran sodium sulfate (DSS). Strikingly, only one DSS treatment cycle was sufficient to induce invasive colonic adenocarcinoma in 33% of TGFβR-KO animals, whereas no colonic transformation was reported in TGFβR-WT mice (Supplementary Fig. 5). Of note, this oncogenic treatment, which classically targets the colon, was also sufficient to lead to duodenal/pyloric tubular adenoma at the bulb associated with small intestine inflammation in 67% of TGFβR-KO mice (Supplementary Fig. 5).

Thus, this dataset reveals that, once differentiated, TGFβ signaling prevents intestinal T_H_17 cells from becoming tumorigenic cells capable of triggering spontaneous transformation of the bulb or work in concert with exogenous genotoxic stress to rapidly promote both duodenal and colonic transformation.

### T_H_17 cells become tumorigenic through acquisition of a type 1 helper T (T_H_1) cell profile

**Fig. 2:**
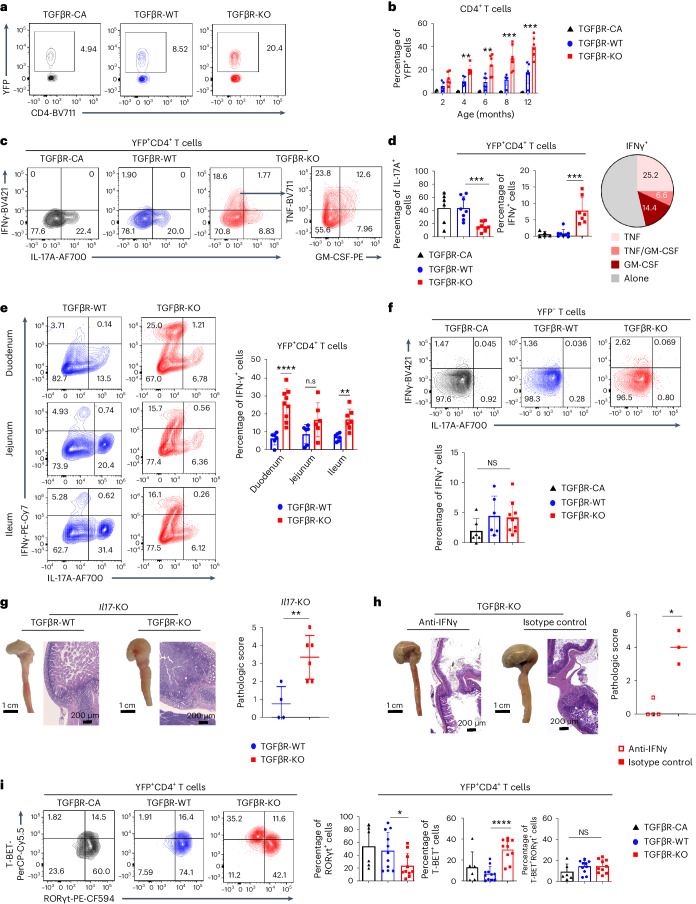
TGFβ signaling prevents development of T_H_17 cell-derived tumorigenic cells. TGFβR-CA, TGFβR-KO and TGFβR-WT mice were crossed with *Rosa26-stop*^fl/fl^*-yfp* reporter mice to map the fate of differentiated intestinal T_H_17 cells. Cells from the SILP were isolated and analyzed by flow cytometry. **a**, Representative contour plots of YFP expression in CD4^+^TCRβ^+^ cells from 8-month-old animals. **b**, Percentage of YFP^+^ cells among CD4^+^TCRβ^+^ cells of the SILP across the animal lifespan (mean ± s.d.). Data are representative of seven animals per group for each age analyzed from two independent experiments. **c**,**d**, Contour plots showing cytokine expression on YFP^+^CD4^+^ T cells from the SILP of 8-month-old mice (**c**). Quantifications (means ± s.d.) are shown (**d**). The pie graph in **d** illustrates the percentage of YFP^+^CD4^+^TCRβ^+^ cells expressing IFNγ either alone or in combination with other inflammatory cytokines. The experiment was repeated five times. **e**, Contour plots and bar graph (mean ± s.d.) illustrating the percentage of YFP^+^CD4^+^ T cells producing IFNγ and IL-17A in the lamina propria of different intestinal segments. The experiment was repeated four times. **f**, YFP^–^ T cells from the SILP of 8-month-old mice were analyzed by flow cytometry. Representative contour plots for cytokine expression and respective quantifications (mean ± s.d.) are shown. Data are representative of three independent experiments with three mice per group. **g**, *Il17a* expression was invalidated in TGFβR-KO and TGFβR-WT mice homozygous for *Il17a-cre*^14^. Representative gross lesions, duodenum H&E histology staining and pathology scores (mean ± s.d.) of 11-month-old animals are illustrated. The experiment was repeated five times. **h**, Neutralizing antibodies to IFNγ were injected in 4-month-old TGFβR-KO mice for 4 months. Representative gross lesions, duodenum H&E histology staining and pathology scores (mean ± s.d.) are illustrated. The experiment was repeated twice. **i**, Contour plots (left) showing the expression of TFs in YFP^+^CD4^+^ T cells from the SILP of 8-month-old mice and the respective quantifications (right; mean ± s.d.). Data are representative of three independent experiments with three mice per group. For all experiments, statistical significance was evaluated using two-tailed Student’s *t-*tests; *P < 0.05; **P < 0.01; ***P < 0.001; ****P < 0.0001; NS, not statistically significant. Source data

**Extended Data Fig. 3:**
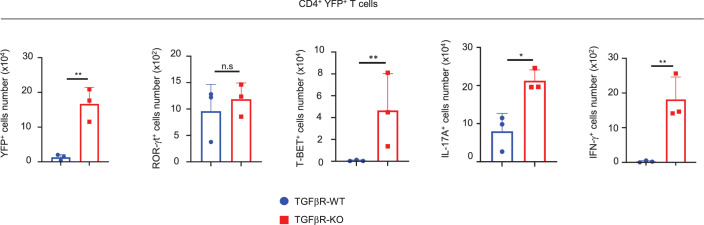
Absolute number of YFP^pos^ CD4 T cells. SILP of 8-month-old animals was analyzed by flow cytometry. Graph illustrates (mean +SD) the absolute numbers of YFP^pos^ cells expressing the indicated makers. Absolute numbers were normalized on tissue weight. Data are representative of 3 animals per group and 2 independent experiments. Statistical analysis was performed using a two-tailed Student’s t test * p<0.05, ** p<0.001 n.s no statistically different. Source data

Next, we assessed the consequences of the absence of TGFβ signaling on differentiated intestinal T_H_17 cells. To selectively track the outcome of these T_H_17 cells, TGFβR-KO, TGFβR-WT and TGFβR-CA mice were crossed with *Rosa26-stop*^fl/fl^*-yfp* mice. Strikingly, in both the SILP and CLP, CD4^+^TCRαβ^+^ cells expressing the yellow fluorescent protein (YFP^+^) were 2.5 and 5 times more abundant in TGFβR-KO mice than in TGFβR-WT mice and TGFβR-CA mice, respectively (Fig. 2a, Supplementary Fig. 2c,d and Extended Data Fig. 2b). This outnumbering of T_H_17-committed cells in TGFβR-KO mice was observed by 4 months of age, concomitantly with the first signs of chronic inflammation (Figs. 1c and 2b and Supplementary Fig. 2e). Of note, the YFP^+^ cells were highly concentrated at the bulb region of TGFβR-KO mice where transformation occurred (Supplementary Fig. 6a,b). As previously reported in the SILP of TGFβR-WT mice, around 25% of CD4^+^YFP^+^ T cells produced IL-17A, and 1% produced interferon-γ (IFNγ)^14^. By sharp contrast, in TGFβR-KO mice, both proportion and absolute numbers of YFP^+^CD4^+^ T cells producing IFNγ were increased by around 20 times (Fig. 2c,d and Extended Data Fig. 3). Of note, less than 2% of YFP^+^CD4^+^ T cells producing IFNγ co-produced IL-17A, revealing a complete switch from a T_H_17 to a T_H_1 cell profile in the SILP of TGFβR-KO mice. Notably, the ability of YFP^+^CD4^+^ T cells to produce IFNγ was two times more increased in the duodenum than in other intestinal segments, including the colon (Fig. 2e and Supplementary Fig. 2g). In the SILP, this IFNγ production by YFP^+^CD4^+^ T cells was associated with co-production of two other inflammatory cytokines, tumor necrosis factor (TNF) and granulocyte–macrophage colony-stimulating factor (GM-CSF; Fig. 2c,d and Supplementary Fig. 2f,g). Importantly, this exacerbated T_H_1 cell polarization was restricted to T_H_17-committed cells because YFP^–^ T cells exhibited similar IFNγ production in all mouse strains (Fig. 2f). Moreover, the analysis of T_reg_ cells failed to show any differences between TGFβR-KO and TGFβR-WT mice (Supplementary Fig. 7a,b), confirming that the exacerbation of the T_H_1 cell program was intrinsic to T_H_17 cells escaping TGFβ signaling control.

**Fig. 3:**
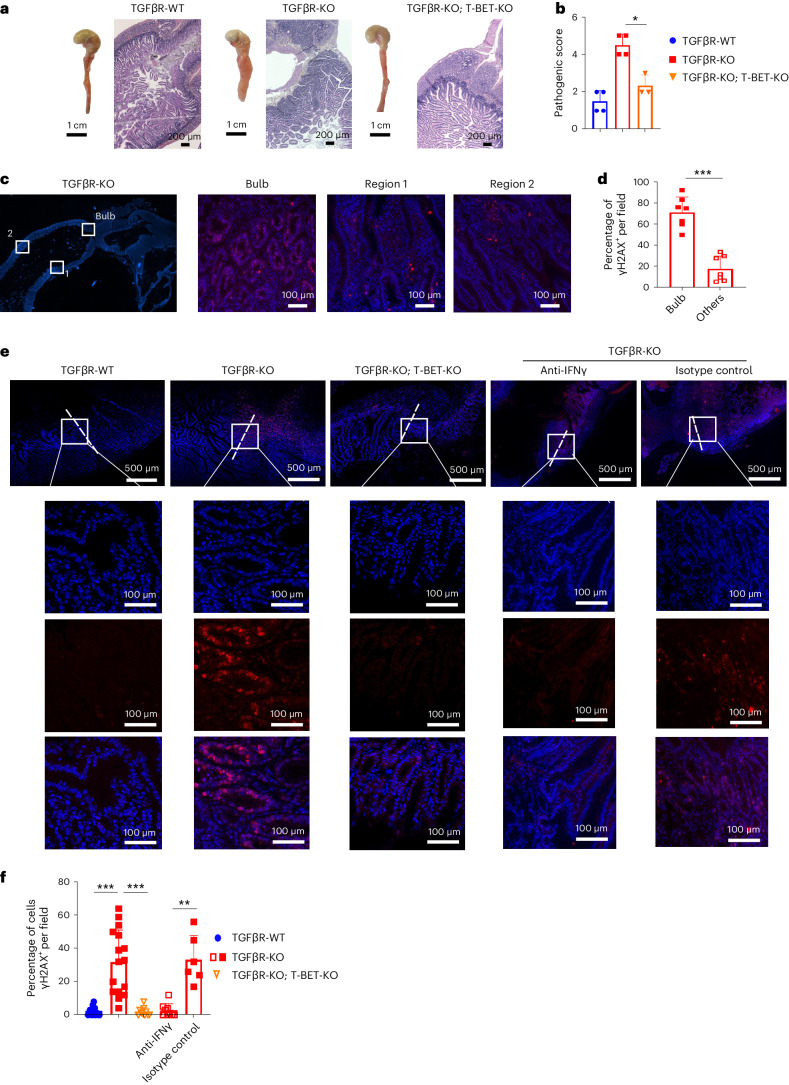
T_H_1 polarization of differentiated T_H_17 cells escaping TGFβ signaling initiates cancer. **a**, Representative gross lesions and H&E histology staining of the duodenum. **b**, Pathology score (mean ± s.d.). The experiment was repeated three times. **c**,**d**, Duodenums collected from 8-month-old TGFβR-KO mice were analyzed by immunostaining for γH2AX (red). **d**, Quantification of the percentage of γH2AX^+^ IECs per field in the duodenal bulb (mean ± s.d.). **e**,**f**, TGFβR-KO mice were treated with either neutralizing anti-IFNγ antibodies or isotope control. The duodenums of the different age-matched animals were analyzed by immunostaining for γH2AX (red). **f**, Quantification of the percentage of γH2AX^+^ IECs per field in the duodenal bulb (mean ± s.d.). The dashed line represents the limit between the duodenum and pyloric epithelium. Magnifications of the areas highlighted in white boxes are shown on the right. **d**, Quantification of the percentage of γH2AX^+^ IECs per field in the duodenal bulb (mean ± s.d.). Data are representative of three mice per group from two independent experiments. The experiment was repeated three times. Statistical significance was evaluated using a two-tailed Student’s *t*-test, except for **b** in which a two-tailed Mann–Whitney test was used; *P < 0.05; **P < 0.01; ***P < 0.001. Source data

Of note, the absolute number of intestinal YFP^+^CD4^+^ T cells producing IL-17A was three times higher in TGFβR-KO mice than in TGFβR-WT mice (Extended Data Fig. 3). However, duodenal CIAC was still detected when T_H_17-committed cells were unable to produce IL-17A (Fig. 2g), ruling out a key role for IL-17A and implying the importance of the switch to the T_H_1 cell program of T_H_17 cells for pathology development. Given that the exacerbated production of IFNγ was restricted to T_H_17 cells in TGFβR-KO mice (Fig. 2f), we proceeded to injections of anti-IFNγ neutralizing antibodies and found that this was sufficient to fully prevent CIAC in the duodenum of TGFβR-KO animals (Fig. 2h). In agreement with their increased IFNγ production, compared to TGFβR-WT mice, YFP^+^ cells of TGFβR-KO mice showed a fourfold increase in T-BET and a twofold decrease in RORγt expression, respectively (Fig. 2i and Supplementary Fig. 2h,i). Confirming the key role of the switch from a T_H_17 to T_H_1 cell program in pathology, in the absence of T-BET, no sign of CIAC was observed in the duodenum nor exacerbated transformation after AOM DSS genotoxic treatment in TGFβR-KO mice (Fig. 3a,b and Supplementary Fig. 5).

Given that double-stranded DNA (dsDNA) damage facilitates transformation during CIAC^1^, we next analyzed histone H2AX overphosphorylation (γH2AX) in the nucleus^20^. Clearly, TGFβR-KO mice showed an increase in γH2AX in IECs selectively at the bulb (Fig. 3c,d and Supplementary Fig. 2j), a phenotype that was totally absent after anti-IFNγ treatment and in the absence of T-BET in TGFβR-KO animals (Fig. 3e,f). Thus, these data reveal that, once differentiated, all, or some, of the intestinal T_H_17 cells must continuously receive TGFβ signaling to sustain the T_H_17 cell program and not switch to a proinflammatory T_H_1 cell program, dependent on T-BET, leading to IFNγ production responsible for dsDNA damage in IECs and cancer development.

### Characterization and origin of T_H_17 cell-derived tumorigenic T cells

**Fig. 4:**
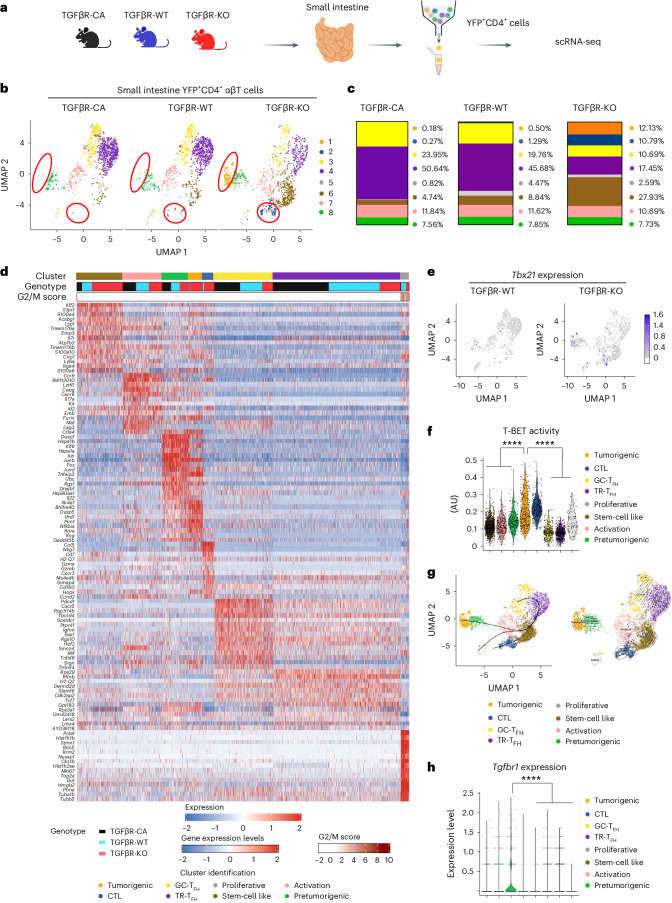
Characterization and origin of T_H_17 cell-derived tumorigenic cells. **a**, scRNA-seq analysis was performed on sorted YFP^+^CD4^+^ T cells isolated from the small intestines of 7- to 8-month-old TGFβR-CA, TGFβR-KO and TGFβR-WT mice as illustrated. **b**, Uniform manifold approximation and projection (UMAP) representation of the repartition of 1,000 cells per group. **c**, Proportion of each cluster identified among YFP^+^CD4^+^ αβT cells. **d**, Gene expression heat map representation of the top 15 genes increased and decreased in expression in each cluster from TGFβR-CA, TGFβR-KO and TGFβR-WT mice. **e**, UMAP representation of *Tbx21* expression in TGFβR-KO and TGFβR-WT mice. **f**, Violin plots illustrating T-BET regulon activity in the different clusters identified in TGFβR-KO mice; AU, arbitrary units; GC-T_FH_, germinal center-localized T_FH_ cells; TR-T_FH_, tissue-resident T_FH_ cells. **g**, Two different algorithms were used to evaluate the hierarchy between the single-cell clusters identified in intestinal YFP^+^CD4^+^ αβT cells isolated from TGFβR-KO mice. The slingshot package was used to infer pseudotime trajectories, depicted as three smoothed lines joining the different previously identified Seurat clusters (left). CellRank combines pseudotime and RNA velocity to provide streams of directionality (as shown by the arrows) across single-cell clusters (right). **h**, Violin plots demonstrating *Tgfbr1* expression in the different clusters identified in TGFβR-WT mice. For all experiments, statistical significance was evaluated using a two-sided Mann–Whitney test; ****P < 0.0001. Source data

**Extended Data Fig. 4:**
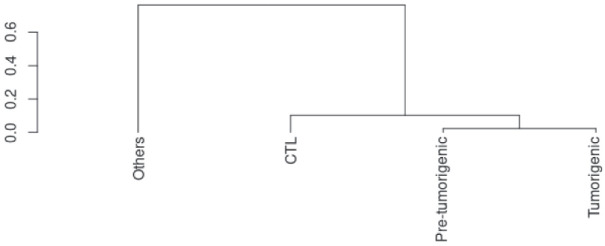
Comparison of TCR repertoire between pre-tumorigenic and tumorigenic cells. Sorted YFP^pos^ CD4 T cells isolated from the small intestine of 7-8-month-old, TGFβR-KO were analyzed by for TCR sequencing by scRNA. scRepertoire was used to assign clonotypes based on TCR chains, quantify and study clonotype dynamics, and integrate with gene expression data in combination with the Seurat package to redefine the cell subsets. Graph illustrates the hierarchical clustering on clonotype size and Jensen-Shannon divergence.

To better characterize the origin of T_H_17 cell-derived tumorigenic T cells, we next profiled YFP^+^CD4^+^TCRαβ^+^ cells isolated from the small intestine of TGFβR-CA, TGF-βR-WT and TGFβR-KO mice by droplet-based single-cell RNA sequencing (scRNA-seq; Fig. 4a). Overall, 802 genes were significantly up- or downregulated in TGFβR-KO versus in TGFβR-WT mice, and 389 genes were differentially expressed between TGFβR-CA and TGFβR-WT animals. Interestingly, after integration of transcriptome data from all three conditions, unsupervised clustering analysis divided the YFP^+^ cells into eight distinct subsets based on their transcriptional signatures, revealing a profound heterogeneity of the intestinal T_H_17 cell population (Fig. 4b). Strikingly, two clusters (clusters 1 and 2) present in TGFβR-KO mice and representing around 25% of YFP^+^CD4^+^TCRαβ^+^ cells were barely detectable in TGFβR-WT and TGFβR-CA mice (Fig. 4c). Cluster 1 was notably characterized by high levels of *Ifng*, *Bhlhe40*, *Furin* and *Nr4a1*, representing the T_H_17 cells that had acquired the T_H_1 cell program (Fig. 4d). Cytotoxic T lymphocyte (CTL)-associated genes, including *Gzma*, *Gzmb*, *Nkg7*, *Cd160*, *Cd107a* and *Ifng*, were expressed in cluster 2, and their CTL features was confirmed by flow cytometry (Fig. 4b–d and Supplementary Fig. 8). Of note, both *Tbx21* (encoding T-BET) expression and T-BET activity were largely restricted to both clusters 1 and 2 (Fig. 4e,f). However, the YFP^+^ CTL population and its ability to produce IFNγ remained present in the SILP of TGFβR-KO; T-BET-KO mice (Supplementary Fig. 8), which did not develop any pathology (Fig. 3a,b,e,f), implying that the CTL subset was not sufficient to initiate IEC transformation. Hence, we defined cluster 1 as the tumorigenic subset. In addition to T_H_1 cell features, the tumorigenic population was characterized by the expression of inhibitors of NF-κB signaling *Nfkbia*, *Pim1* and *Tnfaip3* (ref. ^21^), revealing their high level of activation, and the expression of *Hopx*, de novo expressed during CD4^+^ T cell transdifferentiation programs^22^. Gene expression analysis demonstrated that clusters 3 and 4 corresponded to T_H_17 cells converted into follicular helper T (T_FH_) cells. In agreement with the observations describing T_H_17 cells as the source of some T_FH_ cells in the intestine, including PP^23^, these data reveal that T_H_17 cells can give rise to both bona fide germinal center-localized T_FH_ cells (cluster 3), based on high expression of *Cxcr5*, *Bcl6*, *Pdcd1*, *Tnfrsf4*, *Icos* and *Maf*, and tissue-resident T_FH_ cells (cluster 4), which expressed lower levels of *Cxcr5* and *Blc6* but high levels of *Il7R*, *Itga4*, *Gpr183* and *Rflb* (Fig. 4d). Of note, the percentage of both T_FH_ subsets among intestinal YFP^+^ cells was halved in TGFβR-KO mice compared to in TGFβR-CA and TGFβR-WT mice, suggesting that the deprivation of TGFβ signaling in T_H_17 cells did not push their differentiation to one of the two T_FH_ subsets as reported in vitro^24^. However, in vivo, the effects of TGFβ signaling on the redifferentiation of T_H_17 cells in T_FH_ cells were not associated with changes in *Maf* expression (Fig. 4d)^24^. Cluster 5 corresponded to cells with a more proliferative phenotype, as demonstrated by their higher G2M score^25^ (Fig. 4d). This population was 2.5 to 5 times under-represented in TGFβR-CA mice compared to in TGFβR-KO mice and TGFβR-WT mice, respectively (Fig. 4b–d), in line with the lower numbers of YFP^+^CD4^+^ T cells observed in the intestines of TGFβR-CA animals (Fig. 2a). Cluster 6 included cells colonizing the SILP, as evidenced by the expression of *S1pr1*, *Klf2*, *Itga4*, *lsp1*, *S100a4/S100a6* and *Emp3*, and endowed with a stem cell-like signature (for example, *Slamf6*, *Il7r* and *Tcf7* (refs. ^26,27^); Fig. 4d). Along with their migratory stem cell-like phenotype, bioinformatic modelings indicated that all the subsets that we identified were initially derived from cluster 6 (Fig. 4g). In TGFβR-KO animals, cluster 6 was three and six times more abundant than in TGFβR-WT mice and TGFβR-CA mice, respectively. Hence, the proportions of clusters 5 and 6 in the different mouse strains strongly imply that the over-representation of YFP^+^ cells in the SILP of TGFβR-KO animals (Fig. 2a) was associated with an increased colonization of the SILP by recently differentiated T_H_17 cells rather than their proliferation. Cluster 7 contained activated intestinal T cells expressing high levels of *Il17a* and *Maf* and intestinal residency-associated genes *Ly6a*, *lztfl1*, *Smco4*, *Ccr9* and *Cxcr6*. Interestingly, in TGFβR-WT and TGFβR-CA animals, this activation cluster expressed higher levels of later activation makers, such as *Lag3*, *CtlA4* and *Pdcd1*, than TGFβR-KO mice (Fig. 4d), suggesting that the absence of TGFβ signaling could protect activated T_H_17 cells from a form of exhaustion. Finally, cluster 8 was characterized by high levels of *Hspa1*, *Hspa2*, *Ubc*, *Dusp1*, *Dnajb1*, *Jun* and *Fos*, implying that this subset underwent profound cellular stress. Strikingly, the different bioinformatic trajectory models that we used concluded that cluster 8 was derived from activated cells (cluster 7) and gave rise to the tumorigenic subset (cluster 1; Fig. 4g). Moreover, confirming this analysis, single-cell TCR repertoire analysis of cluster 8 and the tumorigenic population (cluster 1) revealed a higher similarity between these two populations than between the other subsets, including the CTL subset (cluster 2; Extended Data Fig. 4). Thus, we defined cluster 8 as a pretumorigenic population. The transition from the pretumorigenic to the tumorigenic stage was associated with the loss of cellular stress markers and an increase in T-BET activity (Fig. 4d,f). Importantly, in contrast to the tumorigenic subset, the pretumorigenic population was observed regardless of TGFβ signaling intensity in T_H_17 cells (Fig. 4b,c). Moreover, the gene expression pattern of pretumorigenic cells was very similar between TGFβR-CA, TGFβR-WT and TGFβR-KO animals (Fig. 4d), suggesting that TGFβ signaling could prevent pre-existing pretumorigenic T cells from becoming tumorigenic. Supporting this assumption, only the pretumorigenic population exhibited an exacerbated expression of *Tgfbr1* (Fig. 4h). Of note, we did not find any loss of *Il10*^+^ cells among the YFP^+^CD4^+^ T cells between the three types of mice, excluding a role for TGFβ signaling in controlling the size of the regulatory IL-10-producing T_H_17 cell population in the intestine (Supplementary Fig. 7c). Thus, once differentiated, a fraction of activated intestinal T_H_17 cells can reach a pretumorigenic state, but their ability to become tumorigenic is blocked by TGFβ signaling.

### KLF6 promotes development of tumorigenic cells

**Fig. 5:**
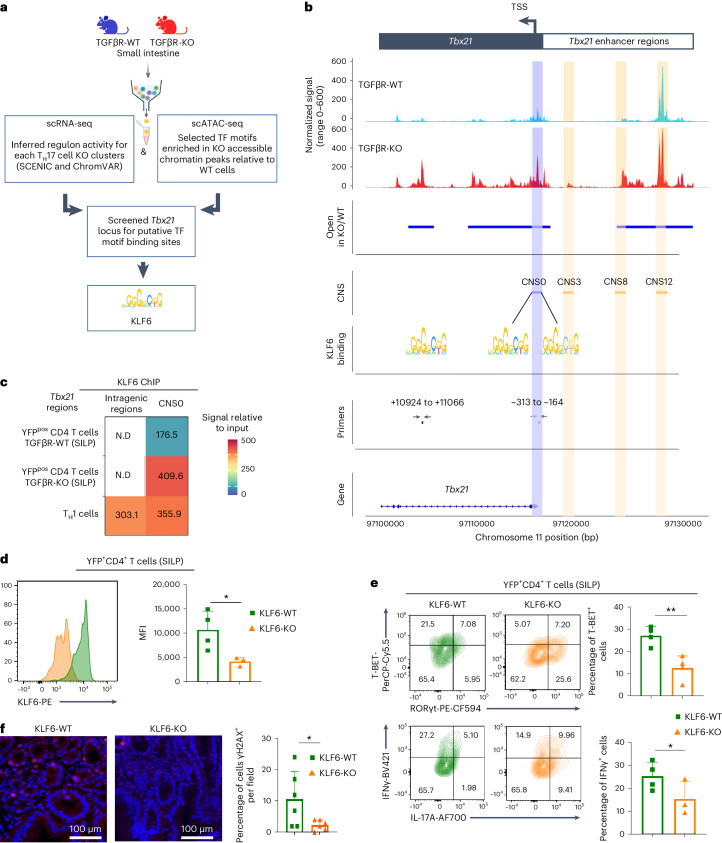
KLF6 promotes a tumorigenic state of T_H_17 cells. YFP^+^CD4^+^ T cells isolated from the small intestines of 7- to 8-month-old TGFβR-KO (KO) and TGFβR-WT (WT) mice were used for scRNA-seq, scATAC-seq, ChIP or CRISPR–Cas9 gene deletion. **a**, Workflow for the selection of TFs with *Tbx21* transactivation potential. After meeting two criteria (that is, significant regulon activity from scRNA-seq and motif enrichment from scATAC-seq), the final shortlist of TFs was based on the presence of putative *Tbx21* binding sites. **b**, scATAC-seq coverage plot neighboring the *Tbx21* transcription start site. The top two tracks indicate chromatin accessibility signals for TGFβR-WT and TGFβR-KO cells, with the ‘Open’ track representing the regions with differential chromatin accessibility between the two. Known CNSs and the newly described CNS0 are highlighted in orange and blue, respectively. Approximate locations of KLF6 binding motifs and primers are depicted with arrows; TSS, transcription start site. **c**, A ChIP assay for KLF6 binding on dedicated DNA regions of *Tbx21* was performed. The heat map represents the fold enriched signal compared to the input fraction. In vitro-differentiated T_H_1 cells from TGFβR-WT mice were used as a control; ND, not detectable. **d**–**f**, *Klf6* was deleted by CRISPR–Cas9 in purified YFP^+^CD4^+^TCRβ^+^ cells isolated from TGFβR-KO mice (KLF6-KO). Validation of the deletion was performed by flow cytometry (**d**). Cells were then adoptively transferred into C57BL/6 recipient mice. Representative contour plots of YFP^+^CD4^+^TCRβ^+^ cells (left) and bar graphs illustrating the percentage of T-BET^+^ and IFNγ^+^ cells 3 days after transfer (mean ± s.d.) are shown (**e**); MFI, mean fluorescence intensity. **f**, Representative γH2AX (red) and DAPI (blue) staining of the recipient bulb and histogram of the quantification of γH2AX^+^ IECs per field (mean ± s.d.). Data are representative of two independent experiments with three to four animals per experiment. For all experiments, statistical analyses were performed using unpaired *t*-tests; **P < 0.01; *P < 0.05. Source data

**Extended Data Fig. 5:**
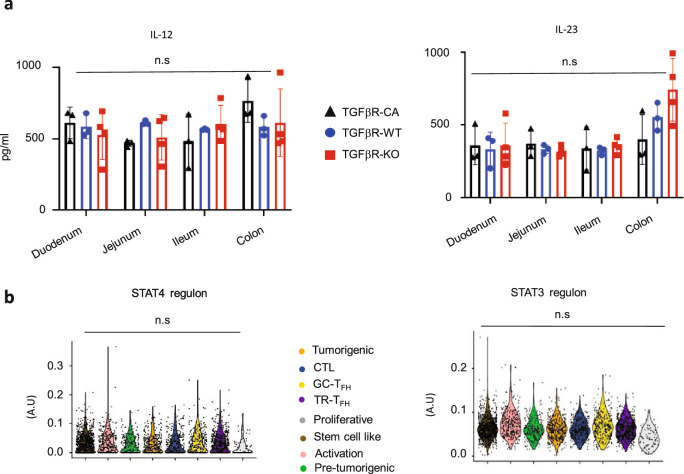
IL-23/12 levels in the intestine and IL-23/12 signature in tumorigenic T cells and pre-tumorigenic cells. **(a**) Graphs represent the concentrations (mean + SD) of cytokines in different intestinal segments of 7-8 month-old TGFβR-CA, TGFβR-KO and TGFβR-WT mice measured by ELISA. Concentrations were normalized per grams of tissue. ELISA data are representative of 2 experiments with 3–5 mice per group. (**b)** Sorted YFP^pos^ CD4 T cells isolated from the small intestine of 7-8 month-old TGFβR-KO mice were analyzed by scRNAseq as described in Fig. 4a. Violin plots representing STAT4 regulon and STAT3regulon in the different subsets. Statistical analysis was performed using a Mann-Whitney for the regulon and paired t test for ELISAs. n.s = non significatively different. Source data

Given the importance of T-BET expression for the acquisition of tumorigenic potential by differentiated T_H_17 cells (Fig. 3a,b,e,f), we next aimed to identify the upstream molecular regulators of *Tbx21* expression. During naive/T_H_1 cell differentiation, *Tbx21* expression is mainly induced by STAT4 activation in response to IL-12, which can also work with IL-23 to direct naive/T_H_17 differentiation toward a T_H_1 cell fate^28^. No differences in IL-12 and IL-23 levels, including in the duodenum, were detected between TGFβR-KO and TGFβR-WT mice (Extended Data Fig. 5a). In line with this observation, no exacerbated IL-12 and IL-23 signaling activity was observed in tumorigenic T_H_1 cell-like and pretumorigenic cells compared to in other cells from TGFβR-KO animals (Extended Data Fig. 5b), suggesting that, in the absence of TGFβ signaling, the acquisition of a T_H_1 cell program by intestinal T_H_17 cells obeys a distinct molecular mechanism to what has been described for naive cell transdifferentiation to T_H_1/T_H_17 cells. To decipher this molecular mechanism, we analyzed the *Tbx21* locus and found 191 motifs from 121 unique TFs (Supplementary Table 1). Using single-cell assay for transposase-accessible chromatin sequencing (scATAC-seq), we analyzed chromatin accessibility in YFP^+^CD4^+^TCRβ^+^ cells purified from the small intestine. Of the 746 TFs enriched in TGFβR-KO cells, only 54 had at least one motif match on the *Tbx21* locus (Supplementary Table 2). We then inferred regulon activity for the eight clusters from TGFβR-KO mice with scRNA-seq data using SCENIC. Of the 86 top tumorigenic regulons (Supplementary Table 3), only 3 were putative *Tbx21*-binding TFs with increased target chromatin accessibility, and only the TF Kruppel-like factor 6 (KLF6) was found to be in common. This result was confirmed with an alternative analysis of the scATAC-seq data using ChromVAR (Fig. 5a and Supplementary Table 4). Notably, *Klf6* was selectively expressed in both the pretumorigenic and tumorigenic subsets, and both *Klf6* expression and KLF6 regulon activity were increased in pretumorigenic TGFβR-KO cells compared to in control cells (Supplementary Fig. 9). Moreover, following the differentiation trajectory from the pretumorigenic to the tumorigenic stage (Fig. 4g), we found that both expression and activity of KLF6 preceded *Tbx21* expression (Supplementary Fig. 9). Of note, although TGFβR-WT pretumorigenic cells expressed *Klf6*, they exhibited only mild activity of this TF (Supplementary Fig. 9). This suggests that TGFβ signaling, known to be associated with various epigenetic regulators such as histone modifiers, DNA modifiers and nucleosome remodelers, controls DNA accessibility to KLF6 (ref. ^29^) in T_H_17 cells. In line with this scenario, *Tbx21* locus chromatin was more open in TGFβR-KO cells than in TGFβR-WT cells, including the promoter and enhancer regions (Fig. 5b). Hence, in the absence of TGFβ signaling, in pretumorigenic cells, all the required conditions could have been met (that is, KLF6 expression and KLF6 accessibility to *Tbx21* DNA) to allow *Tbx21* expression and progression to the tumorigenic state. In agreement with this hypothesis, we found three putative KLF6 binding sites on *Tbx21*, all located in accessible DNA regions, in TGFβR-KO compared to in TGFβR-WT cells. Two of these three binding sites were restricted to a 149-base pair (bp) region that we named conserved noncoding sequence 0 (CNS0), in relation to the CNS3, CNS8 and CNS12 regions recently depicted^30^, whereas the third binding site was in the intragenic region (Fig. 5b). A chromatin immunoprecipitation (ChIP) assay on intestinal YFP^+^CD4^+^ T cells confirmed that KLF6 efficiently bound CNS0 in TGFβR-KO cells (Fig. 5c). Interestingly, KLF6 did not bind to the intragenic region of *Tbx21*, whereas it bound both the CNS0 and the intragenic region in differentiated TGFβR-WT T_H_1 cells, suggesting a different mode of regulation of *Tbx21* expression by KLF6 in T_H_17 cell-derived T_H_1 cells, and naive/T_H_1 cell differentiation also repressed by TGFβ1 signaling^31^. Demonstrating the role of KLF6 in the induction of *Tbx21* expression in intestinal YFP^+^CD4^+^ TGFβR-KO T cells and thus in pathology, deletion of *Klf6* in these cells was sufficient to prevent both T-BET and IFNγ expression as well as dsDNA damage in the bulb (Fig. 5d–f). Hence, these data identify KLF6 as a key TF capable of sustaining high levels of T-BET expression in differentiated T_H_17 cells, which is essential for developing the tumorigenic population.

### IEC-produced TGFβ1 prevents T_H_17 cells from becoming tumorigenic

**Fig. 6:**
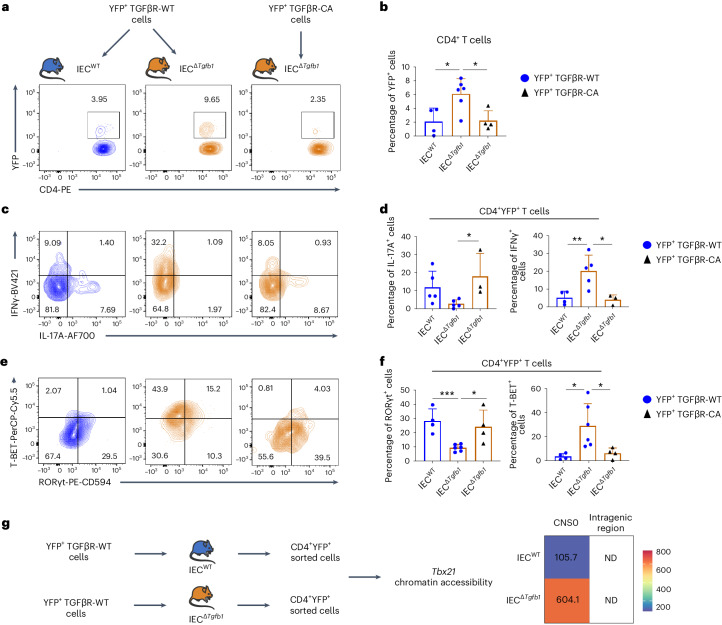
IEC-produced TGFβ1 prevents differentiated T_H_17 cells from becoming tumorigenic. YFP^+^ T cells from TGFβR-WT or TGFβR-CA mice were transferred into either IEC^Δ*Tgfb1*^ or *Villin-cre*, *Tgfb1*^WT^ (IEC^WT^) recipient mice. Recipients were then treated with tamoxifen, and CD4^+^TCRβ^+^ cells from the SILP were analyzed 3 weeks after transfer. **a**,**b**, Representative flow cytometry contour plots (**a**) and percentages of YFP^+^ cells among CD4^+^ T cells (**b**). **c**–**f**, Representative flow cytometry contour plots of cytokine production and IFNγ and TF factor expression (**c** and **e**) as well as quantifications (mean ± s.d.; **d** and **f**). **g**, DNA from YFP^+^CD4^+^ T cells purified from the SILP of either IEC^Δ*Tgfb1*^ or IEC^WT^ recipient mice were analyzed for chromatin accessibility of both KLF6 binding regions (CNS0 and the intragenic regions) of the *Tbx21* locus. The heat map demonstrates the fold enriched signal compared to the input fraction. Data are representative of three to six mice from three independent experiments. For all experiments, statistical analyses were performed using a two-tailed Student’s *t*-test; *P < 0.05; **P < 0.01; ***P < 0.001. Source data

**Extended Data Fig. 6:**
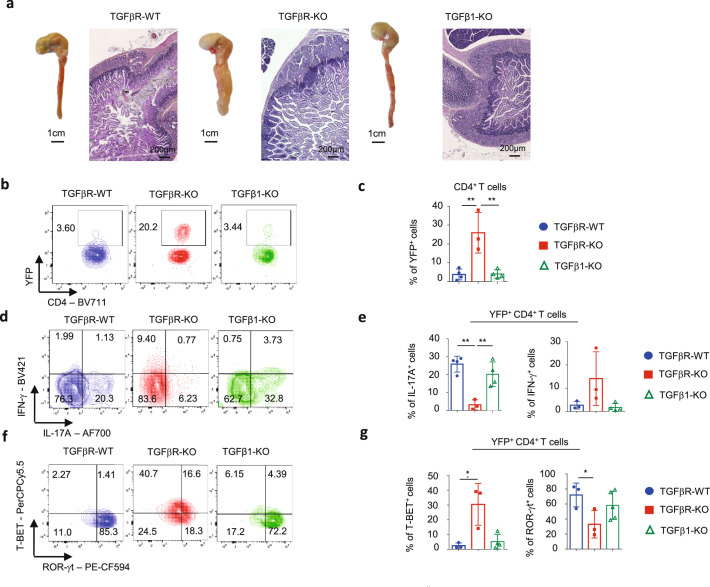
Analysis of TGF-β1 autocrine production by T_H_17 cells on tumorigenic state development. Experiments were performed using *Il17a-Cre; Rosa stop*^fl/fl^ *Yfp* -TGFβR-KO and TGFβR-WT mice invalidated for *Tgfb1*. **(a)** Representative gross lesions and H&E histology staining of the duodenum **(b, c)** Representative counter plots and quantification (mean +SD) of the percentage of YFP^pos^ CD4 T cell population in the SILP. **(d-g)** YFP^pos^ CD4 T cells were analyzed for cytokine production and TF expression. Representative counter plots are illustrated as well as graphs illustrating the percentage of cells (mean +SD) expressing the mentioned proteins. Data are representative of 2 experiments 3 to 5 mice per group. Statistical analysis was performed using a two-tailed Student’s t test. *P<0.05; **P<0.01. Source data

**Extended Data Fig. 7:**
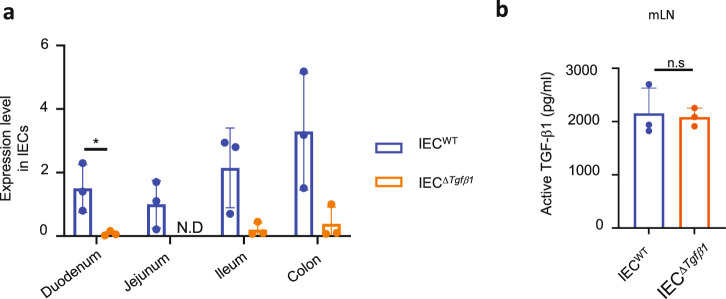
Confirmation of *Tgfb1* deletion without effect on TGF-β activity in mLN. *VillinCRE*^*ERT2*^*, Tgfb1*^*fl/fl*^ (IEC^Δ*Tgfb1*^*)* mice with tamoxifen inducible deletion of *Tgfb1* in IECs were used. (**a**)mRNA was extracted from purified of IEC isolated from IEC^Δ*Tgfb1*^and IEC^WT^ mice isolated from different intestinal segments. *Tgfb1* expression IECs was evaluated by qRT-PCR normalized on *Gapdh* expression. **(b**) mLN were harvested and the levels of TGF-b1 was measured by ELISA. Data are representative of 3 animals per group from two independent experiments. Source data

Finally, we aimed to identify the cellular source of TGFβ1 in the intestine preventing tumorigenic T_H_17 cell development. We excluded a role of the autocrine TGFβ1, previously identified as essential for the differentiation of T_H_17 cells from naive cells^32^ because TGFβ1-KO mice never showed any pathologic signs nor exacerbated reprogramming of T_H_17 cells to a T_H_1 cell fate (Extended Data Fig. 6). Because IECs are considered an important source of TGFβ1 in the gut^33^, and immunostaining revealed that YFP^+^CD4^+^ T cells were predominantly localized in the villi and were thus surrounded by IECs (Supplementary Fig. 6c), we analyzed the effects of IEC-derived TGFβ1 on the switch from intestinal T_H_17 cells to T_H_1 cells. We transferred YFP^+^CD4^+^ T cells from the small intestines of TGFβR-WT mice to *Villin-cre*^ERT2^; *Tgfb1*^fl/fl^ (IEC^Δ^^*Tgfb1*^) animals, reducing TGFβ1 levels in all intestinal segments but not in the mLNs after tamoxifen treatment (Extended Data Fig. 7). To confirm the direct role of IEC-produced TGFβ1 on differentiated T_H_17 cells, we also transferred TGFβR-CA T cells, which maintain TGFβ signaling independent of the presence of TGFβ1 in their microenvironment^16^, into IEC^Δ^^*Tgfb1*^ animals (Fig. 6a). Similar to TGFβR-KO mice, 5 weeks after transfer, the pool of intestinal TGFβR-WT YFP^+^CD4^+^ T cells was two to three times higher in IEC^Δ^^*Tgfb1*^ animals than in control mice (Figs. 2c–f and 6a,b), and the absence of TGFβ1 production by IECs was sufficient to induce both IFNγ and T-BET expression and conversely impaired IL-17A and RORγt expression in differentiated T_H_17 cells (Fig. 6c–f). Importantly, deprivation of TGFβ1 in the intestines of IEC^Δ^^*Tgfb1*^ mice was not associated with an increase of T_H_1 cell reprogramming in TGFβR-CA-derived YFP^+^CD4^+^ T cells (Fig. 6c–f). Moreover, in agreement with the acquisition of this tumorigenic phenotype by T_H_17 cells, the absence of IEC-derived TGFβ1 was sufficient to increase the accessibility of CNS0 of the *Tbx21* locus in intestinal YFP^+^CD4^+^ T cells (Fig. 6g). Thus, through the production of TGFβ1, IECs avoid the progression of differentiated T_H_17 cells to the tumorigenic state, revealing a key cellular interplay in the prevention of intestinal CIAC.

## Discussion

The ability of T_H_17 cells to exert both beneficial and pathogenic functions under different conditions and tissue environments has recently suggested that this effector T cell type could be heterogenous^12^. This study reveals that, in the small intestine, the differentiated T_H_17 population is actually composed of eight distinct subsets. Among them, we defined a pretumorigenic subset whose progression to a tumorigenic state leads to CIAC with spontaneous adenocarcinoma formation, revealing that effector T cells can induce tissue transformation.

The transition from pretumorigenic cells to tumorigenic cells is prevented by TGFβ signaling. Hence, in addition to playing a key role in the differentiation of naive cells to T_H_17 cells^34^, TGFβ signaling must be sustained in T_H_17 cells once they are differentiated to avoid CIAC. The signaling pathway and the main cellular source of TGFβ involved in naive/T_H_17 differentiation are different from those controlling the outcome of differentiated T_H_17 cells. Indeed, it is not via the canonical branches of TGFβ signaling (that is, SMAD4 and TRIM33) but likely through its noncanonical branch that TGFβ signaling prevents differentiated T_H_17 cells from becoming tumorigenic T_H_1 cells. This SMAD4/TRIM33-independent branch of TGFβ signaling also prevents naive CD4^+^ T cell differentiation in T_H_1 cells^35^, suggesting its specific role in repression of the T_H_1 cell program irrelevant of the naive or already differentiated status of the CD4^+^ T cells. IEC-produced TGFβ1 appears at the core of the interplay between IECs and differentiated intestinal T_H_17 cells to protect the host from the generation of tumorigenic T cells and CIAC. High levels of TGFβ1 in the intestine, provided by, in large part, numerous IECs, may be required to activate the noncanonical pathway of TGFβ signaling. Interestingly, IECs have also been reported to contribute to differentiation of naive T cells to the T_H_17 cell fate, particularly by the production of SAA1/SAA2 (ref. ^36^). Hence, IECs contribute to both early and late stages of T_H_17 cell biology with direct consequences on intestinal epithelial barrier integrity. First, by promoting the generation of T_H_17 cells, IECs sustain their tight junctions and strengthen the epithelial barrier^37^. Second, by preventing tumorigenic T_H_17 cell development, they avoid CIAC.

The tumorigenic population develops from a pretumorigenic population, which likely composes an intermediate state between activated T_H_17 cells and tumorigenic cells. The high cellular stress signature observed in the pretumorigenic subset and the trajectory modelings strongly imply that the pretumorigenic state could be a final state of differentiation potentially associated with cell death when TGFβ signaling in T_H_17 cells is sustained. The TCR repertoire of pretumorigenic cells was very close to that of the tumorigenic population but was not fully identical, suggesting that only some clones could get an advantage in the absence of TGFβ control. Further investigations, including research on antigen specificity of tumorigenic cells, should clarify this aspect. Similar to humans^17^, the proximal duodenum is the principal region of transformation malignancy in TGFβR-KO mice. Several factors could explain this specific localization. The proximal part of the duodenum has a distinct embryonic origin versus the rest of the duodenum and the small intestine, which may be responsible for the expression of specific antigens to which T_H_17 cells escaping TGFβ control could react, explaining the high density of YFP^+^ cells at the bulb. Alternatively, or concomitantly, specific environmental factors of the proximal duodenum, such as the high concentration of biliary acids and antigens delivered by the common bile duct, could contribute to the localization of pathology. This idea is reinforced by the extremely rapid and surprising formation of duodenum adenocarcinoma in TGFβR-KO mice after exposure to AOM, a molecule transformed in the liver that becomes genotoxic and is delivered to the small and large intestine through the common bile duct^38^. Tumorigenic T cells promote transformation in the colon but, in contrast to the duodenum, only after AOM exposition, reinforcing the idea that the duodenum and the colon constitute two distinct environments for the oncogenic capacity of tumorigenic T cells. In the duodenum, they induce malignant transformation, whereas in the colon, they promote the effects of exogenous genotoxic agents.

**Extended Data Fig. 8:**
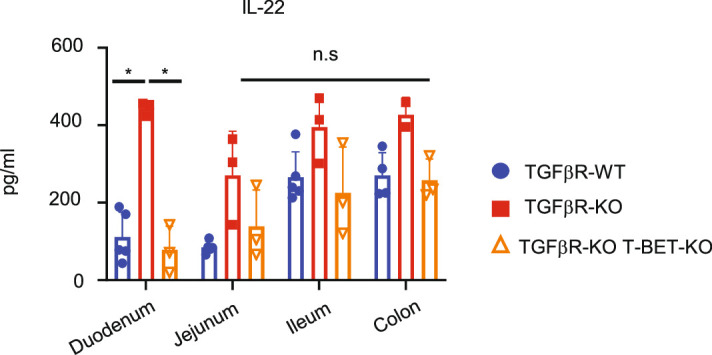
Analysis of the IL-22 levels in the intestine. Graph demonstrates the concentration of IL-22 in different intestinal segments of TGFβR-WT, TGFβR-KO, TGFβR-KO T-BET-KO mice (mean + SD). Data are representative of 2 experiments with 4 mice per group. Statistical analysis was performed using unpaired Student’s t test * p<0.05. n.s no statistically different. Source data

The IL-17/T_H_17 cell axis has been largely proposed to either promote or repress the growth of already established tumors^39,40^. By showing that T_H_17 cells can also initiate malignant transformation, this study assigned an unexpected role for T_H_17 cells and effector T cells in general in cancer initiation, opening the path to prophylactic treatments and questioning potential side effects for therapies stimulating T cells. It is clear that CIAC is a multiple factor phenomena that includes innate immune cells, which could play a part in CIAC initiated by T_H_17 cells escaping TGFβ control, linking both arms of the immune system in cancer initiation. To initiate cancer, T_H_17 cells do not need to produce IL-17A. However, we cannot exclude that other cellular sources of IL-17A could contribute to pathology. Our data showed that T_H_17 cell reprogramming in T_H_1 cells and associated IFNγ production are essential and sufficient to induce dsDNA damage in IECs and the subsequent transformation of cells chronically exposed to genotoxic stress. IFNγ has been proposed to induce oxidative stress, particularly NOX4, and dsDNA damage in vitro^41^. Further investigations should determine whether in vivo IFNγ genotoxic effects on duodenal epithelial cells also involves oxidative stress and/or other factors, particularly in villi stem cells present at the crypts, where YFP^+^CD4^+^ T cells were also highly abundant in the duodenum of TGFβR-KO mice. IL-22 has been reported to facilitate the repair of dsDNA damage in villi stem cells in response to genotoxic stress^42^. Interestingly, *Il22*, largely expressed by pretumorigenic cells, is one of the few genes downregulated in expression in TGFβR-KO mice compared to in TGFβR-WT mice, suggesting that lower levels of *Il22* expression by TGFβR-KO pretumorigenic T cells may contribute to cancer progression once dsDNA damage is generated. Interestingly, the levels of IL-22 were three times higher in the duodenum of TGFβR-KO mice than in TGFβR-WT animals (Extended Data Fig. 8), implying that cells other than T_H_17 cells provide a source of IL-22, such as type 3 innate lymphoid cells, reported to repair dsDNA after chemical-induced genotoxicity^42^. However, the impact of these IL-22-producing cells seemed inefficient to repair dsDNA damage caused by tumorigenic T cells, underlying the strong transforming potential of the latter.

Several members of the Krüppel-like factor family, including KLF2, KLF4, KLF10 and KLF13, have been shown to be expressed in T cells and involved in thymic development and migration and effector or regulatory functions^43^. The role of KLF6 in CD4^+^ T cells has been limited so far to correlations with an activation/memory phenotype^44^. This study identified KLF6 as a master regulator of tumorigenic T cell development. KLF6-dependent *Tbx21* expression is required to differentiate T_H_17 cells to the tumorigenic subset. KLF6 binds to the CNS0 region of the *Tbx21* promoter and promotes T-BET expression crucial for progressing pretumorigenic cells to the tumorigenic state. The pretumorigenic cells present in the intestines of TGFβR-WT animals express *Klf6* at low levels, suggesting that they are prompt to become tumorigenic. However, TGFβ1 and, in particular, IEC-derived TGFβ blocks CNS0 accessibility to KLF6 in intestinal T_H_17 cells, keeping the tumorigenic program in check. We propose KLF6 as a key TF for the continuum of differentiation of T_H_17 cells to the tumorigenic state leading to CIAC.

Although further clinical studies should be performed to validate the presence of the tumorigenic subset as a predictive marker for intestinal cancer susceptibility, several arguments are in favor of a role for tumorigenic T_H_17 cells in CIAC in the human small intestine. First, the vast majority of tumors of the small intestine in humans are localized at the same site where tumorigenic T cells induce transformation in mice^17^. Second, *TBX21* also contains the CNS0 KLF6 binding site (Supplementary Fig. 10a). Third, scRNA-seq analysis on human T_H_17 cells showed that *KLF6* is expressed in T_H_17 cells in individuals with small intestinal chronic inflammation such as Crohn’s disease but not in other T_H_17 cell-mediated pathologies such as multiple sclerosis or ulcerative colitis (Supplementary Fig. 10b). Finally, using genome-wide association studies analyses of individuals with chronic inflammatory bowel disease, others have suggested KLF6 as a candidate for T_H_17 cell-specific mediation of chronic gut inflammation in humans^45^.

In summary, our work shows a mechanism that protects against intestinal cancer initiation. This mechanism is based on an interplay between IECs and differentiated T_H_17 cells, which blocks the continuum of differentiation of pretumorigenic T_H_17 cells to a tumorigenic state, protecting IECs against malignant transformation. By identifying TGFβ1 in this preventive mechanism against cancer, this study questions the suitability of cancer immunotherapy strategies that are based on systemic TGFβ1 targeting to restore efficient T cell cytotoxic functions against cancer cells^46^.

## Methods

### Ethics

Experiments in mice were performed in accordance with the animal care guidelines of the European Union ARRIVE and French laws and were validated by the local animal ethics evaluation committees CECAPP and the French Ministry of research under approbation numbers CECAPP CLB 2017‐017 and APAFIS 18685. No animal developed intestinal inflammation/tumors reaching a state that could alter their daily lives in any manner. For human data reanalysis, ethics statements were reported in the original published articles^47–49^.

### Mice

*Il17a‐cre* mice were provided by G. Stockinger (The Francis Crick Institute)^14^, and *Vil1-cre*^Ert2^ mice were provided by S. Robine (Institut Pasteur)^50^. Stop^fl/fl^-*Tgfbr1*-CA mice were generated as previously described^16^. *Tgfbr2*^fl/fl^ mice were provided by S. Karlsson (Lund university)^15^, *Rosa26‐stop*^fl/fl^*-yfp* mice were provided by F. Constantini (Columbia University)^51^, *Trim3*^fl/fl^ mice were provided by R. Losson (Ilkirch), *Smad4*^fl/fl^ mice were provided by C. Deng (NIH)^52^, C.129S6-*Tbx21*^*tm1Glm*^/J (T-BET-KO) mice were provided by L. Glimcher (Havard University) and *Tgfb1*^*tm2.1Doe*^*Tgfb1*^fl^ mice were provided by O. Sansom (Glasgow University)^53^. *Il17a*-KO animals were obtained from homozygous *Il17a‐cre* animals using a knock-in approach after insertion of the construct in both *Il17a* alleles^14^. RAG-KO (B6.Cg-*Rag2*^tm1.1Cgn^/J) mice (008449, Jackson Laboratory) and C57BL6/J mice (000664, Jackson Laboratory) were purchased from Charles River, and colonies were maintained. Except when mentioned, experiments were performed on 6- to 8-month-old mice. All mice were on a C57BL/6 background, and both sexes were used without differences between males and females being observed except for AOM DSS, in which males were used because they responded more strongly. Littermates without floxed alleles were used as WT control mice. Except for the microbiota analysis, TGFβR-WT and TGFβR-KO animals were cohoused throughout their lifespan. All animals were maintained in specific pathogen‐free animal facilities (Small Animal Platform/Animal Core Facility/Imaging Platform or RAM-ZEFI). Irradiated diet chow (2918 Envigo; A04 and 150 SP-25 Safe Diets) was provided without any alteration in our observations. Animals were housed with enrichment media, including light shed houses and cotton squares, on a 12-h light/12-h dark cycle in a controlled environment (temperature of 22 + 1 °C and hygrometry of 50–60%).

### AOM DSS treatment

Mice were injected intraperitoneally (i.p.) with AOM (6.25 mg per kg (body weight); 25843-45-2, Sigma-Aldrich), followed 30 days later by a single 5-day period of 2.5% (wt/vol) DSS (TdB) administered in the drinking water. Because TGFβR-KO mice did not regain weight after DSS treatment, no other induction of inflammation was performed, as is the case in the classical colitis-associated carcinogenesis protocol^54^. Mouse weight was measured every other day, and when an over 20% weight loss was observed, mice were killed. Animals were killed after 80 days, and organs were collected for histological analysis.

### Tamoxifen and anti-IFNγ treatment

Mice were injected i.p. every day over 5 days with 100 μl of a 10 mg ml^–1^ tamoxifen solution (T5648, Sigma-Aldrich) dissolved with pure ethanol and diluted in corn oil (C8267, Sigma-Aldrich), allowing Cre activity in the intestine for 60 days^50^. For anti-IFNγ treatment, mice were injected i.p. every 3 days with 200 µg of either neutralizing anti-IFNγ (XMG1.2, BioXCell) or IgG isotype control (HRPN, BioXCell).

### Isolation of intestinal cells

The small intestine and colon were dissected, and fat was removed. Intestines were longitudinally opened and washed in 1× PBS (14200091, Gibco). Intestines were cut into small pieces and incubated with 5 mM EDTA (EU0084, Euromedex) and 1 mM DTT (D0632, Sigma-Aldrich) at 37 °C. Epithelial cells were then separated from intraepithelial lymphocytes with a 44%/67% Percoll gradient (P1644, Sigma-Aldrich) run for 20 min at 1,300*g*. Tissues were then digested in RPMI medium (618700044, Gibco) containing 20% fetal bovine serum (FBS; 10437028, Gibco), 100 µg ml^–1^ DNase I (DN25-IG, Sigma-Aldrich) and 1 mg ml^–1^ collagenase from *Clostridium histolyticum* (C2674, Sigma-Aldrich). Intestinal lamina propria lymphocytes were then separated on a 44%/67% Percoll gradient run for 20 min at 1,300*g*.

### Flow cytometry and cell sorting

Surface staining was performed using the following fluorescence-conjugated antibodies diluted in 1× PBS (Gibco) containing 2% bovine serum albumin (A7906, Sigma-Aldrich) and 0.1% sodium azide (08591, Sigma-Aldrich): CD45-APC-Cy7 (30-F11, BD Biosciences), TCRβ-PE (H57-597, BD), CD3-BV650 (145-2C11, BD Biosciences), CD4-BV711 (RM4-5, Biolegend), CD8-BV510 (53-6.7, BD Biosciences) and CRTAM (11-5/CRTAM-PE, Biolegend). For intracellular cytokine staining, mouse cells were restimulated ex vivo for 4 h with 500 ng ml^–1^ PMA (P1585, Sigma-Aldrich) and 500 ng ml^–1^ ionomycin (I0634, Sigma-Aldrich) in the presence of brefeldin A (BD Biosciences). Cells were stained for surface markers and fixed for 30 min with 1% paraformaldehyde (PFA; 11481745, Fisher Scientific) in 1× PBS (1420091, Gibco) and permeabilized with a Cytofix/Cytoperm kit (554655, BD Biosciences) according to the manufacturer’s protocol. Intracellular staining was performed for IL-17A-AlexaFluor 700 (TC11-18H10, BD Biosciences), IFNγ-APC (XMG1.2, BD Biosciences), GM-CSF-PerCP-Cy5.5 (MP1-22E9, Biolegend), TNF-PE-Cy7 (MP6-XT22, BD Biosciences) and granzyme B-APC (GB11, Invitrogen). For intranuclear staining, cells were treated with a nuclear fixation kit (Ebioscience) before being stained with RORγt-PE-CF594 (Q31-378, BD Biosciences), T-BET-APC (eBio4B10, Ebioscience), granzyme B-APC (GB11, Invitrogen) and KLF6-PE (E-10, Santa Cruz). For p-SMAD2/SMAD3 staining, after a 15-min incubation with 5 ng ml^–1^ activated recombinant TGFβ1 (240-B010, R&D Systems) at 37 °C, cells were immediately fixed with a Fixation and Permeabilization Buffer kit (00-5523-00, eBioscience) and stained with anti-p-SMAD2/SMAD3 (D27F4, Cell Signaling) detected with a donkey anti-rabbit APC secondary antibody (A31573, Life Technology). All samples were acquired on a BD Fortessa except for samples processed with the CRISPR–Cas9 approach and used for intestinal segment analysis, for which an AURORA Cytek machine was used. Analyses were performed with FlowJo v10.6.1 software (BD Biosciences). For cell sorting, CD4^+^ T cells were enriched using a CD4^+^ T Cell Isolation kit (mouse; 130-104-454, Miltenyi Biotec) and labeled with CD4-PE (GK1.5, eBioscience), TCRβ-APC (H57-597, BD Biosciences), CD45-APC-Cy7 (30-F11, BD Biosciences) and DAPI (Eurobio Scientific). All antibodies were used at 1:200 except RORγt-PE-CF594 (used at 1:400) and p-SMAD2/SMAD3 and CRTAM (both used at 1:100). Cells were purified on an Aria II (BD Biosciences) according to Extended Data Fig. 2.

### Adoptive T cell transfer

Sorted CD4^+^TCRβ^+^NK1.1^–^ cells (1 × 10^6^) isolated from the mLNs of either TGFβR-WT or TGFβR-KO mice were injected intravenously (i.v.) into RAG-KO mice. After electroporation with CRISPR–Cas9 and guide RNA (gRNA), purified YFP^+^CD4^+^TCRβ^+^ cells (1 × 10^5^) from the SILP of TGFβR-KO mice were injected i.v. into C57BL/6 WT mice. Sorted YFP^+^CD45^+^CD4^+^TCRβ^+^ cells (5 × 10^4^) isolated from the small intestines of either TGFβR-WT or TGFβR-KO mice were injected i.v. into either IEC^Δ*Tgfb1*^ or IEC^WT^ recipients.

### Histology and pathology scoring

The small intestine and colon were fixed in 4% PFA in PBS overnight and maintained in 70% ethanol diluted in distilled water. Samples were embedded in paraffin, sliced into 5-μm-thick sections, mounted and stained with H&E using standard protocols. All microscopy acquisitions were performed on a Zeiss Axio Imager M2 and visualized with a NanoZoomer slide scanner controlled by NDP.view software. Scoring of histopathology was performed blinded using the method described by el Marjou et al.^50^. Briefly, for all pathologic scoring, the following eight parameters were used: (1) the degree of inflammatory infiltrate in the lamina propria, ranging from 1 to 3; (2) the loss of Goblet cells, ranging from 0 to 2; (3) epithelial hyperplasia, ranging from 0 to 4; (4) cryptitis, ranging from 0 to 2; (5) number of crypt abscesses, ranging from 0 to 3; (6) extent of crypt loss regions, ranging from 0 to 2; (7) mucosal erosion to advanced ulceration, ranging from 0 to 4; and (8) presence of adenoma, ranging from 5 to 6. The severity of the inflammatory changes in the distal colon and duodenum correspond to the addition of the scores reported for each parameter.

### Intestinal microbiota analysis

Microbial DNA from 200 mg of fresh stools of 3-month-old TGFβR-WT and TGFβR-KO mice was extracted by GenoScreen. Microbial diversity and composition were determined for each sample by targeting a portion of the ribosomal genes. A 16S rRNA gene fragment comprising V3 and V4 hypervariable regions (16S; 5′-TACGGRAGGCAGCAG-3′ and 5′-CTACCNGGGTATCTAAT-3′) was amplified using an optimized and standardized 16S amplicon library preparation protocol (Metabiote v2.0, GenoScreen). Sequencing was performed using a 250-bp paired-end sequencing protocol on an Illumina MiSeq platform (Illumina) at GenoScreen. Positive (artificial bacteria community comprising eight different bacteria (‘ZymoBIOMICS’)) and negative (sterile water) controls were also included. Raw paired-end reads were processed in a data curation pipeline that included a step to remove low-quality reads (Qiime2 2020.8). The remaining sequences were assigned to samples based on barcode matches, and barcode and primer sequences were then trimmed. The sequences were denoised using the DADA2 method, and reads were classified using the Silva reference database (version 138). The α- and β-diversities were computed. Chao1 and Shannon indexes were calculated to characterize α-diversity, and principal coordinate analyses of the Bray Curtis distance and the unweighted UniFrac distance were performed to assess β-diversity using Qiime2 2020.8. Chao1 and Shannon indexes were calculated to characterize α-diversity.

### T_H_1 cell differentiation

CD4^+^ T cells were purified from mLNs (130-104-454, Miltenyi Biotec.). In total, 2 × 10^5^ cells were activated in a 96-well Nunc plate (Thermo Fisher) in the presence of anti-CD3 (1 μg ml^–1^; 145-2C11, eBioscience) and anti-CD28 (0.5 μg ml^–1^; 37.51, eBioscience) in RPMI medium supplemented with 10% FBS. Culture medium was completed with 10 ng ml^–1^ IL-12 (419-ML-010, R&D Systems) and 20 μg ml^–1^ anti-IL-4 (BE0045, BioXcell). Cells were cultured at 5% CO_2_ and 37 °C for 3 days. On average, 70% of the cells expressed IFNγ by 3 days of polarization culture.

### Immunofluorescence staining

Samples collected from TGFβR-WT, TGFβR-KO and TGFβR-KO; T-BET-KO mice were fixed in 4% PFA overnight before paraffin inclusion. Four-micron-thick slides were washed twice with PBS, permeabilized with 0.2% Triton X-100 (X100, Sigma-Aldrich) and blocked in PBS with 2% bovine serum albumin (A7906, Sigma-Aldrich) for 1 h at room temperature. Slides were then incubated with primary antibodies overnight at 4 °C. For mouse tissue staining, anti-GFP (A-11122, Invitrogen), CD4-PE (GK1.5 eBioscience) and anti-γH2AX (9718S, Ozyme) were used. Secondary antibodies (goat anti-rabbit AlexaFluor 488; A32731, Invitrogen) were incubated for 1 h at room temperature. For nucleus detection, DAPI (D3571, Invitrogen) was used. Images were then acquired using a Confocal Zeiss 980 microscope and analyzed with ImageJ software.

### scRNA-seq

After tissue dissection and dissociation, fluorescence-activated cell sorting-purified suspended YFP^+^CD4^+^ T cells were immediately partitioned into nanoliter-scale Gel Bead-In-Emulsions (GEMs) with a Chromium Single Cell Controller (10x Genomics) at the Cancer Research Center of Lyon (CRCL) Single Cell Platform. Cell encapsulation and barcoding were followed by the standard scRNA-seq protocol, including reverse transcription, amplification and indexing (10x Genomics). Sequencing was performed using a NovaSeq Illumina device (Illumina). Illumina bcl files were base called, demultiplexed and aligned to the mouse mm10 genome using CellRanger software (10x Genomics). The output of CellRanger was used to run the Python package velocyto and produce loom files for each sample with RNA velocity estimations^55^. Loom files from two independent sequencing runs were imported into R, and single-cell data were analyzed with the ‘Seurat’ package^56^. The first batch included barcoded TGFβR-KO and TGFβR-WT T_H_17 cells, whereas the second batch was composed of TGFβR-CA and TGFβR-WT cells. Integration of these two datasets followed the ‘Fast integration using reciprocal PCA (RPCA)’ protocol. After filtering for library size (between 1,000 and 5,000 features per cell) and mitochondrial gene expression (less than 10%), preprocessing was performed using Seurat functions for counts normalization (SCTransform using the ‘glmGamPoi’ method). RPCA integration using 3,000 integration features was followed by dimension reduction with principal component analysis (RunPCA with default parameters), construction of a shared nearest neighbor graph (FindNeighbors using ten dimensions of reduction as input based on an elbow plot of variance captured by each principal component), clustering (FindClusters with a resolution of 0.5) and visualization with the UMAP dimensional reduction technique. Initial marker identification was used to identify and remove γδT cells (based on the expression of ‘*Tcrg*’ and ‘*Trg*’ genes) and contaminating epithelial cells. In total, 5,080 cells remained for downstream analysis after this final filtering step (TGFβR-CA = 1,098, TGFβR-WT = 1,007 and TGFβR-KO = 2,975). Markers of each cluster were identified using the Wilcox test option of the FindAllMarkers function, with a logarithmic fold change threshold of 0.25 and adjusted *P* value under 5%. Known markers were used to identify and relabel the resulting clusters, as described in the text. In addition, the COMET package^57^ was used to identify and validate surface markers distinct from each cluster. A G2/M cell cycle phase score was calculated using an established list of cell cycle markers^25^. Regulatory network analysis was performed to identify core TFs orchestrating transcriptional programs within each cluster using the single-cell regulatory network inference and clustering (SCENIC) R package^58^ (https://github.com/aertslab/SCENIC). SCENIC infers coexpression modules between TF and candidate target genes (that is, regulons) using machine learning. Once all regulons are constructed, SCENIC scores its corresponding activity for each individual cell. We used the dynverse R package to compare across pseudotime algorithms and choose the one best suited for our integrated dataset^59^. Final trajectory (pseudotime) analyses were performed using the slingshot R package^60^ To this end, clustering information was extracted from Seurat objects for each individual condition and passed directly to slingshot’s main function. The same ‘granularity’ parameters (that is, Omega = 3) were used for all conditions to ensure comparability. Once pseudotime trajectories were identified, a general additive model was fitted to identify genes whose expression was significantly associated with each trajectory. CellRank was used for directional trajectory analyses^61^. CellRank combines trajectory inference (pseudotime) with directional information from RNA velocity, automatically predicting initial, intermediate and terminal cell populations.

### scATAC-seq

CD4^+^YFP^+^ T cells were purified from the small intestine, including PP, from TGFβR-WT and TGFβR-KO mice. Cell nuclei were independently prepared and frozen following the recommended conditions for scATAC-seq using the 10x Genomics protocol for library preparation (outsourced with ActiveMotif). Thirty-four-base pair paired-end sequencing reads were generated by Illumina Sequencing using a NextSeq 500. Reads were mapped to the mm10 genome, and peaks were called using CellRanger ATAC software with default parameters (mkfastq and count functions). The Signac package (https://satijalab.org/signac/news/index.html) was used in combination with Seurat for all downstream analyses after Tn5 mapping. Briefly, the pipeline includes the creation of a chromatin assay to which nucleosome signal, transcription start site enrichment and fragment data are consecutively added. Next, latent semantic indexing is performed using the ‘RunTFIDF’ and ‘RunSVD’ functions, followed by clustering and UMAP visualization. Gene activity is inferred using the ‘GeneActivity’ function. Merging of TGFβR-WT and TGFβR-KO samples was performed after selecting common good-quality peaks, resulting in 90,255 features across 3,860 cells (after filtering out ten *Tcrg*/*Trg*-expressing γδT cells). The combined dataset was further annotated using label transfer from the scRNA-seq data following Seurat’s recommended protocol. Differential accessibility was performed using the ‘FindAllMarkers’ function on peak assay data. Motif analyses were performed with Signac’s ‘AddMotifs’ and ‘RunChromVAR’ functions.

### Single-cell TCR sequencing and repertoire analysis

As with scRNA-seq, fluorescence-activated cell sorting-purified suspended YFP^+^CD4^+^ T cells from the small intestine were partitioned into nanoliter-scale GEMs with the Chromium Single Cell Controller (10x Genomics), and gene expression and TCRαβ libraries were prepared using a Chromium Single Cell 5′ Library & Gel Bead kit (10x Genomics), as per the manufacturer’s instructions. Sequencing was performed using a NovaSeq Illumina device (Illumina). Illumina bcl files were base called, demultiplexed and aligned to the mouse mm10 genome using CellRanger version 7.1.0 (10x Genomics) in ‘multi’ mode (gene expression + vdj). Demultiplexed data were loaded into R and analyzed with the ‘scRepertoire’ package version 1.12.0 (https://f1000research.com/articles/9-47/v2). scRepertoire was used to assign clonotypes based on TCR chains, quantify and study clonotype dynamics and integrate with gene expression data in combination with the Seurat package. Clonotypes were called by a combination of *CDR3* nucleotide sequence and VDJC gene sequence (CTstrict). Shared clonotypes were defined as clonotypes coming from different cell types and containing the same CDR3 nucleotide and VDJC gene sequences.

### ChIP

Sorted YFP^+^CD4^+^ T cells were processed with a CUT&RUN assay kit (Cell Signaling), following the manufacturer’s protocol. Anti-KLF6 (sc-365633, Santa Cruz) was used at 2 μg per sample for ChIP. DNA was then purified using DNA spin columns (14209S, Cell Signaling), and 10 ng of DNA per reaction was used for quantitative real-time PCR using LightCycler 480 SYBR Green Master (4707516001, Roche). The following primer sequences were used: *Tbx21* CNS0: forward 5′-CTGGAAAATCAGGCTCACGC-3′ and reverse 5′-ACTTTTCCCAGCTTCGAGGA-3′; *Tbx21* intragenic region: forward 5′-CACATGAAGTAGGAAGCGCC-3′ and reverse 5′-GGGGAGAGCTGGTGTTAAGT-3′.

### Chromatin accessibility

*Tbx21* DNA accessibility was tested on sorted YFP^+^CD4^+^ T cells using an EpiQuik Chromatin Accessibility Assay kit (EpiGenTek) following the manufacturer’s protocol. Isolated chromatin from 2 × 10^4^ cells was then amplified by quantitative PCR. Quantitative PCR was performed using LightCycler 480 SYBR and the same primer sequences depicted above. Fold enrichment was then calculated by the formula fold enrichment = $2^{({\rm{Nnse}}{\rm{C}}_{t}-{\rm{no}}\,{\rm{Nnse}}{\rm{C}}_{t})}$ × 100, where Nnse*C*_t_ refers to nuclease-treated sample cycling threshold (*C*_t_), and no Nnse*C*_t_ refers to the control nontreated sample.

### CRISPR–Cas9 deletion

gRNAs were purchased from Sigma, and mixes with equimolar concentrations of gRNA and trRNA (150 pmol) were incubated for 5 min at 95 °C. The duplexes were incubated for 15 min at 37 °C with TrueCut Cas9 Nuclease V2 (50 pmol; Thermo Fisher) to form ribonucleoprotein complexes. Purified CD4^+^ T cells from the small intestine were resuspended with the ribonucleoprotein complexes and the Electroporation Enhancer in P3 Primary Cell buffer (Lonza) just before electroporation with 4D Nucleofactor (Lonza, program DN100). Electroporated cells, stained with Atto550 fluorescent molecule, were recovered in RPMI medium supplemented with 10% FBS for 30 min before being injected i.v. in C57BL/6 mice. The following gRNA sequences were used: *Klf6* 5′-CACGAAACGGGCTACTTCT-3′ and control 5′-CGCGATAGCGGCGAATATATT-3′.

### Quantitative PCR with reverse transcription

mRNAs were isolated using an RNeasy mini kit (Qiagen) and reverse transcribed with an iScript cDNA synthesis kit (Bio-Rad). Quantitative PCR with reverse transcription was performed using LightCycler 480 SYBR Green Master and different sets of primers on a LightCycler 480 Real-Time PCR System (Roche). Sample gene expression was normalized to the levels of *Gadph* and analyzed according to the ΔΔ*C*_t_ method. The following primer sequences were used: *Tgfb1* forward 5′-CCCGAAGCGGACTACTATGC-3′ and reverse 5′-ATAGATGGCGTTGTTGCGGT-3′; *Gadph* forward 5′-GCATGGCCTTCCGTGTTC-3′ and reverse 5′-TGTCATCATACTTGGCAGGTTTCT-3′.

### Enzyme-linked immunosorbent assay (ELISA)

Tissue sections 1 cm in length of the different intestinal segments were placed into RPMI medium (Gibco) containing 2% FBS (Gibco) and incubated for 24 h at 37 °C with 5% CO_2_. Supernatants were collected and stored before analysis at −80 °C. IL-12p70, IL-23p19 and IL-22 were measured by ELISA according to the manufacturer’s instructions (Invitrogen, BMS616 (IL-12p70); Invitrogen, BMS6017 (IL-23p19); Biolegend, 436304 (IL-22)). Optical density was read using a TECAN Infinite m1000 microplate reader at 450 nm.

### Mapping human and mouse KLF6 binding sites on *TBX21*

Human and mouse (*TBX21* and *Tbx21*, respectively) loci were screened for KLF6 binding sites, and their respective chromosomal locations (mm10 and hg38 assemblies, respectively) were used to obtain *Tbx21* DNA sequences extended by 15,000 bp up- and downstream (GenomicFeatures package). Sequences were then scanned for TF motifs from the MotifDb annotated collection using the Biostrings package function ‘match PWM’.

### *KLF6* expression in donor T_H_17 cells

CD45^+^ cells from (1) cerebrospinal fluid from individuals with multiple sclerosis and healthy donors, (2) colonic biopsies from individuals with ulcerative colitis and healthy donors or (3) ileal biopsies from individuals with Crohn’s disease in inflamed and not inflamed regions were analyzed by scRNA-seq^47–49^. For multiple sclerosis, we used the data from all six individuals and six healthy donors^48^. For ulcerative colitis, we used data from three individuals and three healthy donors^49^. For Crohn’s disease, we used data from nine individuals containing inflamed and noninflamed regions of the ileum of a same patient^47^. For all samples, we first identified the CD4^+^ T cell population. Gene expression signatures for T_H_17 cells and T_reg_ cells were then applied to determine the T_H_17 and T_reg_ cell clusters (Supplementary Table 5) using R software with AddModuleScore UCell packages. *KLF6* expression levels in T_H_17 cells were determined and normalized to those in T_reg_ cells from the same donor.

### Statistics

All statistical analyses were performed using Prism v9.4.1 (GraphPad) except for permutational multivariate analysis of variance, for which Qiime2 2020.8 was used. Statistical relevance was evaluated using an unpaired *t*-test or Mann–Whitney test when appropriate. Data distribution was assumed to be normal (unless stated otherwise), but this was not formally tested. Mice were chosen randomly for the experiments. Differences were considered significant when *P* values were <0.05. No statistical methods were used to predetermine sample sizes, but our sample sizes are similar to those reported in previous publications^62^. No data points nor animals were excluded from the analysis. Experimental conditions were organized randomly. Data collection and analysis were performed blind to the conditions of the experiments for histology scores.

### Reporting summary

Further information on research design is available in the Nature Portfolio Reporting Summary linked to this article.

## Online content

Any methods, additional references, Nature Portfolio reporting summaries, source data, extended data, supplementary information, acknowledgements, peer review information; details of author contributions and competing interests; and statements of data and code availability are available at 10.1038/s41590-024-01909-7.

## Supplementary information

Supplementary Information Supplementary Figs. 1–10.

Reporting Summary

Supplementary Data Statistical source data for the supplementary figures.

## Source data

Source Data Fig. 1 Statistical source data.

Source Data Fig. 2 Statistical source data.

Source Data Fig. 3 Statistical source data.

Source Data Fig. 4 Statistical source data.

Source Data Fig. 5 Statistical source data.

Source Data Fig. 6 Statistical source data.

Source Data Extended Data Fig. 1 Statistical source data.

Source Data Extended Data Fig. 3 Statistical source data.

Source Data Extended Data Fig. 5 Statistical source data.

Source Data Extended Data Fig. 6 Statistical source data.

Source Data Extended Data Fig. 7 Statistical source data.

Source Data Extended Data Fig. 8 Statistical source data.

## Data Availability

scRNA-seq and scATAC-seq data have been deposited in the Gene Expression Omnibus under the accession code GSE235513. 16S rRNA sequencing data have been deposited in NCI under the accession code PRJNA1125870. Source data are provided with this paper.

## References

[1] Grivennikov, S. I., Greten, F. R. & Karin, M. Immunity, inflammation, and cancer. *Cell* **140**, 883–899 (2010). 20303878 10.1016/j.cell.2010.01.025 PMC2866629

[2] Grivennikov, S. I. Inflammation and colorectal cancer: colitis-associated neoplasia. *Semin. Immunopathol.* **35**, 229–244 (2013). 23161445 10.1007/s00281-012-0352-6 PMC3568220

[3] Korn, T., Bettelli, E., Oukka, M. & Kuchroo, V. K. IL-17 and T_H_17 cells. *Annu. Rev. Immunol.* **27**, 485–517 (2009). 19132915 10.1146/annurev.immunol.021908.132710

[4] Opejin, A. et al. A two-step process of effector programming governs CD4^+^ T cell fate determination induced by antigenic activation in the steady state. *Cell Rep.* **33**, 108424 (2020). 33238127 10.1016/j.celrep.2020.108424 PMC7714042

[5] Bettelli, E. et al. Reciprocal developmental pathways for the generation of pathogenic effector T_H_17 and regulatory T cells. *Nature* **441**, 235–238 (2006). 16648838 10.1038/nature04753

[6] Mangan, P. R. et al. Transforming growth factor-β induces development of the T_H_17 lineage. *Nature* **441**, 231–234 (2006). 16648837 10.1038/nature04754

[7] Biancheri, P. et al. The role of transforming growth factor (TGF)-β in modulating the immune response and fibrogenesis in the gut. *Cytokine Growth Factor Rev.* **25**, 45–55 (2014). 24332927 10.1016/j.cytogfr.2013.11.001

[8] Tanaka, S. et al. Trim33 mediates the proinflammatory function of T_H_17 cells. *J. Exp. Med.* **215**, 1853–1868 (2018). 29930104 10.1084/jem.20170779 PMC6028517

[9] Zhang, S. et al. Reversing SKI–SMAD4-mediated suppression is essential for T_H_17 cell differentiation. *Nature* **551**, 105–109 (2017). 29072299 10.1038/nature24283 PMC5743442

[10] Derynck, R. & Zhang, Y. E. Smad-dependent and Smad-independent pathways in TGF-β family signalling. *Nature* **425**, 577–584 (2003). 14534577 10.1038/nature02006

[11] Mills, K. H. G. IL-17 and IL-17-producing cells in protection versus pathology. *Nat. Rev. Immunol.* **23**, 38–54 (2023). 35790881 10.1038/s41577-022-00746-9 PMC9255545

[12] Schnell, A., Littman, D. R. & Kuchroo, V. K. T_H_17 cell heterogeneity and its role in tissue inflammation. *Nat. Immunol.* **24**, 19–29 (2023). 36596896 10.1038/s41590-022-01387-9 PMC10795475

[13] Heinemann, C. et al. IL-27 and IL-12 oppose pro-inflammatory IL-23 in CD4^+^ T cells by inducing Blimp1. *Nat. Commun.* **5**, 3770 (2014). 24796719 10.1038/ncomms4770

[14] Hirota, K. et al. Fate mapping of IL-17-producing T cells in inflammatory responses. *Nat. Immunol.* **12**, 255–263 (2011). 21278737 10.1038/ni.1993 PMC3040235

[15] Levéen, P. et al. TGF-β type II receptor-deficient thymocytes develop normally but demonstrate increased CD8^+^ proliferation in vivo. *Blood* **106**, 4234–4240 (2005). 16131565 10.1182/blood-2005-05-1871

[16] Bartholin, L. et al. Generation of mice with conditionally activated transforming growth factor β signaling through the TβRI/ALK5 receptor. *Genesis* **46**, 724–731 (2008). 18821589 10.1002/dvg.20425

[17] Turpin, A., El Amrani, M. & Zaanan, A. Localized small bowel adenocarcinoma management: evidence summary. *Cancers* **14**, 2892 (2022). 35740558 10.3390/cancers14122892 PMC9220873

[18] Doisne, J.-M. et al. Skin and peripheral lymph node invariant NKT cells are mainly retinoic acid receptor-related orphan receptor γt^+^ and respond preferentially under inflammatory conditions. *J. Immunol.* **183**, 2142–2149 (2009). 19587013 10.4049/jimmunol.0901059

[19] Legoux, F. et al. Molecular mechanisms of lineage decisions in metabolite-specific T cells. *Nat. Immunol.* **20**, 1244–1255 (2019). 31431722 10.1038/s41590-019-0465-3

[20] Lowndes, N. F. & Toh, G. W.-L. DNA repair: the importance of phosphorylating histone H2AX. *Curr. Biol.* **15**, R99–R102 (2005). 15694301 10.1016/j.cub.2005.01.029

[21] Oh, H. & Ghosh, S. NF-κB: roles and regulation in different CD4^+^ T-cell subsets. *Immunol. Rev.* **252**, 41–51 (2013). 23405894 10.1111/imr.12033 PMC3576882

[22] Bourque, J., Kousnetsov, R. & Hawiger, D. Roles of Hopx in the differentiation and functions of immune cells. *Eur. J. Cell Biol.* **101**, 151242 (2022). 35636259 10.1016/j.ejcb.2022.151242

[23] Hirota, K. et al. Plasticity of T_H_17 cells in Peyer’s patches is responsible for the induction of T cell-dependent IgA responses. *Nat. Immunol.* **14**, 372–379 (2013). 23475182 10.1038/ni.2552 PMC3672955

[24] Chang, Y. et al. TGF-β specifies T_FH_ versus T_H_17 cell fates in murine CD4^+^ T cells through c-Maf. *Sci. Immunol.* **9**, eadd4818 (2024). 38427718 10.1126/sciimmunol.add4818

[25] Tirosh, I. et al. Dissecting the multicellular ecosystem of metastatic melanoma by single-cell RNA-seq. *Science* **352**, 189–196 (2016). 27124452 10.1126/science.aad0501 PMC4944528

[26] Kiner, E. et al. Gut CD4^+^ T cell phenotypes are a continuum molded by microbes, not by T_H_ archetypes. *Nat. Immunol.* **22**, 216–228 (2021). 33462454 10.1038/s41590-020-00836-7 PMC7839314

[27] Schnell, A. et al. Stem-like intestinal T_H_17 cells give rise to pathogenic effector T cells during autoimmunity. *Cell* **184**, 6281–6298 (2021). 34875227 10.1016/j.cell.2021.11.018 PMC8900676

[28] Bettelli, E. & Kuchroo, V. K. IL-12- and IL-23-induced T helper cell subsets: birds of the same feather flock together. *J. Exp. Med.* **201**, 169–171 (2005). 15657286 10.1084/jem.20042279 PMC2212800

[29] Bai, J. & Xi, Q. Crosstalk between TGF-β signaling and epigenome. *Acta Biochim. Biophys. Sin.* **50**, 60–67 (2018). 29190318 10.1093/abbs/gmx122

[30] Fang, D. et al. Differential regulation of transcription factor T-BET induction during NK cell development and T helper-1 cell differentiation. *Immunity* **55**, 639–655 (2022). 35381213 10.1016/j.immuni.2022.03.005 PMC9059963

[31] Marie, J. C., Liggitt, D. & Rudensky, A. Y. Cellular mechanisms of fatal early-onset autoimmunity in mice with the T cell-specific targeting of transforming growth factor-β receptor. *Immunity* **25**, 441–454 (2006). 16973387 10.1016/j.immuni.2006.07.012

[32] Gutcher, I. et al. Autocrine transforming growth factor-β1 promotes in vivo T_H_17 cell differentiation. *Immunity* **34**, 396–408 (2011). 21435587 10.1016/j.immuni.2011.03.005 PMC3690311

[33] Maloy, K. J. & Powrie, F. Intestinal homeostasis and its breakdown in inflammatory bowel disease. *Nature* **474**, 298–306 (2011). 21677746 10.1038/nature10208

[34] Zhang, S. The role of transforming growth factor β in T helper 17 differentiation. *Immunology* **155**, 24–35 (2018). 29682722 10.1111/imm.12938 PMC6099164

[35] Park, I.-K., Letterio, J. J. & Gorham, J. D. TGF-β1 inhibition of IFN-γ-induced signaling and T_H_1 gene expression in CD4^+^ T cells is Smad3 independent but MAP kinase dependent. *Mol. Immunol.* **44**, 3283–3290 (2007). 17403540 10.1016/j.molimm.2007.02.024 PMC2134969

[36] Lee, J.-Y. et al. Serum amyloid A proteins induce pathogenic T_H_17 cells and promote inflammatory disease. *Cell* **180**, 79–91 (2020). 31866067 10.1016/j.cell.2019.11.026 PMC7039443

[37] Lee, J. S. et al. Interleukin-23-independent IL-17 production regulates intestinal epithelial permeability. *Immunity* **43**, 727–738 (2015). 26431948 10.1016/j.immuni.2015.09.003 PMC6044435

[38] Megaraj, V. et al. Role of hepatic and intestinal P450 enzymes in the metabolic activation of the colon carcinogen azoxymethane in mice. *Chem. Res. Toxicol.* **27**, 656–662 (2014). 24552495 10.1021/tx4004769 PMC4002058

[39] Grivennikov, S. I. et al. Adenoma-linked barrier defects and microbial products drive IL-23/IL-17-mediated tumour growth. *Nature* **491**, 254–258 (2012). 23034650 10.1038/nature11465 PMC3601659

[40] Wang, K. et al. Interleukin-17 receptor A signaling in transformed enterocytes promotes early colorectal tumorigenesis. *Immunity* **41**, 1052–1063 (2014). 25526314 10.1016/j.immuni.2014.11.009 PMC4272447

[41] Hubackova, S. et al. IFNγ induces oxidative stress, DNA damage and tumor cell senescence via TGFβ/SMAD signaling-dependent induction of Nox4 and suppression of ANT2. *Oncogene* **35**, 1236–1249 (2016). 25982278 10.1038/onc.2015.162

[42] Gronke, K. et al. Interleukin-22 protects intestinal stem cells against genotoxic stress. *Nature* **566**, 249–253 (2019). 30700914 10.1038/s41586-019-0899-7 PMC6420091

[43] Cao, Z., Sun, X., Icli, B., Wara, A. K. & Feinberg, M. W. Role of Kruppel-like factors in leukocyte development, function, and disease. *Blood* **116**, 4404–4414 (2010). 20616217 10.1182/blood-2010-05-285353 PMC2996110

[44] Crawford, A. et al. Molecular and transcriptional basis of CD4^+^ T cell dysfunction during chronic infection. *Immunity* **40**, 289–302 (2014). 24530057 10.1016/j.immuni.2014.01.005 PMC3990591

[45] Ciofani, M. et al. A validated regulatory network for T_H_17 cell specification. *Cell* **151**, 289–303 (2012). 23021777 10.1016/j.cell.2012.09.016 PMC3503487

[46] Derynck, R., Turley, S. J. & Akhurst, R. J. TGFβ biology in cancer progression and immunotherapy. *Nat. Rev. Clin. Oncol.* **18**, 9–34 (2021). 32710082 10.1038/s41571-020-0403-1 PMC9721352

[47] Martin, J. C. et al. Single-cell analysis of Crohn’s disease lesions identifies a pathogenic cellular module associated with resistance to anti-TNF therapy. *Cell* **178**, 1493–1508 (2019). 31474370 10.1016/j.cell.2019.08.008 PMC7060942

[48] Schafflick, D. et al. Integrated single cell analysis of blood and cerebrospinal fluid leukocytes in multiple sclerosis. *Nat. Commun.* **11**, 247 (2020). 31937773 10.1038/s41467-019-14118-w PMC6959356

[49] Smillie, C. S. et al. Intra- and inter-cellular rewiring of the human colon during ulcerative colitis. *Cell* **178**, 714–730 (2019). 31348891 10.1016/j.cell.2019.06.029 PMC6662628

[50] el Marjou, F. et al. Tissue-specific and inducible Cre-mediated recombination in the gut epithelium. *Genesis* **39**, 186–193 (2004). 15282745 10.1002/gene.20042

[51] Srinivas, S. et al. Cre reporter strains produced by targeted insertion of EYFP and ECFP into the *ROSA26* locus. *BMC Dev. Biol.* **1**, 4 (2001). 11299042 10.1186/1471-213X-1-4 PMC31338

[52] Doisne, J.-M. et al. iNKT cell development is orchestrated by different branches of TGF-β signaling. *J. Exp. Med.* **206**, 1365–1378 (2009). 19451264 10.1084/jem.20090127 PMC2715067

[53] Azhar, M. et al. Generation of mice with a conditional allele for transforming growth factor β 1 gene. *Genesis* **47**, 423–431 (2009). 19415629 10.1002/dvg.20516 PMC2766615

[54] Rocha, C. et al. Tubulin glycylases are required for primary cilia, control of cell proliferation and tumor development in colon. *EMBO J.* **33**, 2247–2260 (2014). 25180231 10.15252/embj.201488466 PMC4282510

[55] La Manno, G. et al. RNA velocity of single cells. *Nature* **560**, 494–498 (2018). 30089906 10.1038/s41586-018-0414-6 PMC6130801

[56] Stuart, T. et al. Comprehensive integration of single-cell data. *Cell* **177**, 1888–1902.e21 (2019). 31178118 10.1016/j.cell.2019.05.031 PMC6687398

[57] Delaney, C. et al. Combinatorial prediction of marker panels from single-cell transcriptomic data. *Mol. Syst. Biol.* **15**, e9005 (2019). 31657111 10.15252/msb.20199005 PMC6811728

[58] Aibar, S. et al. SCENIC: single-cell regulatory network inference and clustering. *Nat, Methods* **14**, 1083–1086 (2017). 28991892 10.1038/nmeth.4463 PMC5937676

[59] Saelens, W., Cannoodt, R., Todorov, H. & Saeys, Y. A comparison of single-cell trajectory inference methods. *Nat. Biotechnol.* **37**, 547–554 (2019). 30936559 10.1038/s41587-019-0071-9

[60] Street, K. et al. Slingshot: cell lineage and pseudotime inference for single-cell transcriptomics. *BMC Genomics* **19**, 477 (2018). 29914354 10.1186/s12864-018-4772-0 PMC6007078

[61] Lange, M. et al. CellRank for directed single-cell fate mapping. *Nat. Methods* **19**, 159–170 (2022). 35027767 10.1038/s41592-021-01346-6 PMC8828480

[62] Reis, S. et al. TCR-Vγδ usage distinguishes protumor from antitumor intestinal γδ T cell subsets. *Science* **377**, 276–284 (2022). 35857588 10.1126/science.abj8695 PMC9326786

