# Supplementary material for: An intestinal TH17 cell-derived subset can initiate cancer
Source: Nat Immunol. 2024 Jul 26;25(9):1637–49. doi: 10.1038/s41590-024-01909-7 (PMC11362008; doi:10.1038/s41590-024-01909-7)
Supplement: Supplementary file 1 — Supplementary Figs. 1–10. [file 41590_2024_1909_MOESM1_ESM.pdf]

nature immunology

Supplementary information

https://doi.org/10.1038/s41590-024-01909-7

An intestinal T_H17 cell-derived subset can initiate cancer

In the format provided by the authors and unedited

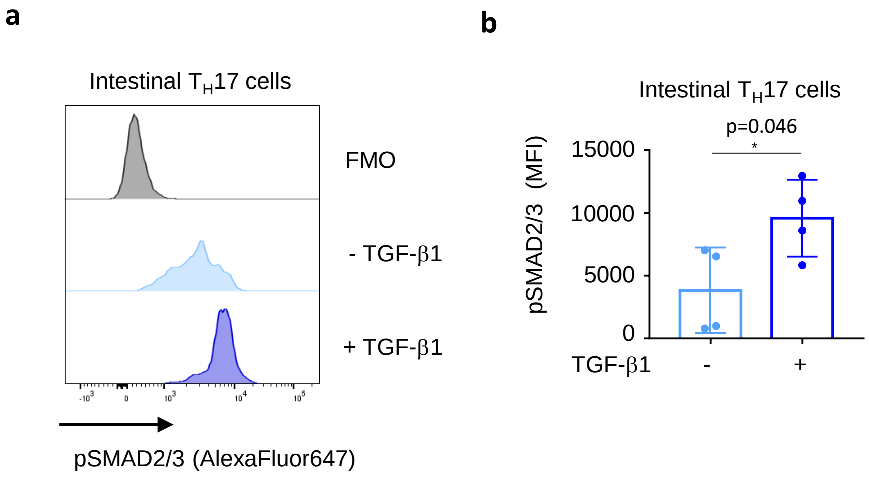

Figure S1: Differentiated TH17 cells respond to TGF-β1

YFP^{pos} CD4^{pos} TCRβ^{pos} cells were purified from the SILP of *Il17a-Cre; Rosa stop*$^{fl/fl}$ *Yfp* reporter mice, and stimulated or not with TGF-β1. The phosphorylation of SMAD2/3 (p-SMAD2/3) was analyzed by flow cytometry. (**A**) Representative histograms are shown. The Fluorescence Minus One (FMO) was used as control. (**B**) Quantification of the mean fluorescence intensity (MFI) of p-SMAD2/3 staining (mean ± SD) is presented. Data are representative of 4 animals of two independent experiments. The statistical significance was determined by two-tailed t test.

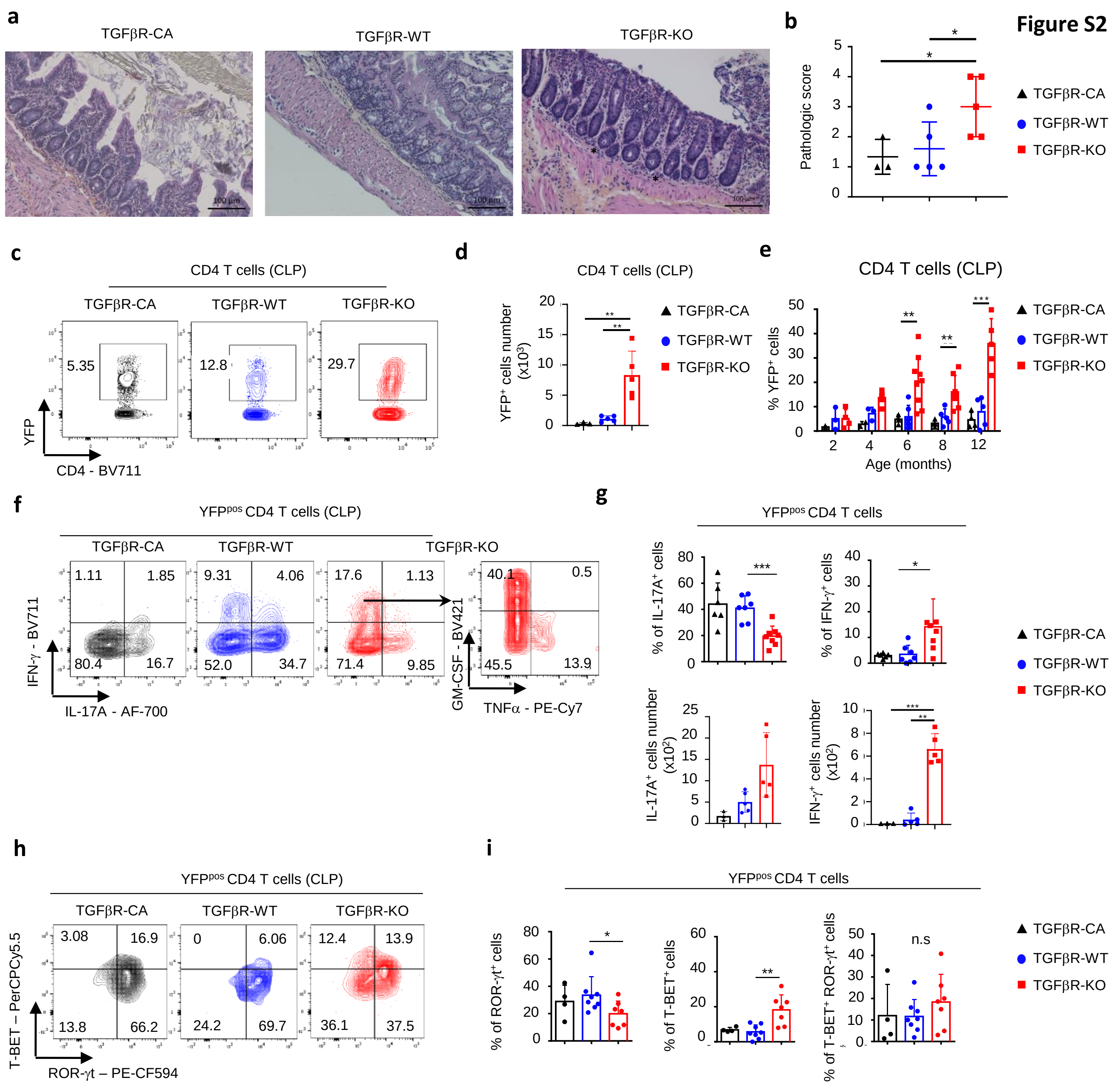

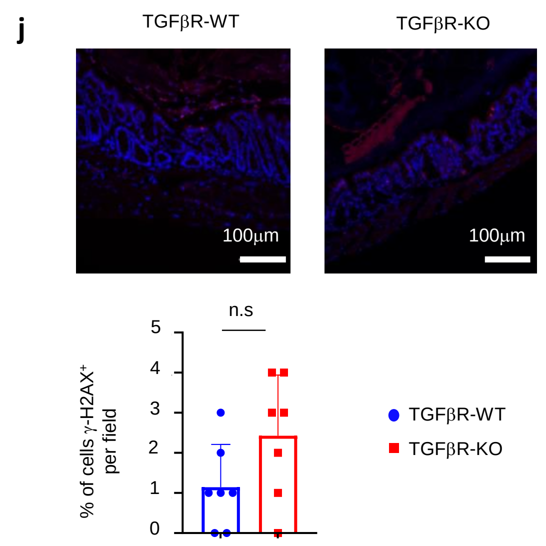

Figure S2: Mild colonic CIAC in TGFβR-KO mice

(A-B) colons from 10 month-old animals were isolated and analyzed by H&E staining. **(A)** Representative colon histology for the 3 groups of mice each dot represent one mouse. Micro-abscesses are indicated by asterisks. Scale bares are indicated on the pictures. **(B)** Representation of the pathologic scores calculated for the colon of the animals. Data are representative of 3-4 animals per group and of two independent experiments. (**C-E**) TGFβR-CA, TGFβR-KO and TGFβR-WT mice were crossed to *Rosa stop*$^{fl/fl}$ *Yfp* reporter mice. YFP^{pos} CD4 TCRβ cells isolated from CLP were analyzed by flow cytometry. **(C)** Representative counter plots of YFP expression among CD4 TCRβ cells in 8 month-old animals. **(D)** Absolut numbers per gramme of colon (**E**) Percentages of YFP^{pos} cells among CD4 TCRβ cells at different age (2-12 months) are represented (mean ± SD). Data were obtained from 5 independent experiments. **(F-I)** Cytokine production and transcription factor expression were evaluated in CLP YFP^{pos} CD4^{+} T cells from 8 month of age animals. Representative counter plots (F, H) as well as quantification of the percentages and absolute numbers per gramme of colon are depicted (G, I) (mean ± SD) Data were obtained from 5 independent experiments. **J)** Representative immune staining of the colon for γ–H2AX (Red) and DAPI (Blue). Histograms illustrate the numbers of positive IECs per field (Mean ±SD). Data are representative of 3-8 animals per group in three independent experiments. Statistical analysis were performed using a two-tailed Student's t test n:s not significant.*P<0.05; **P<0.01; ***P<0.001. n.s not statistically different.

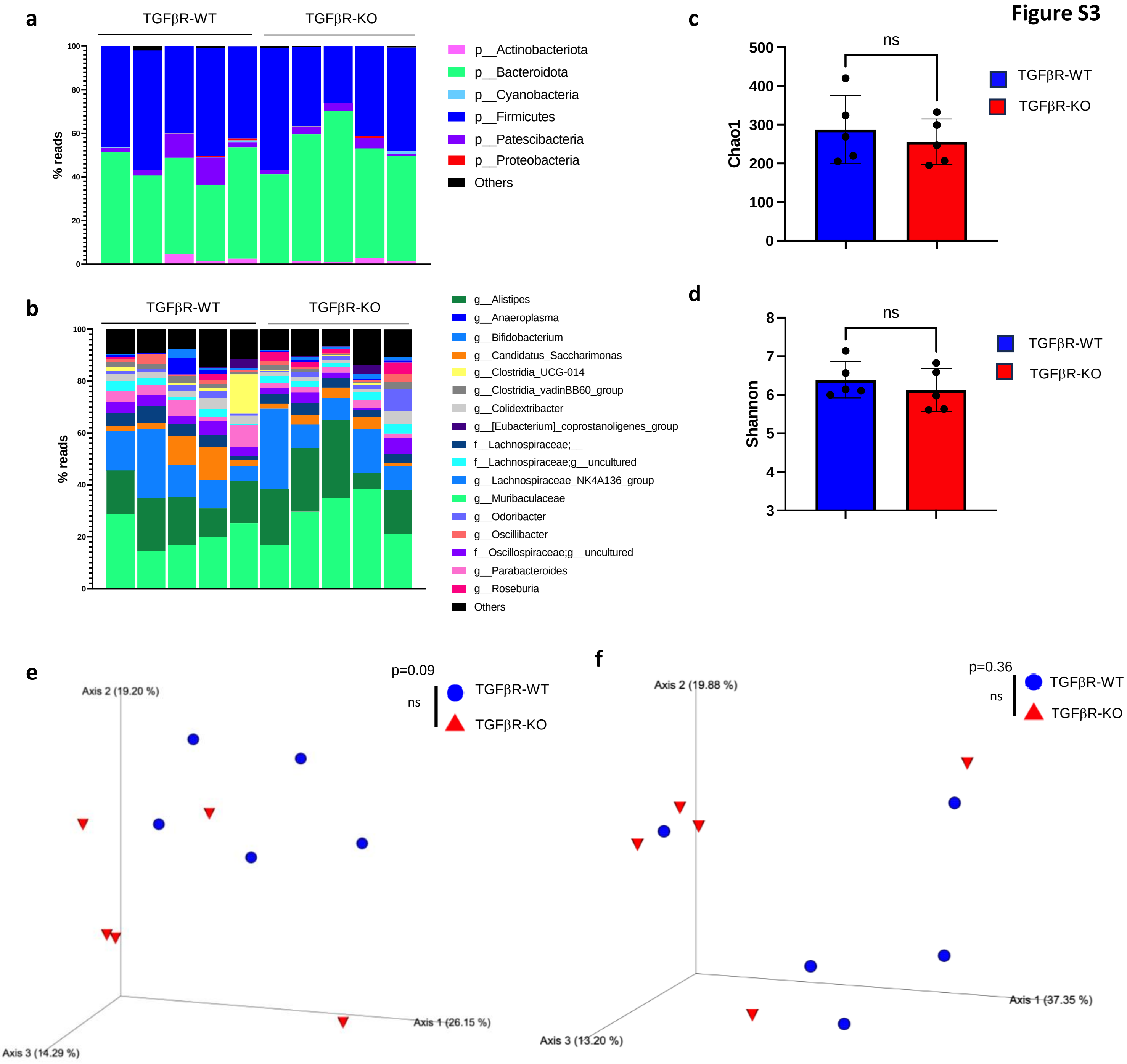

Figure S3: Absence of intestinal dysbiosis in TGFβR-KO mice

Bacteria purified from feces of TGFβR-WT and TGFβR-KO mice of different cages were analyzed. Global composition of the bacterial microbiota at the phylum (**A**) and genus (**B**) levels are illustrated Colored blocks indicate taxa relative abundance for each mouse. **C** and **D** Histograms illustrate Chao1 index (**C**) and Shannon index (**D**) describing the α-diversity of the bacterial microbiota (Means ±SD). Statistical difference was tested using the Mann-Whitney U test. Principal coordinate analysis of the Bray-Curtis distance (**E**) and the unweighted unifrac distance (**F**) between each sample. PCoA1 PCoA2 and PCoA3 represent the top three principal coordinates that captured most of the diversity. The fraction of diversity captured by the coordinate is given as a percentage. Groups were compared using Permanova method (999 permutations). ns non statistically different. Data were obtained from two independent experiments with 5 animals in total.

Figure S4

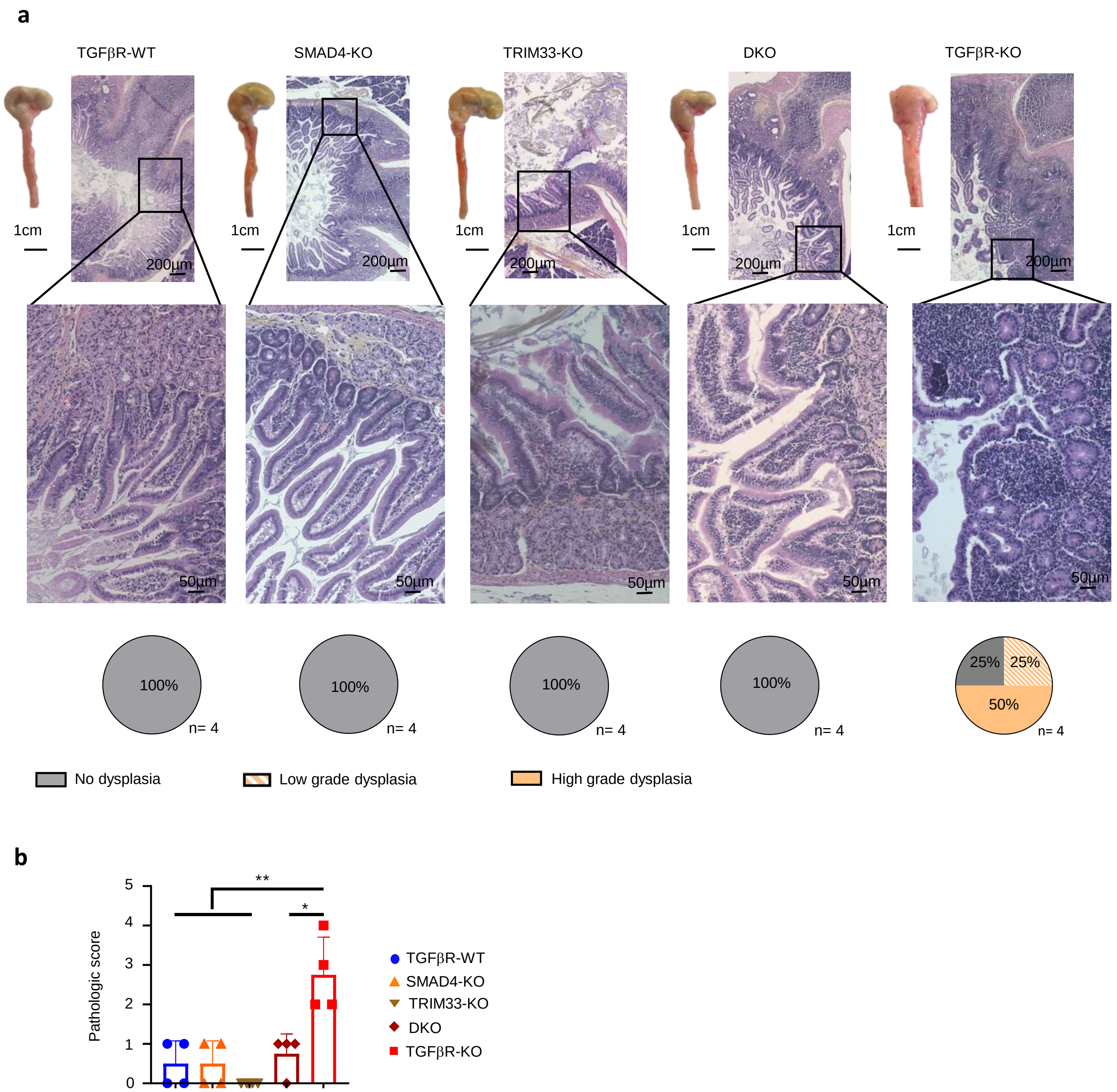

Figure S4: Tumorigenic potential of T_H17 cells is not controlled by SMAD4 and TRIM33-dependent pathways of the TGF-β signaling

Histological analysis was performed on the duodenum of 10 month-old *Il17a-Cre;Smad4*$^{fl/fl}$ (SMAD4-KO) mice, *Il17a-Cre;Trim33*$^{fl/fl}$ (TRIM33-KO) mice and *Il17a-Cre;Smad4*$^{fl/fl}$*;Trim33*$^{fl/fl}$ (DKO) mice. **A,** Representative pictures of the duodenum and H&E staining histology are showed. Scale-bars are indicated on the pictures. Data are representative of 4 mice per group. **B** Graph illustrates the pathologic score of the duodenum (mean ± SD) Statistical analysis were performed using a two-tailed Student's t test.*P<0.05; **P<0.01; ***P<0.001. Data are representative of 3 experiments with 4 animals per group.

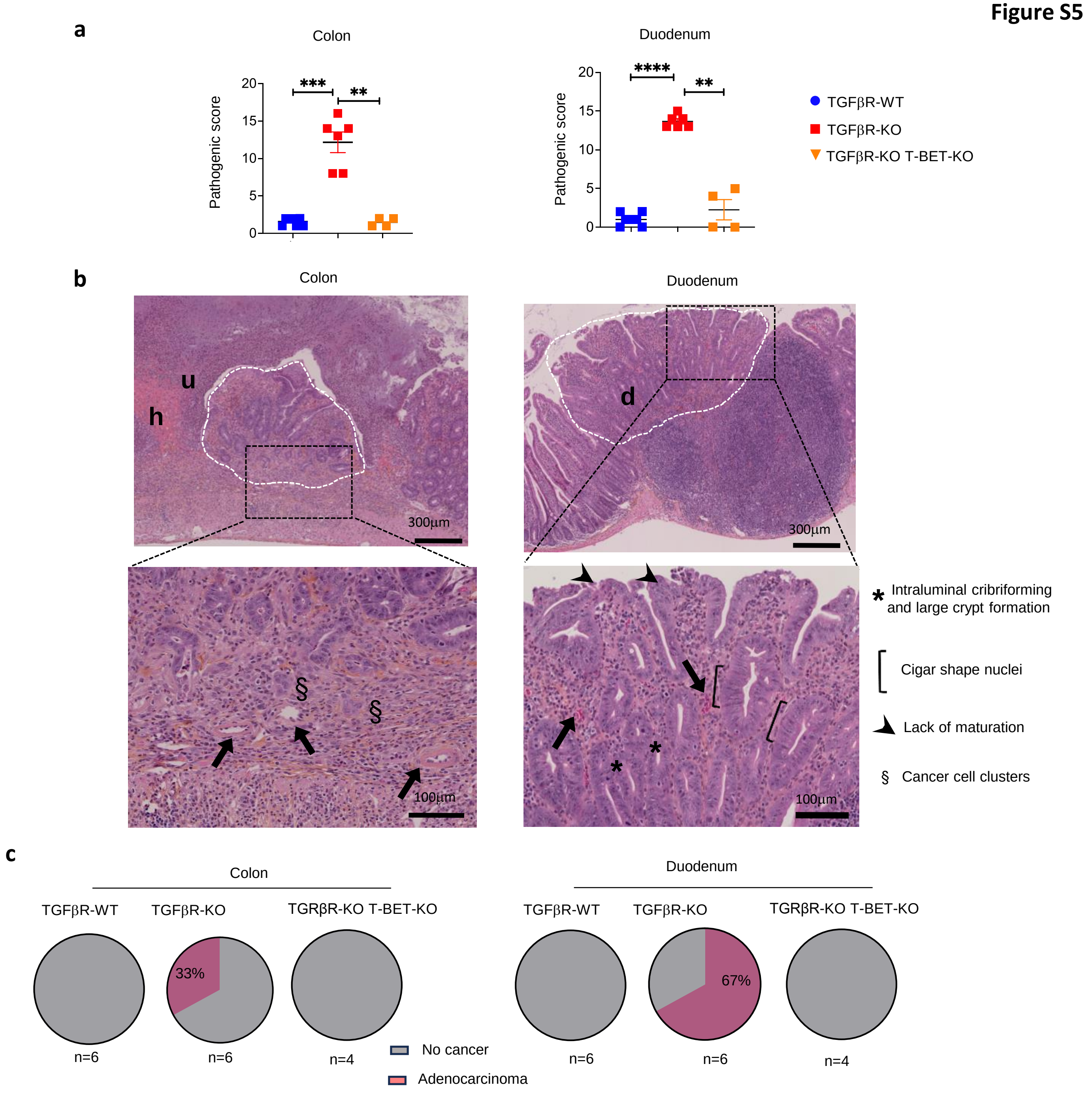

Figure S5: Tumorigenic TH17 cells promote genotoxic effects in both the duodenum and the colon

TGFβR-WT or TGFβR-KO or TGFβR-KO T-BET-KO mice were injected with AOM DSS. **A,** illustrates the pathologic score of these two segments 80 days after AOM treatment. (**B)** Representative images of H&E stainings performed on duodenum and colon sections from TGFβR-KO mice. Adenomas are delineated by dotted white line. Ulceration (u), hemorrhagic zones (h) and clusters of cancer cells at the edge of the muscularis mucosae as well as duodenal tubular adenoma, disrupting the mucosa (d) are indicated. Dysplasia was determined by the presence of intraluminal cribriforming and large crypt formation, enlarged, rounded and elongated cell nuclei (cigar shape), and lack of cell maturation at the epithelium surface. Blood capillaries are showed with arrows. **(C)** Representation of the percentage of animals with duodenal or colonic cancer based on histology analysis performed 80 days after AOM treatment. Scale-bars are indicated on the panels. Statistic relevance was evaluated by unpaired t test *P<0.05; **P<0.01; ****P<0.0001.

Figure S6

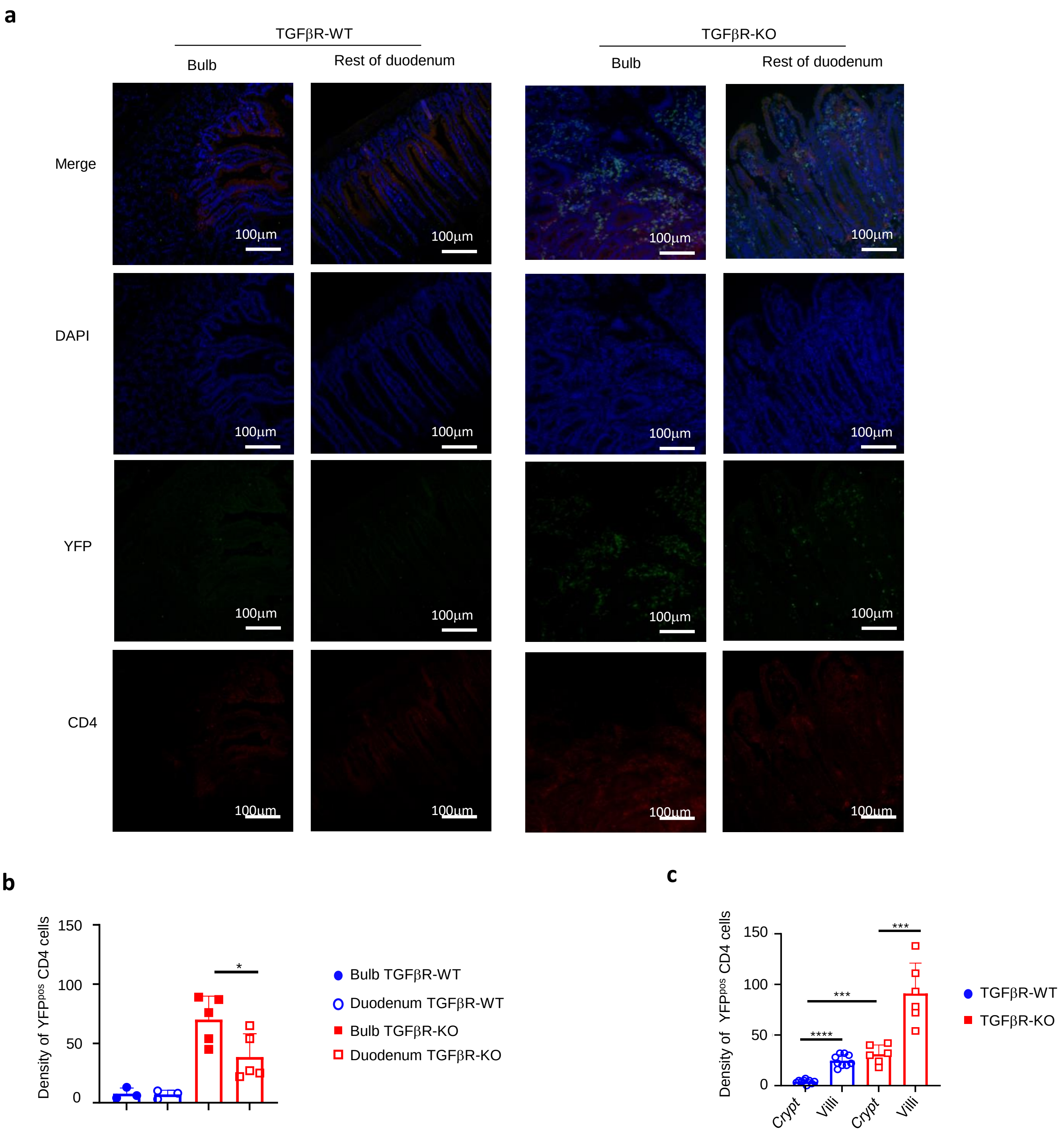

Figure S6: YFP CD4 T cell localization

Duodenum and its bulb of TGFβR-KO and TGFβR-WT mice crossed to *Rosa stop*$^{fl/fl}$ *Yfp* reporter mice were analyzed by immunostaining for YFP (Green) CD4 (Red) and DAPI (blue). (**A**) representative stainings (**B**) Graphs illustrates the density of YFP^{pos} CD4 cells counted by field in the bulb and the rest of the duodenum (mean $\pm$SD). (**C**) Graph illustrates the numbers of YFP^{pos} CD4 cells within the villi and at the bottom of the villi (crypt) for a same surface of tissue of TGFβR-WT *Rosa stop*$^{fl/fl}$ *Yfp* reporter mice and TGFβR-KO *Rosa stop*$^{fl/fl}$ *Yfp* reporter mice (mean $\pm$SD). Data are representative of three independent experiments Paired t-test was used ****P<0.0001, ***P<0.001, *P<0.05.

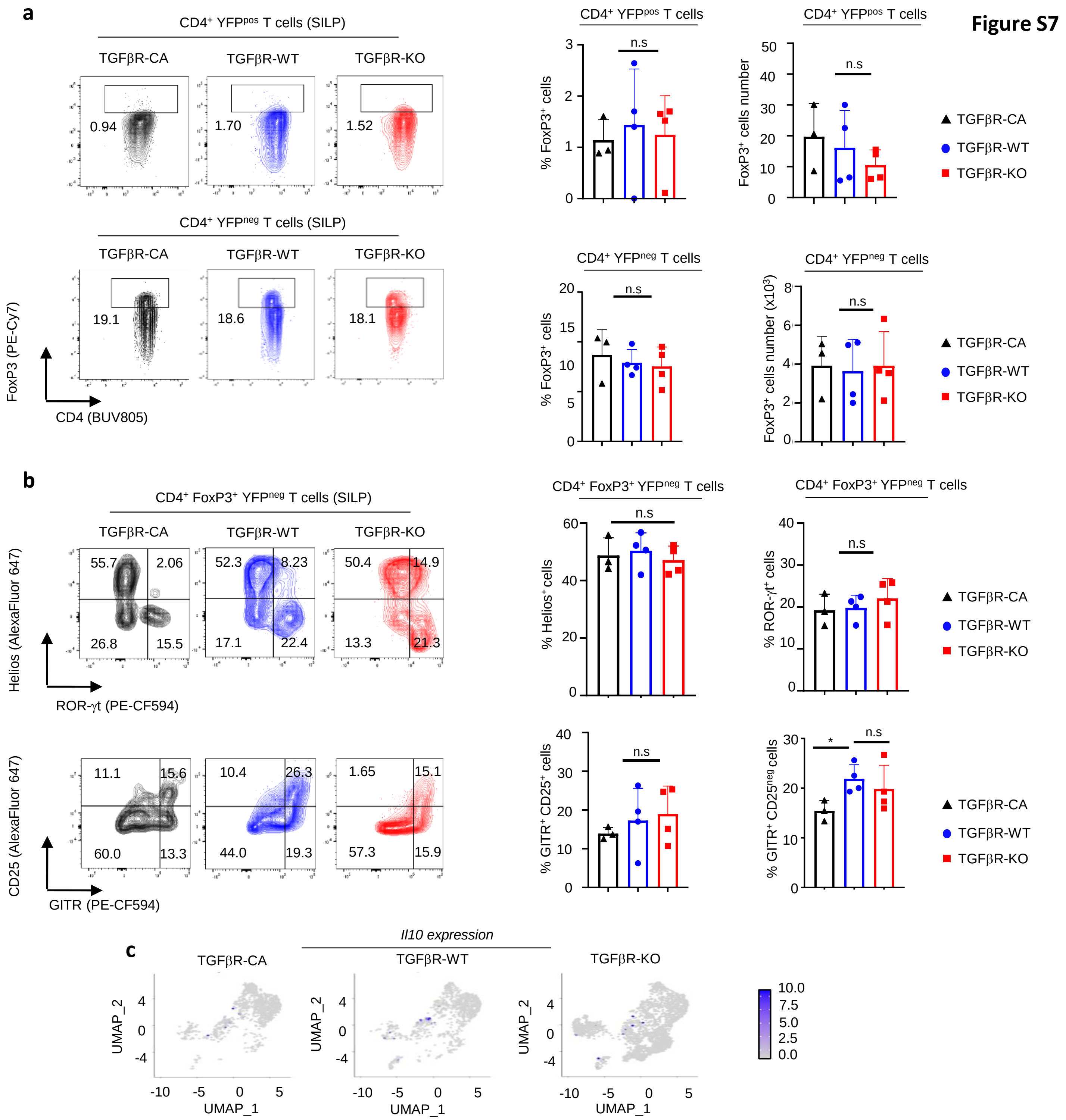

Figure S7: Treg and IL-10 producing TH17 populations are not affected in TGFβR-KO mice

At 8 month of age cells from SILP of TGFβR-CA, TGFβR-KO and TGFβR-WT mice were isolated and analyzed by flow cytometry for the presence **(A)** and the phenotype of Tregs (**B**) or purified for YFP^{pos} CD4 T cells and analyzed by single cell mRNA (C). (**A**) Representative counter plots showing the presence of Tregs among the YFP pos and YFP neg CD4 t cells. Graphs illustrate the percentage of the Tregs (mean $\pm$SD). **(B**) Representative counter plots of the Treg subset analysis and graphs illustrating the percentage of Treg subsets (mean $\pm$SD). Data were representative of 3-5 mice per groups in three independent experiments. Unpaired t-test was used n.s: not significant. (**C**) YFP CD4 a,b T cells were analyzed as in figure 4A. UMP representation of *il10* expression among YFP^{pos} CD4 TCRb cells.

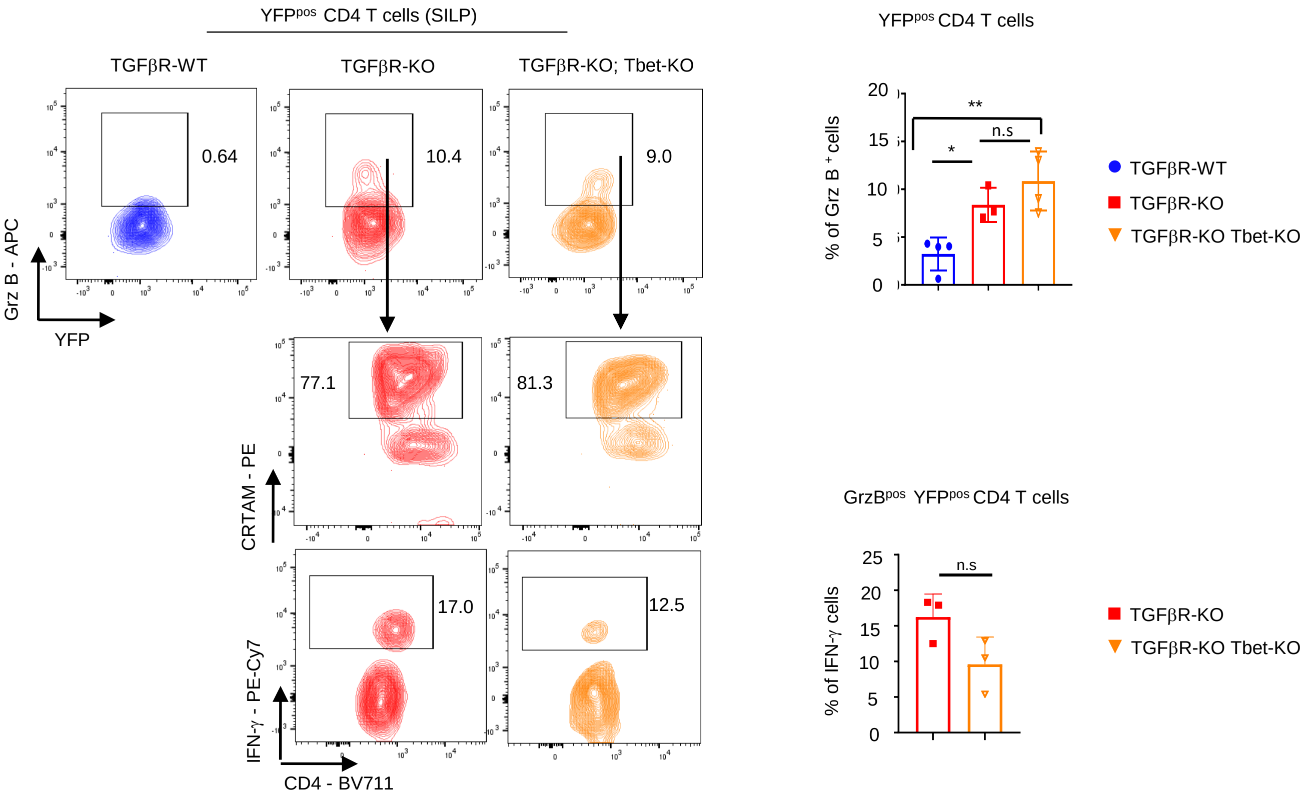

Figure S8: Differentiation of TH17 in CD4 CTL in the absence of TGF-β signaling control is T-BET independent

YFPpos CD4 T cells from SILP of 8 month-old mice were analyzed by flow cytometry for CTL features including granzyme B (GrzB), CRTAM and IFN-γ. Representative counter plots and quantification (mean ±SD) are illustrated.. Statistical analysis were performed using a two-tailed Student's t test.*P<0.05; **P<0.01; ***P<0.001 with 3 mice per group an in 2 independent experiments. n.s non statistically significant.

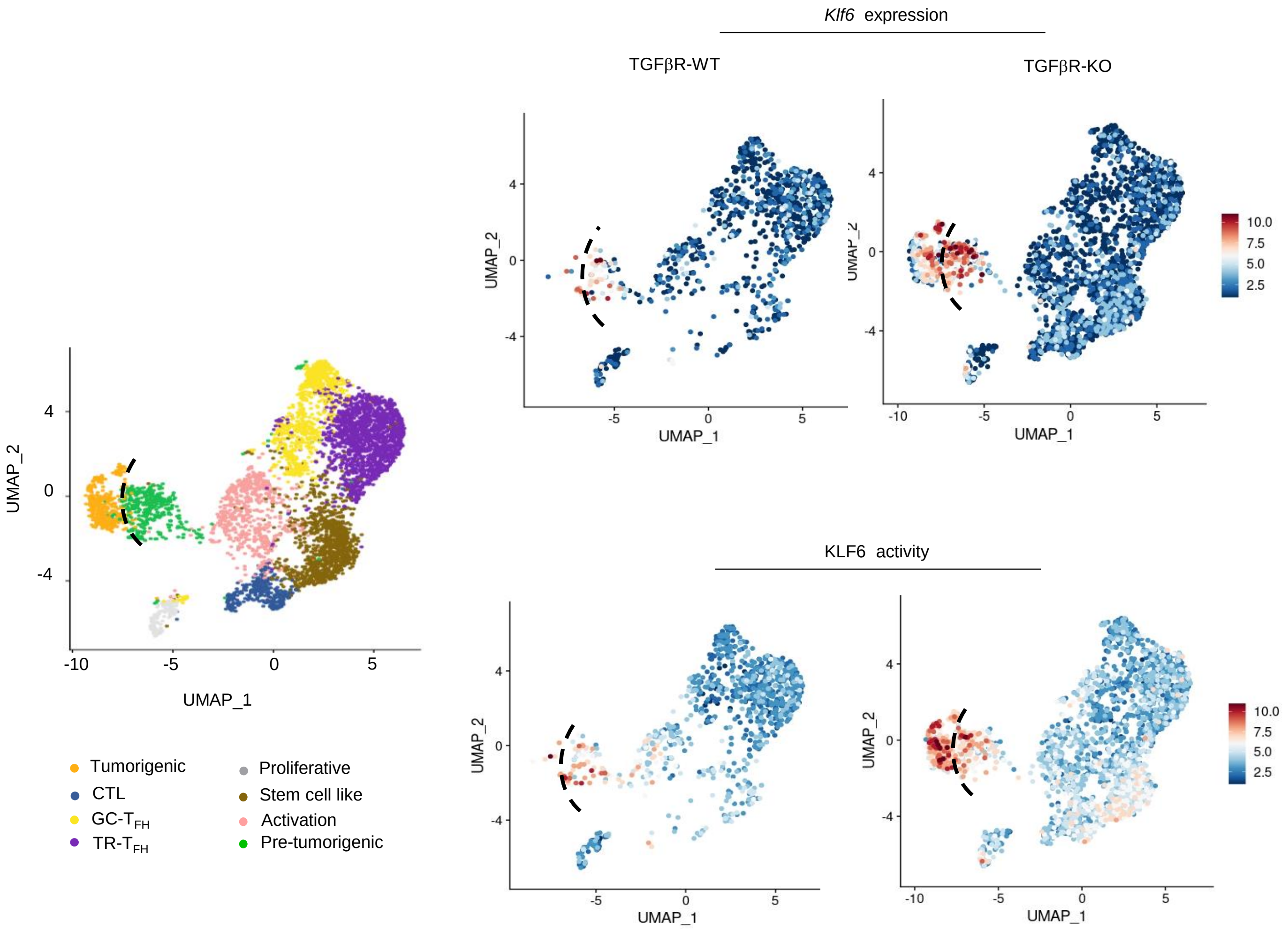

Figure S9: KLF6 activity and *Klf6* expression are restricted to pre and tumorigenic TH17 cells

Sorted YFP^{pos} CD4 T cells isolated from the small intestine of 7-8 month-old TGFβR-CA, TGFβR-KO and TGFβR-WT mice were analyzed by scRNAseq as described in Figure 4A. Feature Plots represent either the expression of *Klf6* or KLF6 regulon activity in TGFβR-WT and TGFβR-KO mice. Dash line delineates the border between pre-tumorigenic and tumorigenic clusters.

Figure S10

a

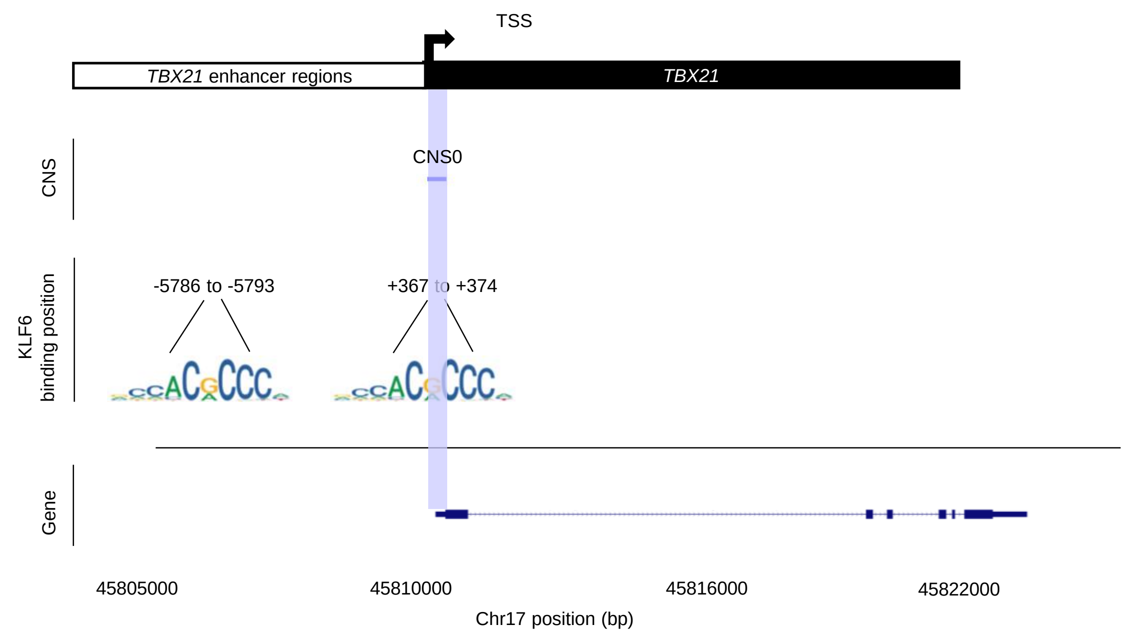

b

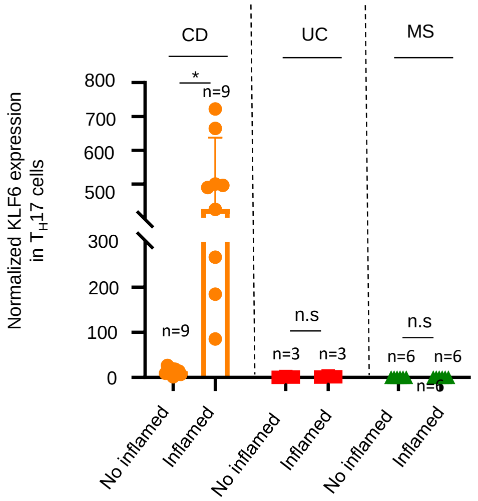

Figure S10: KLF6 and TH17 cells in human small intestine inflammation

(A) Coverage plot of *TBX21* locus is illustrated and transcription start (TSS) and direction are indicated. KLF6 binding sites are reported as well the equivalent of the CNS0 region defined in mouse *Tbx21* locus in figure 5. (**B**) TH17 cells of the small intestine and colonic biopsies from patients with Crohn disease (CD) ulcerative colitis (UC) respectively were analysis by ScRNAseq. For small intestine and colon, biopsies were taken during flare (inflamed) or not (no-inflamed). For multiple sclerosis (MS), TH17 cells were analyzed from cerebrospinal fluid of MS patients (inflamed) and heathy donors (no-inflamed). Graph illustrates the *KLF6* expression in TH17 cells (mean ±SD). Each point represents one patient sample. Statistical analysis was performed using Mann Whitney test. *P<0.05; ns: not statistically significant.

full.name	TF
Mmusculus-cisbp_1.02-M0105_1.02	M0105_1.02
Mmusculus-cisbp_1.02-M0106_1.02	M0106_1.02
Mmusculus-cisbp_1.02-M0131_1.02	M0131_1.02
Mmusculus-cisbp_1.02-M0184_1.02	M0184_1.02
Mmusculus-cisbp_1.02-M0186_1.02	M0186_1.02
Mmusculus-cisbp_1.02-M0207_1.02	M0207_1.02
Mmusculus-cisbp_1.02-M0209_1.02	M0209_1.02
Mmusculus-cisbp_1.02-M0218_1.02	M0218_1.02
Mmusculus-cisbp_1.02-M0219_1.02	M0219_1.02
Mmusculus-cisbp_1.02-M0221_1.02	M0221_1.02
Mmusculus-cisbp_1.02-M0222_1.02	M0222_1.02
Mmusculus-cisbp_1.02-M0223_1.02	M0223_1.02
Mmusculus-cisbp_1.02-M0307_1.02	M0307_1.02
Mmusculus-cisbp_1.02-M0308_1.02	M0308_1.02
Mmusculus-cisbp_1.02-M0312_1.02	M0312_1.02
Mmusculus-cisbp_1.02-M1839_1.02	M1839_1.02
Mmusculus-cisbp_1.02-M1927_1.02	M1927_1.02
Mmusculus-cisbp_1.02-M1964_1.02	M1964_1.02
Mmusculus-cisbp_1.02-M1967_1.02	M1967_1.02
Mmusculus-cisbp_1.02-M6101_1.02	M6101_1.02
Mmusculus-HOCOMOCOv10-ARI3A_MOUSE.H10MO.S	ARI3A
Mmusculus-HOCOMOCOv10-CDX1_MOUSE.H10MO.C	CDX1
Mmusculus-HOCOMOCOv10-CDX2_MOUSE.H10MO.C	CDX2
Mmusculus-HOCOMOCOv10-COT1_MOUSE.H10MO.B	COT1
Mmusculus-HOCOMOCOv10-CRX_MOUSE.H10MO.S	CRX
Mmusculus-HOCOMOCOv10-DLX2_MOUSE.H10MO.D	DLX2
Mmusculus-HOCOMOCOv10-ERR1_MOUSE.H10MO.A	ERR1
Mmusculus-HOCOMOCOv10-ERR2_MOUSE.H10MO.B	ERR2
Mmusculus-HOCOMOCOv10-ERR3_MOUSE.H10MO.B	ERR3
Mmusculus-HOCOMOCOv10-ESR2_MOUSE.H10MO.S	ESR2
Mmusculus-HOCOMOCOv10-ETV4_MOUSE.H10MO.B	ETV4
Mmusculus-HOCOMOCOv10-FOXD3_MOUSE.H10MO.D	FOXD3
Mmusculus-HOCOMOCOv10-FOXO4_MOUSE.H10MO.C	FOXO4
Mmusculus-HOCOMOCOv10-FOXP3_MOUSE.H10MO.D	FOXP3
Mmusculus-HOCOMOCOv10-GATA3_MOUSE.H10MO.C	GATA3
Mmusculus-HOCOMOCOv10-GATA6_MOUSE.H10MO.B	GATA6
Mmusculus-HOCOMOCOv10-GCR_MOUSE.H10MO.S	GCR
Mmusculus-HOCOMOCOv10-HMGA1_MOUSE.H10MO.D	HMGA1
Mmusculus-HOCOMOCOv10-HXD4_MOUSE.H10MO.D	HXD4
Mmusculus-HOCOMOCOv10-IKZF1_MOUSE.H10MO.C	IKZF1
Mmusculus-HOCOMOCOv10-ISL1_MOUSE.H10MO.D	ISL1
Mmusculus-HOCOMOCOv10-KLF6_MOUSE.H10MO.D	KLF6
Mmusculus-HOCOMOCOv10-LEF1_MOUSE.H10MO.C	LEF1
Mmusculus-HOCOMOCOv10-MAFG_MOUSE.H10MO.D	MAFG
Mmusculus-HOCOMOCOv10-MAF_MOUSE.H10MO.B	MAF
Mmusculus-HOCOMOCOv10-MECP2_MOUSE.H10MO.C	MECP2
Mmusculus-HOCOMOCOv10-MSX2_MOUSE.H10MO.D	MSX2
Mmusculus-HOCOMOCOv10-MYCN_MOUSE.H10MO.B	MYCN
Mmusculus-HOCOMOCOv10-MYF6_MOUSE.H10MO.C	MYF6
Mmusculus-HOCOMOCOv10-MYOG_MOUSE.H10MO.C	MYOG

Mmusculus-HOCOMOCOv10-NF2L1_MOUSE.H10MO.C	NF2L1
Mmusculus-HOCOMOCOv10-NFIA_MOUSE.H10MO.S	NFIA
Mmusculus-HOCOMOCOv10-NKX21_MOUSE.H10MO.D	NKX21
Mmusculus-HOCOMOCOv10-NKX25_MOUSE.H10MO.C	NKX25
Mmusculus-HOCOMOCOv10-NR4A1_MOUSE.H10MO.C	NR4A1
Mmusculus-HOCOMOCOv10-NR4A2_MOUSE.H10MO.C	NR4A2
Mmusculus-HOCOMOCOv10-PAX2_MOUSE.H10MO.S	PAX2
Mmusculus-HOCOMOCOv10-PAX5_MOUSE.H10MO.S	PAX5
Mmusculus-HOCOMOCOv10-PITX2_MOUSE.H10MO.D	PITX2
Mmusculus-HOCOMOCOv10-PPARA_MOUSE.H10MO.S	PPARA
Mmusculus-HOCOMOCOv10-PPARG_MOUSE.H10MO.S	PPARG
Mmusculus-HOCOMOCOv10-PRGR_MOUSE.H10MO.S	PRGR
Mmusculus-HOCOMOCOv10-PRRX1_MOUSE.H10MO.D	PRRX1
Mmusculus-HOCOMOCOv10-PRRX2_MOUSE.H10MO.C	PRRX2
Mmusculus-HOCOMOCOv10-RARA_MOUSE.H10MO.S	RARA
Mmusculus-HOCOMOCOv10-RARB_MOUSE.H10MO.D	RARB
Mmusculus-HOCOMOCOv10-RARG_MOUSE.H10MO.S	RARG
Mmusculus-HOCOMOCOv10-SMAD4_MOUSE.H10MO.C	SMAD4
Mmusculus-HOCOMOCOv10-SMRC1_MOUSE.H10MO.B	SMRC1
Mmusculus-HOCOMOCOv10-SNAI1_MOUSE.H10MO.C	SNAI1
Mmusculus-HOCOMOCOv10-SNAI2_MOUSE.H10MO.C	SNAI2
Mmusculus-HOCOMOCOv10-SOX10_MOUSE.H10MO.D	SOX10
Mmusculus-HOCOMOCOv10-SOX5_MOUSE.H10MO.C	SOX5
Mmusculus-HOCOMOCOv10-SP1_MOUSE.H10MO.S	SP1
Mmusculus-HOCOMOCOv10-SRY_MOUSE.H10MO.B	SRY
Mmusculus-HOCOMOCOv10-STF1_MOUSE.H10MO.B	STF1
Mmusculus-HOCOMOCOv10-TGIF1_MOUSE.H10MO.S	TGIF1
Mmusculus-HOCOMOCOv10-TWST1_MOUSE.H10MO.D	TWST1
Mmusculus-HOCOMOCOv10-UBIP1_MOUSE.H10MO.D	UBIP1
Mmusculus-HOCOMOCOv10-VDR_MOUSE.H10MO.S	VDR
Mmusculus-HOCOMOCOv10-ZEB1_MOUSE.H10MO.D	ZEB1
Mmusculus-JASPAR_CORE-Arnt-MA0004.1	Arnt
Mmusculus-JASPAR_CORE-Arnt::Ahr-MA0006.1	Arnt::Ahr
Mmusculus-JASPAR_CORE-Nkx2-5-MA0063.1	Nkx2
Mmusculus-JASPAR_CORE-Prrx2-MA0075.1	Prrx2
Mmusculus-JASPAR_CORE-Sox5-MA0087.1	Sox5
Mmusculus-JASPAR_CORE-Pdx1-MA0132.1	Pdx1
Mmusculus-JASPAR_CORE-ARID3A-MA0151.1	ARID3A
Mmusculus-JASPAR_CORE-NFATC2-MA0152.1	NFATC2
Mmusculus-JASPAR_CORE-NR4A2-MA0160.1	NR4A2
Mmusculus-JASPAR_CORE-Nr2e3-MA0164.1	Nr2e3
Mmusculus-JASPAR_CORE-SOX10-MA0442.1	SOX10
Mmusculus-JASPAR_2014-Arnt-MA0004.1	Arnt
Mmusculus-JASPAR_2014-Arnt::Ahr-MA0006.1	Arnt::Ahr
Mmusculus-JASPAR_2014-Nkx2-5-MA0063.1	Nkx2
Mmusculus-JASPAR_2014-Prrx2-MA0075.1	Prrx2
Mmusculus-JASPAR_2014-Sox5-MA0087.1	Sox5
Mmusculus-JASPAR_2014-Pdx1-MA0132.1	Pdx1
Mmusculus-JASPAR_2014-ARID3A-MA0151.1	ARID3A
Mmusculus-JASPAR_2014-Nr2e3-MA0164.1	Nr2e3
Mmusculus-JASPAR_2014-Atoh1-MA0461.1	Atoh1

Mmusculus-JASPAR_2014-Sox3-MA0514.1	Sox3
Mmusculus-JASPAR_2014-Myc-MA0147.2	Myc
Mmusculus-JASPAR_2014-Mycn-MA0104.3	Mycn
Mmusculus-JASPAR_2014-Sox2-MA0143.3	Sox2
Mmusculus-jaspar2016-Arnt-MA0004.1	Arnt
Mmusculus-jaspar2016-Ahr::Arnt-MA0006.1	Ahr::Arnt
Mmusculus-jaspar2016-Gata1-MA0035.1	Gata1
Mmusculus-jaspar2016-Klf4-MA0039.1	Klf4
Mmusculus-jaspar2016-Nkx2-5-MA0063.1	Nkx2
Mmusculus-jaspar2016-Prrx2-MA0075.1	Prrx2
Mmusculus-jaspar2016-Prrx2-MA0075.2	Prrx2
Mmusculus-jaspar2016-Sox5-MA0087.1	Sox5
Mmusculus-jaspar2016-JUN::FOS-MA0099.1	JUN::FOS
Mmusculus-jaspar2016-FOS::JUN-MA0099.2	FOS::JUN
Mmusculus-jaspar2016-Mycn-MA0104.1	Mycn
Mmusculus-jaspar2016-Mycn-MA0104.3	Mycn
Mmusculus-jaspar2016-Pdx1-MA0132.1	Pdx1
Mmusculus-jaspar2016-Sox2-MA0143.3	Sox2
Mmusculus-jaspar2016-Myc-MA0147.2	Myc
Mmusculus-jaspar2016-Arid3a-MA0151.1	Arid3a
Mmusculus-jaspar2016-NR4A2-MA0160.1	NR4A2
Mmusculus-jaspar2016-Nr2e3-MA0164.1	Nr2e3
Mmusculus-jaspar2016-Atoh1-MA0461.1	Atoh1
Mmusculus-jaspar2016-Sox3-MA0514.1	Sox3
Mmusculus-jaspar2016-Bhlha15-MA0607.1	Bhlha15
Mmusculus-jaspar2016-Dux-MA0611.1	Dux
Mmusculus-jaspar2016-Pitx1-MA0682.1	Pitx1
Mmusculus-jaspar2016-Lhx4-MA0704.1	Lhx4
Mmusculus-jaspar2016-Msx3-MA0709.1	Msx3
Mmusculus-jaspar2016-Shox2-MA0720.1	Shox2
Mmusculus-jaspar2016-Dlx3-MA0880.1	Dlx3
Mmusculus-jaspar2016-Dlx4-MA0881.1	Dlx4
Mmusculus-jaspar2016-Dlx2-MA0885.1	Dlx2
Mmusculus-jaspar2018-Arnt-MA0004.1	Arnt
Mmusculus-jaspar2018-Ahr::Arnt-MA0006.1	Ahr::Arnt
Mmusculus-jaspar2018-Gata1-MA0035.1	Gata1
Mmusculus-jaspar2018-Klf4-MA0039.1	Klf4
Mmusculus-jaspar2018-Nkx2-5-MA0063.1	Nkx2
Mmusculus-jaspar2018-Prrx2-MA0075.1	Prrx2
Mmusculus-jaspar2018-Sox5-MA0087.1	Sox5
Mmusculus-jaspar2018-JUN::FOS-MA0099.1	JUN::FOS
Mmusculus-jaspar2018-Mycn-MA0104.1	Mycn
Mmusculus-jaspar2018-Pdx1-MA0132.1	Pdx1
Mmusculus-jaspar2018-Arid3a-MA0151.1	Arid3a
Mmusculus;Rnorvegicus;Hsapiens-jaspar2018-NFATC2-MA0152.1	NFATC2
Mmusculus;Rnorvegicus;Hsapiens-jaspar2018-NR4A2-MA0160.1	NR4A2
Mmusculus-jaspar2018-Nr2e3-MA0164.1	Nr2e3
Mmusculus;Rnorvegicus;Hsapiens-jaspar2018-FOS::JUN-MA0099.2	FOS::JUN
Mmusculus;Rnorvegicus;Hsapiens-jaspar2018-SP1-MA0079.2	SP1
Mmusculus;Rnorvegicus;Hsapiens-jaspar2018-SOX10-MA0442.1	SOX10
Mmusculus-jaspar2018-Sox3-MA0514.1	Sox3

Mmusculus-jaspar2018-Atoh1-MA0461.1	Atoh1
Mmusculus-jaspar2018-Myc-MA0147.2	Myc
Mmusculus-jaspar2018-Mycn-MA0104.3	Mycn
Mmusculus-jaspar2018-Sox2-MA0143.3	Sox2
Mmusculus-jaspar2018-Bhlha15-MA0607.1	Bhlha15
Mmusculus-jaspar2018-Dux-MA0611.1	Dux
Mmusculus-jaspar2018-Pitx1-MA0682.1	Pitx1
Mmusculus-jaspar2018-Prrx2-MA0075.2	Prrx2
Mmusculus-jaspar2018-Lhx4-MA0704.1	Lhx4
Mmusculus-jaspar2018-Msx3-MA0709.1	Msx3
Mmusculus-jaspar2018-Shox2-MA0720.1	Shox2
Mmusculus-jaspar2018-Dlx3-MA0880.1	Dlx3
Mmusculus-jaspar2018-Dlx4-MA0881.1	Dlx4
Mmusculus-jaspar2018-Dlx2-MA0885.1	Dlx2
Mmusculus-jaspar2018-Smad4-MA1153.1	Smad4
Mmusculus-jaspar2022-Arnt-MA0004.1	Arnt
Mmusculus-jaspar2022-Ahr::Arnt-MA0006.1	Ahr::Arnt
Mmusculus-jaspar2022-Arid3a-MA0151.1	Arid3a
Mmusculus-jaspar2022-Nr2e3-MA0164.1	Nr2e3
Mmusculus-jaspar2022-Lhx4-MA0704.1	Lhx4
Mmusculus-jaspar2022-Msx3-MA0709.1	Msx3
Mmusculus-jaspar2022-Shox2-MA0720.1	Shox2
Mmusculus-jaspar2022-Dlx3-MA0880.1	Dlx3
Mmusculus-jaspar2022-Dlx4-MA0881.1	Dlx4
Mmusculus-jaspar2022-Smad4-MA1153.1	Smad4
Mmusculus-jaspar2022-Foxn1-MA1684.1	Foxn1
Mmusculus-jaspar2022-Neurod2-MA1993.1	Neurod2
Mmusculus-jaspar2022-Nr1H2-MA1996.1	Nr1H2
Mmusculus-jaspar2022-Olig2-MA1997.1	Olig2
Mmusculus-jaspar2022-Twist2-MA0633.2	Twist2
Mmusculus-jaspar2022-Vdr-MA0693.3	Vdr
Mmusculus-jolma2013-Tcfap2a-2	Tcfap2a
Mmusculus-jolma2013-Foxc1-2	Foxc1
Mmusculus-jolma2013-Dlx1	Dlx1
Mmusculus-jolma2013-Dlx2	Dlx2
Mmusculus-jolma2013-Lhx4	Lhx4
Mmusculus-jolma2013-Meox2	Meox2
Mmusculus-jolma2013-Msx3-2	Msx3
Mmusculus-jolma2013-Shox2	Shox2

Supplementary Table 1 : Tbx21.locus_predicted.TFs

DNA sequence was extracted for the mouse *Tbx21* locus (mm10: chr11:97,098,071-97,115,331) and extended by 15kb up- and downstream. This sequence was screened for transcription factor binding sites using sequence matching. All significant matches are shown.

Supplementary Table 2

motif	observed	background	percent.observed	percent.background	fold.enrichment	pvalue	motif.name	p.adjust
MA0006.1	3522	7743	35,75271546	19,3575	1,846969674	0	Ahr::Arnt	0
MA0151.1	6980	17948	70,85575069	44,87	1,579134181	0	Arid3a	0
MA0004.1	2929	6573	29,73302203	16,4325	1,80940344	0	Arnt	0
MA1100.2	2843	6225	28,86001421	15,5625	1,854458745	0	ASCL1	0
MA1631.1	4032	9522	40,92985484	23,805	1,719380585	0	ASCL1(var.2)	0
MA0816.1	3241	7193	32,90021318	17,9825	1,829568368	0	Ascl2	0
MA1467.1	3024	6655	30,69739113	16,6375	1,845072344	0	ATOH1(var.2)	0
MA1633.1	3606	8222	36,60542077	20,555	1,780852385	0	BACH1	0
MA0591.1	3213	7292	32,61597807	18,23	1,789137579	0	Bach1::Mafk	0
MA1634.1	3249	7338	32,98142321	18,345	1,797842639	0	BATF	0
MA0462.2	3242	7311	32,91036443	18,2775	1,800594416	0	BATF::JUN	0
MA0835.2	3268	7360	33,17429703	18,4	1,802950925	0	BATF3	0
MA0463.2	2835	6220	28,77880418	15,55	1,850726957	0	BCL6	0
MA1472.1	2747	5942	27,88549386	14,855	1,877178987	0	BHLHA15(var.2)	0
MA1635.1	2822	6003	28,64683788	15,0075	1,908834775	0	BHLHE22(var.2)	0
MA0465.2	2662	5862	27,0226373	14,655	1,843919297	0	CDX2	0
MA0018.4	2538	5232	25,76388184	13,08	1,969715737	0	CREB1	0
MA0471.2	4177	9334	42,40178662	23,335	1,817089634	0	E2F6	0
MA0154.4	3266	7037	33,15399452	17,5925	1,884552765	0	EBF1	0
MA1604.1	3135	6735	31,82418029	16,8375	1,890077523	0	Ebf2	0
MA1637.1	3158	6667	32,05765912	16,6675	1,923363379	0	EBF3	0
MA0162.4	4233	9422	42,97025683	23,555	1,824252041	0	EGR1	0
MA0732.1	2890	6324	29,33712313	15,81	1,855605511	0	EGR3	0
MA0733.1	2628	5729	26,67749467	14,3225	1,862628359	0	EGR4	0
MA0598.3	5599	13788	56,83686935	34,47	1,648879297	0	EHF	0
MA0473.3	5782	14218	58,69454878	35,545	1,651274406	0	ELF1	0
MA0640.2	5788	14135	58,7554563	35,3375	1,6626942	0	ELF3	0
MA0136.2	3221	7505	32,6971881	18,7625	1,74268824	0	ELF5	0
MA0076.2	2988	6930	30,331946	17,325	1,750761674	0	ELK4	2,44E-307
MA0800.1	2668	4837	27,08354482	12,0925	2,239697731	0	EOMES	0
MA0258.2	3307	7476	33,57019592	18,69	1,796158155	0	ESR2	0
MA0592.3	3032	6838	30,77860116	17,095	1,800444642	0	ESRRA	0
MA0141.3	2719	5885	27,60125876	14,7125	1,876041377	0	ESRRB	0
MA0643.1	2600	5587	26,39325957	13,9675	1,889619443	0	Esrrg	0
MA0761.2	6011	14874	61,01918587	37,185	1,640962374	0	ETV1	0
MA0764.2	6235	15414	63,29306669	38,535	1,642482592	0	ETV4	0
MA0645.1	3108	7204	31,55009644	18,01	1,751809908	0	ETV6	0

Supplementary Table 2

MA0149.1	6466	16024	65,63800629	40,06	1,638492419	0	EWSR1-FLI1	0
MA0476.1	2907	6524	29,50969445	16,31	1,809300702	0	FOS	0
MA0099.3	2653	5851	26,93127601	14,6275	1,841140045	0	FOS::JUN	0
MA1141.1	2851	6350	28,94122424	15,875	1,823069244	0	FOS::JUND	0
MA0477.2	2755	6109	27,96670389	15,2725	1,83118048	0	FOSL1	0
MA1128.1	2854	6268	28,971678	15,67	1,848862668	0	FOSL1::JUN	0
MA1137.1	2604	5638	26,43386458	14,095	1,875407207	0	FOSL1::JUNB	0
MA0478.1	2806	6320	28,48441783	15,8	1,802811255	0	FOSL2	0
MA1130.1	2632	5795	26,71809969	14,4875	1,844217407	0	FOSL2::JUN	0
MA0148.4	2692	5942	27,32717491	14,855	1,839594406	0	FOXA1	0
MA0047.3	2922	6421	29,66196325	16,0525	1,847809578	0	FOXA2	0
MA1683.1	2773	6123	28,14942645	15,3075	1,838930358	0	FOXA3	0
MA0845.1	3464	7680	35,16394275	19,2	1,831455351	0	FOXB1	0
MA0032.2	3586	8043	36,4023957	20,1075	1,810388944	0	FOXC1	0
MA0846.1	4769	11019	48,4113288	27,5475	1,757376488	0	FOXC2	0
MA0847.2	3840	8731	38,98081413	21,8275	1,785857937	0	FOXD2	0
MA0041.1	5336	12442	54,16708964	31,105	1,74142709	0	Foxd3	0
MA1487.1	3932	8962	39,91472947	22,405	1,781509907	0	FOXE1	0
MA1606.1	3144	6949	31,91554157	17,3725	1,837130037	0	Foxf1	0
MA0030.1	2476	5195	25,13450411	12,9875	1,935284243	0	FOXF2	0
MA0042.2	2659	5881	26,99218353	14,7025	1,835890735	0	FOXI1	0
MA0851.1	4244	9691	43,08192062	24,2275	1,778223945	0	Foxj3	0
MA0852.2	2662	5814	27,0226373	14,535	1,859142573	0	FOXK1	0
MA1103.2	2694	5926	27,34747741	14,815	1,845931651	0	FOXK2	0
MA0033.2	2659	5881	26,99218353	14,7025	1,835890735	0	FOXL1	0
MA1607.1	3440	7544	34,92031266	18,86	1,851554224	0	Foxl2	0
MA0480.1	3657	8173	37,12313471	20,4325	1,816866987	0	Foxo1	0
MA0848.1	2659	5881	26,99218353	14,7025	1,835890735	0	FOXO4	0
MA0849.1	2659	5881	26,99218353	14,7025	1,835890735	0	FOXO6	0
MA0481.3	2973	6590	30,17967719	16,475	1,83184687	0	FOXP1	0
MA0593.1	3392	7481	34,43305248	18,7025	1,841093569	0	FOXP2	0
MA0850.1	2659	5881	26,99218353	14,7025	1,835890735	0	FOXP3	0
MA0040.1	2657	5704	26,97188103	14,26	1,891436257	0	Foxq1	0
MA0062.3	5956	14857	60,46086692	37,1425	1,627808223	0	GABPA	0
MA0483.1	3331	7426	33,81382601	18,565	1,821374953	0	Gfi1b	0
MA0734.2	3107	6433	31,53994518	16,0825	1,961134474	0	GLI2	0
MA1491.1	3041	6257	30,86996244	15,6425	1,973467313	0	GLI3	0
MA1638.1	2857	6279	29,00213176	15,6975	1,847563737	0	HAND2	0

Supplementary Table 2

MA0738.1	2623	5267	26,6267384	13,1675	2,022155945		0	HIC2		0
MA0899.1	2688	5728	27,28656989	14,32	1,905486724		0	HOXA10		0
MA0901.2	3749	8444	38,05705005	21,11	1,802797255		0	HOXB13		0
MA0909.2	2113	4147	21,44959903	10,3675	2,068926841		0	HOXD13		0
MA0770.1	2406	5110	24,42391635	12,775	1,911852552	0		HSF2	0	
MA1508.1	6309	15755	64,04425947	39,3875	1,626004683		0	IKZF1		0
MA0155.1	3092	6608	31,38767638	16,52	1,89998041		0	INSM1		0
MA0050.2	7222	18590	73,31235408	46,475	1,577457861		0	IRF1		0
MA0051.1	3313	7361	33,63110344	18,4025	1,827529055		0	IRF2		0
MA1418.1	4341	9841	44,06659222	24,6025	1,79114286		0	IRF3		0
MA1419.1	2491	5192	25,28677292	12,98	1,948133507		0	IRF4		0
MA0772.1	3823	8528	38,80824282	21,32	1,820274053		0	IRF7		0
MA0652.1	2579	5435	26,18008324	13,5875	1,926777055		0	IRF8		0
MA0653.1	3586	7784	36,4023957	19,46	1,870626706		0	IRF9		0
MA0489.1	3002	6635	30,47406355	16,5875	1,837170372		0	JUN(var.2)		0
MA0493.1	5013	10987	50,8882347	27,4675	1,852670782		0	Klf1		0
MA1511.1	5100	11678	51,77139377	29,195	1,773296584		0	KLF10		0
MA1512.1	4880	11379	49,53811796	28,4475	1,741387396		0	KLF11		0
MA0742.1	3474	7408	35,26545528	18,52	1,904182251		0	Klf12		0
MA1513.1	2941	6726	29,85483707	16,815	1,775488378	0		KLF15	0	
MA0741.1	5764	13951	58,51182621	34,8775	1,677638197		0	KLF16		0
MA1514.1	4394	9721	44,60460867	24,3025	1,835391777		0	KLF17		0
MA1515.1	4968	10975	50,43142828	27,4375	1,8380475		0	KLF2		0
MA1516.1	3282	6852	33,31641458	17,13	1,944916204		0	KLF3		0
MA0039.4	6526	16074	66,24708151	40,185	1,648552483		0	KLF4		0
MA0599.1	6299	15202	63,94274693	38,005	1,682482487		0	KLF5		0
MA1517.1	3742	8147	37,98599127	20,3675	1,865029644		0	KLF6		0
MA1107.2	6852	16798	69,55639021	41,995	1,656301708		0	KLF9		0
MA0768.1	3654	8386	37,09268095	20,965	1,769266918		0	LEF1		0
MA0501.1	2818	6261	28,60623287	15,6525	1,827582359		0	MAF::NFE2		0
MA0659.2	2806	6321	28,48441783	15,8025	1,802526045	0		MAFG	0	
MA0496.3	3744	8731	38,00629378	21,8275	1,741211489		0	MAFK		0
MA1522.1	4841	11045	49,14221906	27,6125	1,779709156		0	MAZ		0
MA0052.4	4066	9243	41,27499746	23,1075	1,786216487		0	MEF2A		0
MA0497.1	4141	9498	42,03634149	23,745	1,77032392		0	MEF2C		0
MA0773.1	2612	5424	26,51507461	13,56	1,955388983		0	MEF2D		0
MA0498.2	2675	5832	27,15460359	14,58	1,862455665		0	MEIS1		0
MA0801.1	2746	5164	27,8753426	12,91	2,159205469		0	MGA		0

Supplementary Table 2

MA1523.1	2448	5158	24,85026901	12,895	1,92712439		0	MSANTD3		0
MA1108.2	3105	6846	31,51964268	17,115	1,841638485		0	MXI1		0
MA1641.1	2478	5205	25,15480662	13,0125	1,933126349		0	MYF5		0
MA0499.2	3264	7426	33,13369201	18,565	1,784739672		0	MYOD1		0
MA0500.2	2536	5500	25,74357933	13,75	1,872260315	0		MYOG	0	
MA0056.2	2586	5505	26,25114202	13,7625	1,907439929		0	MZF1		0
MA0057.1	3327	7382	33,77322099	18,455	1,83003094		0	MZF1(var.2)		0
MA1109.1	3425	7799	34,76804385	19,4975	1,783205224		0	NEUROD1		0
MA1642.1	3375	7699	34,26048117	19,2475	1,779996424		0	NEUROG2(var.2)		0
MA0606.1	3045	6774	30,91056746	16,935	1,825247562		0	NFAT5		0
MA0152.1	3961	9240	40,20911583	23,1	1,740654365		0	NFATC2		0
MA0089.2	3079	7041	31,25571008	17,6025	1,775640397		0	NFE2L1		0
MA0150.2	2839	6323	28,8194092	15,8075	1,823147822		0	Nfe2l2		0
MA1643.1	2824	6065	28,66714039	15,1625	1,890660537		0	NFIB		0
MA0161.2	2776	6118	28,17988022	15,295	1,842424336		0	NFIC		0
MA0048.2	2876	6070	29,19500558	15,175	1,923888342		0	NHLH1		0
MA1529.1	2623	5437	26,6267384	13,5925	1,958928703		0	NHLH2		0
MA0503.1	2960	6704	30,04771089	16,76	1,792822846		0	Nkx2-5(var.2)		0
MA0504.1	3966	8732	40,25987209	21,83	1,844245172		0	NR2C2		0
MA0017.2	2982	6572	30,27103847	16,43	1,84242474		0	NR2F1		0
MA1537.1	3075	6745	31,21510507	16,8625	1,85115523		0	NR2F1(var.2)		0
MA1111.1	2928	6478	29,72287077	16,195	1,835311564		0	NR2F2		0
MA0677.1	3189	7171	32,37234798	17,9275	1,805736884		0	Nr2f6		0
MA0728.1	3178	7247	32,26068419	18,1175	1,780636633		0	Nr2f6(var.2)		0
MA1112.2	3126	7058	31,732819	17,645	1,798402891		0	NR4A1		0
MA0160.1	3030	6652	30,75829865	16,63	1,849566966		0	NR4A2		0
MA1540.1	3498	7943	35,50908537	19,8575	1,788195159		0	NR5A1		0
MA0505.1	4625	10769	46,94954827	26,9225	1,743877733		0	Nr5a2		0
MA1541.1	3184	7045	32,32159172	17,6125	1,835150701		0	NR6A1		0
MA0679.2	3017	6651	30,62633235	16,6275	1,841908426		0	ONECUT1		0
MA0757.1	2590	5432	26,29174703	13,58	1,936063846		0	ONECUT3		0
MA1646.1	4174	9744	42,37133286	24,36	1,73938148		0	OSR2		0
MA1113.2	2450	5225	24,87057152	13,0625	1,903967197	0		PBX2	0	
MA1114.1	3529	7816	35,82377424	19,54	1,833355897		0	PBX3		0
MA0681.2	3279	7109	33,28596082	17,7725	1,872891311		0	PHOX2B		0
MA0782.2	3190	7054	32,38249924	17,635	1,83626307		0	PKNOX1		0
MA0783.1	2818	5949	28,60623287	14,8725	1,923431358		0	PKNOX2		0
MA0163.1	3621	7738	36,75768957	19,345	1,900113186		0	PLAG1		0

Supplementary Table 2

MA1615.1	3628	7735	36,82874835	19,3375	1,904524802		0	Plagl1		0
MA0784.1	2712	5809	27,53019998	14,5225	1,895692889		0	POU1F1		0
MA0507.1	3114	6714	31,61100396	16,785	1,883288886		0	POU2F2		0
MA0627.2	2900	6354	29,43863567	15,885	1,853234855		0	POU2F3		0
MA0786.1	2787	6165	28,29154401	15,4125	1,835623293		0	POU3F1		0
MA0787.1	2557	5488	25,95675566	13,72	1,891891812		0	POU3F2		0
MA0788.1	2650	5643	26,90082225	14,1075	1,906845455		0	POU3F3		0
MA0790.1	2643	5678	26,82976348	14,195	1,890085486		0	POU4F1		0
MA0791.1	2510	5340	25,47964674	13,35	1,908587771		0	POU4F3		0
MA1115.1	2878	6521	29,21530809	16,3025	1,792075331	0		POU5F1	0	
MA0142.1	3156	7004	32,03735661	17,51	1,829660572		0	Pou5f1::Sox2		0
MA1148.1	2845	6364	28,88031672	15,91	1,815230466		0	PPARA::RXRA		0
MA1550.1	2982	6377	30,27103847	15,9425	1,898763586		0	PPARD		0
MA0065.2	4344	9850	44,09704599	24,625	1,790742984		0	Pparg::Rxra		0
MA0508.3	5043	11493	51,19277231	28,7325	1,781702682		0	PRDM1		0
MA1616.1	3320	7346	33,70216222	18,365	1,835129987		0	Prdm15		0
MA1647.1	4045	9300	41,06182113	23,25	1,766099834		0	PRDM4		0
MA1618.1	3223	7230	32,71749061	18,075	1,810096299		0	Ptf1a		0
MA1619.1	2519	5317	25,57100802	13,2925	1,923716985		0	Ptf1a(var.2)		0
MA1620.1	3304	7229	33,53974216	18,0725	1,855844081		0	Ptf1a(var.3)		0
MA0729.1	2944	6683	29,88529083	16,7075	1,788735049		0	RARA		0
MA0159.1	2713	5875	27,54035123	14,6875	1,875087744		0	RARA::RXRA		0
MA0857.1	3128	7031	31,75312151	17,5775	1,806464031		0	Rarb		0
MA0859.1	2825	6401	28,67729165	16,0025	1,79205072	0		Rarg	0	
MA1116.1	3787	8699	38,44279769	21,7475	1,767688134		0	RBPJ		0
MA1621.1	3266	7210	33,15399452	18,025	1,839333954		0	Rbpjl		0
MA0138.2	3256	6989	33,05248198	17,4725	1,891685905		0	REST		0
MA0509.2	2610	5613	26,4947721	14,0325	1,888100631		0	RFX1		0
MA0798.2	2597	5599	26,36280581	13,9975	1,883393878		0	RFX3		0
MA0071.1	2813	6273	28,5554766	15,6825	1,820849775		0	RORA		0
MA0073.1	7111	17609	72,18556492	44,0225	1,639742516		0	RREB1		0
MA0002.2	4867	11026	49,40615166	27,565	1,792350867		0	RUNX1		0
MA0511.2	2553	5274	25,91615064	13,185	1,965578358		0	RUNX2		0
MA0684.2	3283	7274	33,32656583	18,185	1,832640409		0	RUNX3		0
MA0512.2	2966	6523	30,10861841	16,3075	1,846304977		0	Rxra		0
MA0855.1	3044	6564	30,9004162	16,41	1,883023535		0	RXRB		0
MA0856.1	2978	6470	30,23043346	16,175	1,868960337		0	RXRG		0
MA1622.1	3205	7186	32,53476804	17,965	1,811008519		0	Smad2::Smad3		0

Supplementary Table 2

MA0513.1	3992	8872	40,52380469	22,18	1,827042592	0	SMAD2::SMAD3::SMAD4	0
MA1558.1	3522	8189	35,75271546	20,4725	1,746377602	0	SNAI1	0
MA0745.2	3056	6723	31,02223125	16,8075	1,845737394	0	SNAI2	0
MA1559.1	3194	7263	32,42310425	18,1575	1,785659053	0	SNAI3	0
MA0442.2	3787	8619	38,44279769	21,5475	1,784095495	0	SOX10	0
MA1152.1	4106	9317	41,68104761	23,2925	1,789462171	0	SOX15	0
MA0514.1	5024	12113	50,99989849	30,2825	1,684137653	0	Sox3	0
MA0867.2	3382	7616	34,33153995	19,04	1,803127098	0	SOX4	0
MA0087.1	3028	6862	30,73799614	17,155	1,791780597	0	Sox5	0
MA0515.1	3572	7941	36,26027814	19,8525	1,826484228	0	Sox6	0
MA0079.4	4302	10054	43,67069333	25,135	1,737445527	0	SP1	0
MA0516.2	4934	11835	50,08628566	29,5875	1,692819118	0	SP2	0
MA0746.2	4599	10716	46,68561567	26,79	1,742650828	0	SP3	0
MA0685.1	3997	9318	40,57456096	23,295	1,741771237	0	SP4	0
MA0747.1	5186	12208	52,64440158	30,52	1,724914862	0	SP8	0
MA1564.1	4678	11102	47,48756471	27,755	1,710955313	0	SP9	0
MA0080.5	6835	17200	69,3838189	43	1,613577184	0	SPI1	0
MA0081.2	6113	14986	62,05461374	37,465	1,656335613	0	SPIB	0
MA0687.1	3907	8974	39,66094813	22,435	1,767815829	0	SPIC	0
MA0595.1	2208	4290	22,41396813	10,725	2,089880478	0	SREBF1	0
MA0596.1	2280	4602	23,14485839	11,505	2,01172172	0	SREBF2	0
MA0137.3	3167	6915	32,1490204	17,2875	1,85966857	0	STAT1	0
MA0517.1	5854	14184	59,42543904	35,46	1,675844305	0	STAT1::STAT2	0
MA1623.1	6506	16205	66,04405644	40,5125	1,63021429	0	Stat2	0
MA0144.2	4164	9603	42,26982032	24,0075	1,760692297	0	STAT3	0
MA0518.1	3796	8413	38,53415897	21,0325	1,83212452	0	Stat4	0
MA1624.1	3159	6759	32,06781037	16,8975	1,89778431	0	Stat5a	0
MA0519.1	3381	7544	34,32138869	18,86	1,819797916	0	Stat5a::Stat5b	0
MA1625.1	3386	7477	34,37214496	18,6925	1,838820113	0	Stat5b	0
MA0520.1	3161	6996	32,08811288	17,49	1,834654825	0	Stat6	0
MA0802.1	2600	4728	26,39325957	11,82	2,232932282	0	TBR1	0
MA0805.1	2712	5199	27,53019998	12,9975	2,118115021	0	TBX1	0
MA0803.1	2746	5164	27,8753426	12,91	2,159205469	0	TBX15	0
MA1565.1	3406	6831	34,57517003	17,0775	2,02460372	0	TBX18	0
MA0688.1	2606	4913	26,45416709	12,2825	2,153809655	0	TBX2	0
MA0689.1	2641	5013	26,80946097	12,5325	2,139194971	0	TBX20	0
MA0690.1	2722	5006	27,63171252	12,515	2,207887536	0	TBX21	0
MA1566.1	3049	5934	30,95117247	14,835	2,086361474	0	TBX3	0

Supplementary Table 2

MA0806.1	2559	5013	25,97705817	12,5325	2,072775437		0	TBX4		0
MA0807.1	3357	6632	34,0777586	16,58	2,055353354		0	TBX5		0
MA1567.1	2594	4868	26,33235205	12,17	2,163710111		0	TBX6		0
MA0521.1	4040	9076	41,01106487	22,69	1,807451074		0	Tcf12		0
MA1648.1	3756	8833	38,12810882	22,0825	1,726621027		0	TCF12(var.2)		0
MA0522.3	3725	8753	37,81341996	21,8825	1,728021019		0	TCF3		0
MA0830.2	3668	8592	37,2347985	21,48	1,733463617		0	TCF4		0
MA0769.2	3119	7125	31,66176023	17,8125	1,777502329		0	TCF7		0
MA1421.1	3514	7998	35,67150543	19,995	1,784021277		0	TCF7L1		0
MA0523.1	3789	8725	38,46310019	21,8125	1,763351298		0	TCF7L2		0
MA0003.4	3490	7467	35,42787534	18,6675	1,897837168		0	TFAP2A		0
MA0810.1	2563	5185	26,01766318	12,9625	2,007148558		0	TFAP2A(var.2)		0
MA0811.1	2262	4590	22,96213582	11,475	2,001057588		0	TFAP2B		0
MA0812.1	2314	4540	23,49000102	11,35	2,069603614		0	TFAP2B(var.2)		0
MA0524.2	2250	4501	22,84032078	11,2525	2,029799669		0	TFAP2C		0
MA0814.2	3444	7318	34,96091767	18,295	1,910954779		0	TFAP2C(var.2)		0
MA1569.1	2063	3908	20,94203634	9,77	2,143504231		0	TFAP2E		0
MA0597.1	2884	6061	29,27621561	15,1525	1,932104644		0	THAP1		0
MA1574.1	2988	6292	30,331946	15,73	1,928286459		0	THRB		0
MA1576.1	3265	7277	33,14384326	18,1925	1,821841048		0	THRB(var.3)		0
MA1123.2	3054	6557	31,00192874	16,3925	1,891226399		0	TWIST1		0
MA0093.3	2500	5349	25,3781342	13,3725	1,897785321	0		USF1	0	
MA0693.2	3418	7798	34,69698508	19,495	1,779788924		0	VDR		0
MA1578.1	3420	7428	34,71728759	18,57	1,869536219		0	VEZF1		0
MA1627.1	5651	13475	57,36473454	33,6875	1,702849263		0	Wt1		0
MA0095.2	2950	6533	29,94619836	16,3325	1,833534263		0	YY1		0
MA1649.1	3953	9232	40,1279058	23,08	1,738644099		0	ZBTB12		0
MA0698.1	3480	7716	35,32636281	19,29	1,831330368		0	ZBTB18		0
MA1579.1	3652	8198	37,07237844	20,495	1,808849887		0	ZBTB26		0
MA1581.1	3211	6866	32,59567557	17,165	1,898961583		0	ZBTB6		0
MA0750.2	3287	7493	33,36717085	18,7325	1,78124494		0	ZBTB7A		0
MA0103.3	3741	8578	37,97584002	21,445	1,770848217		0	ZEB1		0
MA1651.1	2947	6581	29,91574459	16,4525	1,818309959		0	ZFP42		0
MA0146.2	3381	7016	34,32138869	17,54	1,95674964		0	Zfx		0
MA1628.1	4539	11039	46,07654045	27,5975	1,669591103		0	Zic1::Zic2		0
MA1629.1	4487	10716	45,54867526	26,79	1,700211843		0	Zic2		0
MA0697.1	2409	5125	24,45437011	12,8125	1,908633765	0		ZIC3	0	
MA1652.1	5289	12008	53,68998071	30,02	1,788473708		0	ZKSCAN5		0

Supplementary Table 2

MA1587.1	3937	8685	39,96548574	21,7125	1,840667161	0	ZNF135	0
MA0088.2	2775	6032	28,16972896	15,08	1,868019162	0	ZNF143	0
MA1653.1	5833	14249	59,21226271	35,6225	1,662215249	0	ZNF148	0
MA1654.1	3739	8188	37,95553751	20,47	1,854203103	0	ZNF16	0
MA1124.1	2713	5792	27,54035123	14,48	1,901957958	0	ZNF24	0
MA0528.2	5134	11691	52,11653639	29,2275	1,783133569	0	ZNF263	0
MA1592.1	2509	5280	25,46949548	13,2	1,929507234	0	ZNF274	0
MA1630.1	4217	9170	42,80783677	22,925	1,867299314	0	Znf281	0
MA1593.1	3547	8039	36,0064968	20,0975	1,791590835	0	ZNF317	0
MA1655.1	2965	6554	30,09846716	16,385	1,836952527	0	ZNF341	0
MA0130.1	5460	13285	55,42584509	33,2125	1,668824843	0	ZNF354C	0
MA1594.1	2399	4923	24,35285758	12,3075	1,978700595	0	ZNF382	0
MA1125.1	6080	14992	61,71962237	37,48	1,646734855	0	ZNF384	0
MA1656.1	3297	7151	33,46868338	17,8775	1,872112062	0	ZNF449	0
MA1596.1	5681	13238	57,66927216	33,095	1,742537306	0	ZNF460	0
MA1597.1	3430	7692	34,81880012	19,23	1,810650032	0	ZNF528	0
MA1599.1	3156	6531	32,03735661	16,3275	1,962171589	0	ZNF682	0
MA1600.1	3072	6579	31,1846513	16,4475	1,896011631	0	ZNF684	0
MA0753.2	5160	12009	52,38046899	30,0225	1,744707103	0	ZNF740	0
MA1601.1	3750	8334	38,0672013	20,835	1,827079496	0	ZNF75D	0
MA1155.1	4835	10727	49,08131154	26,8175	1,83019713	0	ZSCAN4	0
MA0115.1	2804	6346	28,46411532	15,865	1,794145308	1,03E-307	NR1H2::RXRA	2,69E-307
MA1573.1	3179	7551	32,27083545	18,8775	1,709486714	2,41E-307	THAP11	6,23E-307
MA0740.1	3053	7154	30,99177748	17,885	1,732836314	1,22E-305	KLF14	3,15E-305
MA0676.1	2552	5579	25,90599939	13,9475	1,857393755	2,35E-305	Nr2e1	6,04E-305
MA0774.1	2808	6376	28,50472033	15,94	1,788250962	4,34E-305	MEIS2	1,11E-304
MA0619.1	2538	5542	25,76388184	13,855	1,859536762	2,07E-304	LIN54	5,29E-304
MA1632.1	2345	4960	23,80468988	12,4	1,919733055	3,16E-304	ATF2	8,04E-304
MA0495.3	2723	6124	27,64186377	15,31	1,805477712	7,57E-303	MAFF	1,92E-302
MA0797.1	2292	4812	23,26667343	12,03	1,934054317	1,55E-302	TGIF2	3,93E-302
MA1135.1	2413	5180	24,49497513	12,95	1,891503871	1,69E-301	FOSB::JUNB	4,25E-301
MA0739.1	2222	4615	22,55608568	11,5375	1,955023677	8,49E-301	Hic1	2,13E-300
MA1485.1	2310	4877	23,449396	12,1925	1,923263974	1,59E-300	FERD3L	3,99E-300
MA1548.1	1808	3437	18,35346665	8,5925	2,135986809	1,75E-300	PLAGL2	4,37E-300
MA0164.1	2412	5186	24,48482388	12,965	1,888532501	5,48E-300	Nr2e3	1,36E-299
MA0813.1	2122	4329	21,54096031	10,8225	1,990386723	1,13E-299	TFAP2B(var.3)	2,80E-299
MA0785.1	2393	5131	24,29195006	12,8275	1,893740016	1,33E-299	POU2F1	3,29E-299
MA0526.3	2427	5235	24,63709268	13,0875	1,882490367	2,24E-299	USF2	5,51E-299

MA0683.1	2351	5007	23,8655974	12,5175	1,906578582	3,14E-299	POU4F2	7,71E-299
MA1119.1	2667	5975	27,07339356	14,9375	1,812444757	6,71E-299	SIX2	1,64E-298
MA1120.1	2163	4457	21,95716171	11,1425	1,970577672	4,40E-298	SOX13	1,07E-297
MA1105.2	2583	5721	26,22068825	14,3025	1,833294057	6,78E-298	GRHL2	1,65E-297
MA0730.1	2533	5572	25,71312557	13,93	1,845881233	4,09E-297	RARA(var.2)	9,90E-297
MA0116.1	2375	5096	24,10922749	12,74	1,892404042	2,27E-296	Znf423	5,48E-296
MA0494.1	2546	5617	25,84509187	14,0425	1,840490786	2,59E-296	Nr1h3::Rxra	6,24E-296
MA1142.1	2541	5606	25,7943356	14,015	1,840480599	1,28E-295	FOSL1::JUND	3,08E-295
MA0090.3	2493	5462	25,30707542	13,655	1,853319328	4,25E-295	TEAD1	1,02E-294
MA0066.1	2343	5017	23,78438737	12,5425	1,896303558	1,83E-293	PPARG	4,36E-293
MA0696.1	2512	5531	25,49994924	13,8275	1,844147477	2,24E-293	ZIC1	5,32E-293
MA0712.2	2657	5982	26,97188103	14,955	1,80353601	4,61E-293	OTX2	1,09E-292
MA0869.1	2482	5443	25,19541163	13,6075	1,851582703	9,57E-293	Sox11	2,26E-292
MA0482.2	2343	5022	23,78438737	12,555	1,894415561	1,27E-292	GATA4	2,99E-292
MA1584.1	2347	5038	23,82499239	12,595	1,891623056	5,87E-292	ZIC5	1,38E-291
MA0107.1	2442	5339	24,78936149	13,3475	1,857228806	8,14E-290	RELA	1,90E-289
MA0143.4	2115	4363	21,46990153	10,9075	1,96836136	1,16E-289	SOX2	2,71E-289
MA0650.2	2228	4702	22,6169932	11,755	1,924031748	1,43E-288	HOXA13	3,32E-288
MA0490.2	2428	5311	24,64724393	13,2775	1,856316621	1,90E-287	JUNB	4,41E-287
MA0792.1	2336	5033	23,7133286	12,5825	1,884627745	2,89E-287	POU5F1B	6,66E-287
MA1494.1	2393	5206	24,29195006	13,015	1,866457937	3,41E-287	HNF4A(var.2)	7,85E-287
MA0111.1	2400	5235	24,36300883	13,0875	1,861547953	6,07E-286	Spz1	1,39E-285
MA1640.1	2441	5361	24,77921023	13,4025	1,848849859	8,43E-286	MEIS2(var.2)	1,93E-285
MA1525.1	2735	6278	27,76367881	15,695	1,768950546	1,67E-285	NFATC4	3,81E-285
MA0744.2	2440	5367	24,76905898	13,4175	1,846026382	2,33E-284	SCRT2	5,30E-284
MA0488.1	2258	4818	22,92153081	12,045	1,90299135	5,01E-284	JUN	1,14E-283
MA0665.1	2564	5763	26,02781444	14,4075	1,806546204	2,64E-282	MSC	5,97E-282
MA0484.2	2759	6377	28,0073089	15,9425	1,756770199	3,39E-282	HNF4G	7,65E-282
MA0092.1	2315	5003	23,50015227	12,5075	1,878884851	9,45E-282	Hand1::Tcf3	2,12E-281
MA0731.1	2588	5847	26,27144452	14,6175	1,797259759	6,46E-281	BCL6B	1,45E-280
MA0815.1	1863	3686	18,91178561	9,215	2,052282757	1,07E-280	TFAP2C(var.3)	2,39E-280
MA1603.1	2361	5157	23,96710994	12,8925	1,858996311	2,20E-279	Dmrt1	4,90E-279
MA0796.1	2272	4888	23,06364836	12,22	1,887368933	2,45E-279	TGIF1	5,44E-279
MA0872.1	1916	3848	19,44980205	9,62	2,021808945	1,77E-278	TFAP2A(var.3)	3,91E-278
MA1639.1	1970	4003	19,99796975	10,0075	1,998298251	1,80E-278	MEIS1(var.2)	3,97E-278
MA0114.4	2755	6395	27,96670389	15,9875	1,749285622	8,14E-278	HNF4A	1,79E-277
MA1134.1	2337	5105	23,72347985	12,7625	1,858842691	5,22E-276	FOS::JUNB	1,15E-275
MA1149.1	2442	5431	24,78936149	13,5775	1,82576774	2,71E-275	RARA::RXRG	5,93E-275

MA1138.1	2287	4959	23,21591717	12,3975	1,87262893	4,22E-275	FOSL2::JUNB	9,22E-275
MA0771.1	2212	4735	22,45457314	11,8375	1,896901638	8,01E-275	HSF4	1,74E-274
MA0762.1	2622	6000	26,61658715	15	1,774439143	4,70E-274	ETV2	1,02E-273
MA1528.1	2230	4794	22,63729571	11,985	1,888802312	5,49E-274	NFIX(var.2)	1,19E-273
MA0601.1	2222	4772	22,55608568	11,93	1,890702907	1,19E-273	Arid3b	2,56E-273
MA1588.1	2246	4849	22,79971576	12,1225	1,880776718	7,49E-273	ZNF136	1,61E-272
MA1645.1	2595	5924	26,3425033	14,81	1,778697049	9,39E-273	NKX2-2	2,01E-272
MA0614.1	2151	4567	21,83534667	11,4175	1,912445515	1,90E-272	Foxj2	4,06E-272
MA0029.1	2498	5636	25,35783169	14,09	1,799704166	1,73E-270	Mecom	3,69E-270
MA1104.2	2323	5099	23,5813623	12,7475	1,849881333	3,65E-270	GATA6	7,76E-270
MA0655.1	2103	4439	21,34808649	11,0975	1,923684297	5,65E-270	JDP2	1,20E-269
MA1589.1	2219	4789	22,52563192	11,9725	1,881447644	2,37E-269	ZNF140	5,00E-269
MA1420.1	1994	4124	20,24159984	10,31	1,963297753	4,78E-269	IRF5	1,01E-268
MA0112.3	1929	3938	19,58176835	9,845	1,989006435	1,42E-268	ESR1	2,99E-268
MA0491.2	2322	5107	23,57121104	12,7675	1,846188451	2,04E-268	JUND	4,28E-268
MA0036.3	2176	4667	22,08912801	11,6675	1,893218599	3,53E-268	GATA2	7,37E-268
MA0743.2	2286	5001	23,20576591	12,5025	1,856090055	7,73E-268	SCRT1	1,61E-267
MA0860.1	2548	5811	25,86539438	14,5275	1,780443598	1,05E-267	Rarg(var.2)	2,19E-267
MA1153.1	2284	5000	23,1854634	12,5	1,854837072	4,99E-267	Smad4	1,03E-266
MA0673.1	2282	4994	23,1651609	12,485	1,855439399	5,14E-267	NKX2-8	1,06E-266
MA1657.1	2141	4573	21,73383413	11,4325	1,901056998	2,12E-266	ZNF652	4,36E-266
MA0135.1	2140	4572	21,72368287	11,43	1,900584678	4,55E-266	Lhx3	9,36E-266
MA0492.1	2064	4353	20,9521876	10,8825	1,92531014	6,43E-265	JUND(var.2)	1,32E-264
MA0809.2	2394	5352	24,30210131	13,38	1,816300546	1,07E-264	TEAD4	2,20E-264
MA0078.1	2004	4180	20,34311237	10,45	1,946709318	2,76E-264	Sox17	5,62E-264
MA1471.1	2030	4258	20,60704497	10,645	1,935842646	5,31E-264	BARX2	1,08E-263
MA0624.1	2575	5934	26,13947823	14,835	1,762014036	3,35E-262	NFATC1	6,79E-262
MA1154.1	2311	5117	23,45954725	12,7925	1,833851652	1,38E-261	ZNF282	2,79E-261
MA1121.1	2191	4758	22,24139681	11,895	1,869810577	1,18E-260	TEAD2	2,39E-260
MA0486.2	2201	4789	22,34290935	11,9725	1,866185788	1,59E-260	HSF1	3,20E-260
MA0100.3	2105	4502	21,368389	11,255	1,898568547	2,64E-260	MYB	5,30E-260
MA0625.1	2600	6030	26,39325957	15,075	1,750796655	8,41E-260	NFATC3	1,68E-259
MA0820.1	2518	5773	25,56085677	14,4325	1,771062308	1,52E-259	FIGLA	3,03E-259
MA1535.1	2055	4364	20,86082631	10,91	1,912083072	1,73E-258	NR2C1	3,43E-258
MA1144.1	2354	5270	23,89605116	13,175	1,813742024	2,13E-258	FOSL2::JUND	4,23E-258
MA0074.1	1859	3793	18,87118059	9,4825	1,990106047	4,42E-258	RXRA::VDR	8,74E-258
MA0751.1	2268	5015	23,02304335	12,5375	1,836334464	5,40E-257	ZIC4	1,07E-256
MA0858.1	2369	5335	24,04831997	13,3375	1,803060541	1,35E-255	Rarb(var.2)	2,65E-255

MA0122.3	2028	4304	20,58674246	10,76	1,913266028	4,33E-255	Nkx3-2	8,51E-255
MA0913.2	2026	4305	20,56643996	10,7625	1,910935187	6,44E-254	HOXD9	1,26E-253
MA0119.1	2270	5048	23,04334585	12,62	1,825938657	7,57E-253	NFIC::TLX1	1,48E-252
MA1527.1	2299	5138	23,33773221	12,845	1,816872885	1,04E-252	NFIC(var.2)	2,02E-252
MA0868.2	1998	4230	20,28220485	10,575	1,917938993	1,42E-252	SOX8	2,77E-252
MA0101.1	2184	4788	22,17033804	11,97	1,852158566	1,78E-252	REL	3,45E-252
MA0808.1	2159	4713	21,91655669	11,7825	1,860093927	2,34E-252	TEAD3	4,53E-252
MA1132.1	2247	4982	22,80986702	12,455	1,831382338	3,67E-252	JUN::JUNB	7,08E-252
MA1536.1	2217	4892	22,50532941	12,23	1,840174114	6,58E-252	NR2C2(var.2)	1,27E-251
MA1650.1	1776	3590	18,02862654	8,975	2,008760617	1,53E-251	ZBTB14	2,93E-251
MA1547.1	2361	5344	23,96710994	13,36	1,793945355	1,41E-250	PITX2	2,70E-250
MA0866.1	1976	4177	20,05887727	10,4425	1,920888415	1,58E-250	SOX21	3,02E-250
MA0063.2	2173	4767	22,05867425	11,9175	1,850948122	1,60E-250	NKX2-5	3,05E-250
MA0736.1	1745	3508	17,71393767	8,77	2,019833258	2,48E-250	GLIS2	4,71E-250
MA0046.2	2187	4817	22,2007918	12,0425	1,84353679	2,36E-249	HNF1A	4,47E-249
MA0479.1	2173	4775	22,05867425	11,9375	1,847847057	2,90E-249	FOXH1	5,48E-249
MA0091.1	2403	5485	24,39346259	13,7125	1,778921611	4,31E-249	TAL1::TCF3	8,12E-249
MA0660.1	1919	4021	19,48025581	10,0525	1,937851859	1,67E-248	MEF2B	3,14E-248
MA0691.1	2124	4633	21,56126282	11,5825	1,861537908	3,03E-248	TFAP4	5,68E-248
MA0098.3	2398	5491	24,34270632	13,7275	1,773280373	5,69E-246	ETS1	1,06E-245
MA0467.1	1932	4074	19,61222211	10,185	1,925598636	5,92E-246	Crx	1,10E-245
MA0760.1	2372	5410	24,07877373	13,525	1,780315987	6,20E-246	ERF	1,15E-245
MA0714.1	2368	5402	24,03816871	13,505	1,779945851	2,76E-245	PITX3	5,12E-245
MA1532.1	2132	4682	21,64247285	11,705	1,848993836	2,51E-244	NR1D2	4,64E-244
MA0602.1	1664	3312	16,89168612	8,28	2,040058711	6,71E-244	Arid5a	1,24E-243
MA0765.2	2503	5840	25,40858796	14,6	1,740314244	2,04E-243	ETV5	3,76E-243
MA1531.1	2125	4680	21,57141407	11,7	1,843710604	2,56E-241	NR1D1	4,70E-241
MA0671.1	1899	4010	19,27723074	10,025	1,922915784	2,75E-240	NFIX	5,04E-240
MA0672.1	2050	4466	20,81007004	11,165	1,863866551	2,76E-239	NKX2-3	5,05E-239
MA0038.2	2056	4485	20,87097757	11,2125	1,861402681	3,86E-239	GFI1	7,04E-239
MA0474.2	2317	5289	23,52045478	13,2225	1,778820554	1,24E-238	ERG	2,27E-238
MA0140.2	2026	4404	20,56643996	11,01	1,867978198	1,19E-237	GATA1::TAL1	2,15E-237
MA0775.1	2179	4875	22,11958177	12,1875	1,814940043	1,02E-236	MEIS3	1,84E-236
MA1118.1	2084	4589	21,15521267	11,4725	1,843993259	4,15E-236	SIX1	7,49E-236
MA1534.1	1985	4291	20,15023855	10,7275	1,878372273	4,84E-236	NR1I3	8,72E-236
MA0682.2	2332	5354	23,67272358	13,385	1,76860094	4,94E-236	PITX1	8,88E-236
MA0832.1	2120	4700	21,5206578	11,75	1,831545345	7,03E-236	Tcf21	1,26E-235
MA0670.1	1926	4117	19,55131459	10,2925	1,899569064	9,96E-236	NFIA	1,78E-235

Supplementary Table 2

MA0719.1	2350	5414	23,85544615	13,535	1,762500639	1,60E-235	RHOXF1	2,86E-235
MA0633.1	1832	3842	18,59709674	9,605	1,936189145	2,34E-235	Twist2	4,16E-235
MA0789.1	2143	4775	21,75413664	11,9375	1,822336053	3,71E-235	POU3F4	6,58E-235
MA0831.2	1944	4178	19,73403715	10,445	1,889328593	1,68E-234	TFE3	2,98E-234
MA0897.1	1973	4275	20,02842351	10,6875	1,874004539	7,94E-233	Hmx2	1,40E-232
MA0667.1	1978	4293	20,07917978	10,7325	1,87087629	2,41E-232	MYF6	4,25E-232
MA0070.1	1710	3507	17,35864379	8,7675	1,979885234	5,90E-232	PBX1	1,04E-231
MA0059.1	2033	4470	20,63749873	11,175	1,846756039	1,42E-230	MAX::MYC	2,49E-230
MA0841.1	2019	4432	20,49538118	11,08	1,849763644	6,80E-230	NFE2	1,19E-229
MA0766.2	1773	3702	17,99817277	9,255	1,94469722	9,51E-230	GATA5	1,66E-229
MA1479.1	1759	3670	17,85605522	9,175	1,946164057	3,23E-228	DMRTC2	5,63E-228
MA0117.2	2191	4972	22,24139681	12,43	1,789332004	7,42E-228	Mafb	1,29E-227
MA0863.1	1615	3258	16,39427469	8,145	2,012802295	9,21E-228	MTF1	1,60E-227
MA0715.1	1742	3626	17,68348391	9,065	1,950742847	2,92E-227	PROP1	5,06E-227
MA1101.2	1944	4223	19,73403715	10,5575	1,869196036	3,12E-227	BACH2	5,39E-227
MA1473.1	1911	4127	19,39904578	10,3175	1,880207975	8,68E-227	CDX4	1,50E-226
MA0898.1	2042	4523	20,72886001	11,3075	1,833195668	1,30E-226	Hmx3	2,24E-226
MA1563.1	1669	3420	16,94244239	8,55	1,98157221	2,33E-226	SOX18	4,00E-226
MA0878.2	1852	3956	18,80012182	9,89	1,900922327	4,70E-226	CDX1	8,05E-226
MA1530.1	1630	3310	16,5465435	8,275	1,999582296	4,84E-226	NKX6-3	8,26E-226
MA0842.2	1910	4131	19,38889453	10,3275	1,877404457	1,16E-225	NRL	1,98E-225
MA1484.1	2222	5107	22,55608568	12,7675	1,766679904	2,48E-222	ETS2	4,21E-222
MA1580.1	1835	3930	18,6275505	9,825	1,895933893	4,89E-222	ZBTB32	8,29E-222
MA1636.1	1689	3505	17,14546747	8,7625	1,956686729	1,69E-221	CEBPG(var.2)	2,86E-221
MA0641.1	2368	5576	24,03816871	13,94	1,724402347	4,51E-221	ELF4	7,61E-221
MA0506.1	1810	3863	18,37376916	9,6575	1,902538872	7,07E-221	NRF1	1,19E-220
MA0707.1	1915	4179	19,4396508	10,4475	1,860698808	2,09E-220	MNX1	3,51E-220
MA1533.1	1631	3345	16,55669475	8,3625	1,979873812	3,49E-220	NR1I2	5,84E-220
MA0084.1	1931	4235	19,60207086	10,5875	1,851435264	3,52E-219	SRY	5,89E-219
MA0778.1	1578	3212	16,01867831	8,03	1,994854086	9,14E-217	NFKB2	1,53E-216
MA1117.1	1807	3881	18,3433154	9,7025	1,890576181	1,82E-216	RELB	3,03E-216
MA0737.1	1694	3550	17,19622373	8,875	1,937602674	3,30E-216	GLIS3	5,48E-216
MA0510.2	1624	3352	16,48563598	8,38	1,967259663	2,49E-215	RFX5	4,13E-215
MA0752.1	1584	3238	16,07958583	8,095	1,986360201	3,72E-215	ZNF410	6,15E-215
MA0657.1	1958	4343	19,87615471	10,8575	1,830638241	3,86E-215	KLF13	6,37E-215
MA0105.4	1515	3052	15,37914932	7,63	2,015615901	3,27E-213	NFKB1	5,38E-213
MA0833.2	1725	3668	17,5109126	9,17	1,909586979	1,27E-211	ATF4	2,08E-211
MA1478.1	1640	3426	16,64805603	8,565	1,943731002	1,73E-210	DMRTA2	2,83E-210

Supplementary Table 2

MA0025.2	1680	3547	17,05410618	8,8675	1,923214681	9,30E-210	NFIL3		1,52E-209
MA0037.3	1642	3438	16,66835854	8,595	1,939308731	2,00E-209	GATA3		3,27E-209
MA1110.1	1744	3738	17,70378642	9,345	1,894466176	2,46E-209	NR1H4		4,00E-209
MA0799.1	1724	3685	17,50076134	9,2125	1,899675587	2,48E-208	RFX4		4,03E-208
MA1549.1	1796	3902	18,23165161	9,755	1,868954547	7,75E-208	POU6F1(var.2)		1,26E-207
MA0077.1	1668	3525	16,93229114	8,8125	1,921394739	1,52E-207	SOX9		2,46E-207
MA1542.1	1856	4086	18,84072683	10,215	1,844417702	3,09E-207	OSR1		4,99E-207
MA0692.1	1668	3542	16,93229114	8,855	1,912172912	1,05E-204	TFEB		1,70E-204
MA0600.2	1522	3120	15,4502081	7,8	1,98079591	2,07E-204	RFX2		3,33E-204
MA1151.1	2124	4933	21,56126282	12,3325	1,748328629	5,88E-204	RORC		9,43E-204
MA0656.1	1367	2685	13,87676378	6,7125	2,067301867	5,92E-204	JDP2(var.2)		9,47E-204
MA0884.1	1524	3130	15,47051061	7,825	1,977062059	1,20E-203	DUXA		1,92E-203
MA0124.2	1708	3668	17,33834129	9,17	1,890767861	2,18E-203	Nkx3-1		3,47E-203
MA1521.1	1839	4061	18,66815552	10,1525	1,838774244	3,29E-203	MAFA		5,23E-203
MA0472.2	1675	3576	17,00334991	8,94	1,901940706	1,72E-202	EGR2		2,73E-202
MA1489.1	1757	3833	17,83575272	9,5825	1,861283873	2,41E-200	FOXN3		3,81E-200
MA1493.1	1369	2710	13,89706629	6,775	2,051227496	3,69E-200	HES6		5,83E-200
MA0147.3	1576	3302	15,9983758	8,255	1,938022507	7,81E-200	MYC		1,23E-199
MA1126.1	1443	2924	14,64825906	7,31	2,003865808	4,31E-199	FOS::JUN(var.2)		6,78E-199
MA0607.1	1568	3288	15,91716577	8,22	1,936394863	2,97E-198	Bhlha15		4,67E-198
MA0626.1	1461	2981	14,83098163	7,4525	1,990067981	4,89E-198	Npas2		7,66E-198
MA1556.1	1610	3412	16,34351842	8,53	1,916004505	5,09E-198	RXRG(var.2)		7,96E-198
MA1571.1	1682	3627	17,07440869	9,0675	1,883033768	1,41E-197	TGIF2LX		2,19E-197
MA1131.1	1492	3077	15,14567049	7,6925	1,968887942	9,68E-197	FOSL2::JUN(var.2)		1,51E-196
MA0043.3	1487	3065	15,09491422	7,6625	1,969972492	2,53E-196	HLF		3,94E-196
MA1146.1	1439	2938	14,60765405	7,345	1,988788842	1,81E-194	NR1H4::RXRA		2,80E-194
MA1150.1	2105	4953	21,368389	12,3825	1,72569263	2,94E-193	RORB		4,56E-193
MA0713.1	1606	3430	16,30291341	8,575	1,901214392	4,10E-193	PHOX2A		6,33E-193
MA1572.1	1591	3390	16,1506446	8,475	1,905680779	1,94E-192	TGIF2LY		2,99E-192
MA0865.1	1707	3735	17,32819003	9,3375	1,855763323	2,64E-192	E2F8		4,07E-192
MA1538.1	1490	3100	15,12536798	7,75	1,951660385	1,04E-191	NR2F1(var.3)		1,59E-191
MA0502.2	2007	4662	20,37356614	11,655	1,748053722	4,88E-191	NFYB		7,47E-191
MA0861.1	1481	3079	15,0340067	7,6975	1,953102527	7,57E-191	TP73		1,16E-190
MA1145.1	1442	2967	14,63810781	7,4175	1,97345572	8,14E-191	FOSL2::JUND(var.2)		1,24E-190
MA0756.1	1482	3088	15,04415795	7,72	1,948725124	8,61E-190	ONECUT2		1,31E-189
MA0475.2	1955	4510	19,84570094	11,275	1,76015086	1,13E-189	FLI1		1,72E-189
MA0695.1	1550	3289	15,7344432	8,2225	1,91358385	2,91E-189	ZBTB7C		4,42E-189
MA0072.1	2110	5000	21,41914526	12,5	1,713531621	3,54E-189	RORA(var.2)		5,35E-189

Supplementary Table 2

MA0632.2	1316	2620	13,35904984	6,55	2,039549594	5,37E-189	TCFL5	8,11E-189
MA0102.4	1619	3497	16,43487971	8,7425	1,879883295	1,67E-188	CEBPA	2,52E-188
MA0153.2	1449	3001	14,70916658	7,5025	1,960568688	2,26E-188	HNF1B	3,41E-188
MA0605.2	1273	2505	12,92254593	6,2625	2,063480389	4,57E-188	ATF3	6,86E-188
MA0892.1	1572	3365	15,95777078	8,4125	1,896911832	2,14E-187	GSX1	3,21E-187
MA0035.4	1640	3567	16,64805603	8,9175	1,866897228	2,63E-187	GATA1	3,94E-187
MA0106.3	1491	3135	15,13551924	7,8375	1,931166729	3,16E-186	TP53	4,72E-186
MA0009.2	1350	2738	13,70419247	6,845	2,002073407	7,59E-185	TBXT	1,13E-184
MA0870.1	1512	3212	15,34869556	8,03	1,911419124	1,32E-183	Sox1	1,97E-183
MA1539.1	1424	2961	14,45538524	7,4025	1,952770718	8,21E-183	NR2F6(var.3)	1,22E-182
MA1127.1	1301	2611	13,20678104	6,5275	2,023252553	9,34E-183	FOSB::JUN	1,38E-182
MA0468.1	1660	3664	16,85108111	9,16	1,839637676	1,37E-181	DUX4	2,02E-181
MA0621.1	1677	3719	17,02365242	9,2975	1,830992463	5,12E-181	mix-a	7,55E-181
MA0836.2	1544	3326	15,67353568	8,315	1,884971218	2,21E-180	CEBPD	3,26E-180
MA1147.1	1353	2774	13,73464623	6,935	1,980482513	6,83E-180	NR4A2::RXRA	1,00E-179
MA1520.1	1756	3969	17,82560146	9,9225	1,796482889	1,57E-179	MAF	2,29E-179
MA1570.1	1601	3508	16,25215714	8,77	1,853153608	2,07E-178	TFAP4(var.2)	3,03E-178
MA1644.1	1897	4416	19,25692823	11,04	1,744286977	6,39E-178	NFYC	9,33E-178
MA0703.2	1715	3858	17,40940006	9,645	1,80501815	1,65E-177	LMX1B	2,40E-177
MA0829.2	1539	3335	15,62277941	8,3375	1,873796631	1,30E-176	SREBF1(var.2)	1,89E-176
MA0031.1	1465	3119	14,87158664	7,7975	1,907224962	2,78E-176	FOXD1	4,02E-176
MA0613.1	1465	3119	14,87158664	7,7975	1,907224962	2,78E-176	FOXG1	4,02E-176
MA0157.2	1465	3119	14,87158664	7,7975	1,907224962	2,78E-176	FOXO3	4,02E-176
MA0793.1	1544	3354	15,67353568	8,385	1,869235025	5,85E-176	POU6F2	8,44E-176
MA0631.1	1256	2521	12,74997462	6,3025	2,023002717	5,95E-176	Six3	8,57E-176
MA0903.1	1486	3189	15,08476297	7,9725	1,892099463	6,26E-175	HOXB3	8,99E-175
MA0109.1	1522	3301	15,4502081	8,2525	1,872185168	4,90E-174	HLTF	7,03E-174
MA0804.1	1260	2548	12,79057964	6,37	2,007940288	4,82E-173	TBX19	6,90E-173
MA1602.1	1332	2761	13,5214699	6,9025	1,958923564	1,29E-171	ZSCAN29	1,84E-171
MA0609.2	1237	2495	12,5571008	6,2375	2,013162453	7,21E-171	CREM	1,03E-170
MA0700.2	1399	2960	14,2016039	7,4	1,919135662	9,88E-171	LHX2	1,41E-170
MA1483.1	1960	4674	19,89645721	11,685	1,702734892	4,11E-170	ELF2	5,84E-170
MA0058.3	1346	2812	13,66358745	7,03	1,943611302	8,84E-170	MAX	1,25E-169
MA0706.1	1465	3162	14,87158664	7,905	1,881288633	2,29E-169	MEOX2	3,24E-169
MA1102.2	3185	8800	32,33174297	22	1,46962468	7,67E-169	CTCFL	1,08E-168
MA0727.1	1349	2827	13,69404121	7,0675	1,937607529	1,05E-168	NR3C2	1,48E-168
MA1122.1	1409	3002	14,30311643	7,505	1,90581165	1,25E-168	TFDP1	1,76E-168
MA0007.3	1477	3204	14,99340169	8,01	1,871835416	2,42E-168	Ar	3,40E-168

Supplementary Table 2

MA0622.1	1311	2722	13,30829357	6,805	1,955664008	6,22E-168	Mlxip	8,72E-168
MA0069.1	1269	2602	12,88194092	6,505	1,980313746	6,56E-168	PAX6	9,18E-168
MA1099.2	1159	2299	11,76530301	5,7475	2,047029668	1,10E-166	HES1	1,54E-166
MA0839.1	1131	2235	11,48106791	5,5875	2,054777255	4,76E-164	CREB3L1	6,63E-164
MA0610.1	1467	3204	14,89188915	8,01	1,859162191	9,65E-164	DMRT3	1,34E-163
MA0694.1	1354	2872	13,74479748	7,18	1,914317198	1,25E-163	ZBTB7B	1,74E-163
MA0654.1	1344	2844	13,64328495	7,11	1,918886772	1,90E-163	ISX	2,64E-163
MA1468.1	1414	3049	14,3538727	7,6225	1,883092516	1,92E-163	ATOH7	2,66E-163
MA0699.1	1292	2694	13,11541975	6,735	1,947352599	2,31E-163	LBX2	3,18E-163
MA1524.1	1408	3033	14,29296518	7,5825	1,884993759	3,54E-163	MSGN1	4,88E-163
MA0108.2	1572	3523	15,95777078	8,8075	1,811838863	3,70E-163	TBP	5,09E-163
MA0718.1	1223	2500	12,41498325	6,25	1,98639732	8,64E-163	RAX	1,19E-162
MA0060.3	1825	4313	18,52603797	10,7825	1,718157938	7,11E-162	NFYA	9,75E-162
MA1555.1	1317	2776	13,3692011	6,94	1,926397852	1,01E-161	RXRB(var.2)	1,39E-161
MA1133.1	1199	2449	12,17135316	6,1225	1,987971117	9,70E-160	JUN::JUNB(var.2)	1,33E-159
MA0104.4	1162	2346	11,79575678	5,865	2,011211727	1,82E-159	MYCN	2,48E-159
MA0907.1	1283	2694	13,02405847	6,735	1,933787449	5,66E-159	HOXC13	7,71E-159
MA0646.1	1250	2599	12,6890671	6,4975	1,95291529	5,84E-159	GCM1	7,93E-159
MA0871.2	1404	3049	14,25236017	7,6225	1,86977503	8,17E-159	TFEC	1,11E-158
MA1562.1	1328	2826	13,48086489	7,065	1,908119588	9,28E-159	SOX14	1,26E-158
MA1553.1	1264	2642	12,83118465	6,605	1,942647184	1,70E-158	RARG(var.3)	2,30E-158
MA0822.1	1122	2241	11,38970663	5,6025	2,032968608	3,74E-158	HES7	5,04E-158
MA0634.1	1493	3328	15,15582174	8,32	1,82161319	8,12E-157	ALX3	1,09E-156
MA1504.1	1135	2285	11,52167293	5,7125	2,016923051	9,72E-157	HOXC4	1,31E-156
MA1608.1	1280	2703	12,99360471	6,7575	1,922841984	4,99E-156	Isl1	6,69E-156
MA0620.3	1362	2944	13,82600751	7,36	1,878533629	7,70E-156	MITF	1,03E-155
MA0905.1	1243	2602	12,61800832	6,505	1,939739942	5,00E-155	HOXC10	6,69E-155
MA0156.2	1714	4021	17,39924881	10,0525	1,730837981	1,01E-154	FEV	1,35E-154
MA0611.1	1479	3301	15,01370419	8,2525	1,819291632	1,34E-154	Dux	1,78E-154
MA0759.1	1792	4272	18,19104659	10,68	1,703281516	6,62E-154	ELK3	8,80E-154
MA1143.1	993	1915	10,0801949	4,7875	2,10552374	2,17E-152	FOSL1::JUND(var.2)	2,89E-152
MA1585.1	1539	3501	15,62277941	8,7525	1,784950519	4,98E-152	ZKSCAN1	6,60E-152
MA0896.1	1478	3319	15,00355294	8,2975	1,80820162	1,46E-151	Hmx1	1,94E-151
MA1499.1	1060	2104	10,7603289	5,26	2,045689905	1,93E-151	HOXB4	2,54E-151
MA1507.1	1060	2104	10,7603289	5,26	2,045689905	1,93E-151	HOXD4	2,54E-151
MA0628.1	1008	1968	10,23246371	4,92	2,079769047	5,47E-150	POU6F1	7,19E-150
MA1463.1	1385	3055	14,05948635	7,6375	1,840849276	2,70E-149	ARGFX	3,54E-149
MA0662.1	1344	2942	13,64328495	7,355	1,854967362	6,28E-148	MIXL1	8,24E-148

Supplementary Table 2

MA0113.3	1313	2851	13,32859608	7,1275	1,870024003	8,31E-148	NR3C1	1,09E-147
MA1575.1	1329	2899	13,49101614	7,2475	1,861471699	1,06E-147	THRB(var.2)	1,38E-147
MA0914.1	1486	3376	15,08476297	8,44	1,787294191	6,23E-147	ISL2	8,12E-147
MA0014.3	1084	2197	11,00395899	5,4925	2,003451796	1,22E-146	PAX5	1,58E-146
MA0723.1	1327	2903	13,47071363	7,2575	1,856109354	3,46E-146	VAX2	4,50E-146
MA0821.1	1121	2306	11,37955538	5,765	1,973903794	7,12E-146	HES5	9,24E-146
MA0840.1	1105	2262	11,21713532	5,655	1,983578305	1,26E-145	Creb5	1,64E-145
MA0648.1	1093	2229	11,09532027	5,5725	1,99108484	1,87E-145	GSC	2,42E-145
MA0880.1	1174	2463	11,91757182	6,1575	1,935456244	5,64E-145	Dlx3	7,28E-145
MA0819.1	998	1968	10,13095117	4,92	2,059136417	1,24E-144	CLOCK	1,59E-144
MA0716.1	1175	2470	11,92772307	6,175	1,931615073	2,74E-144	PRRX1	3,51E-144
MA0717.1	1175	2470	11,92772307	6,175	1,931615073	2,74E-144	RAX2	3,51E-144
MA0630.1	1175	2470	11,92772307	6,175	1,931615073	2,74E-144	SHOX	3,51E-144
MA0720.1	1175	2470	11,92772307	6,175	1,931615073	2,74E-144	Shox2	3,51E-144
MA1139.1	1015	2018	10,30352249	5,045	2,042323585	4,49E-144	FOSL2::JUNB(var.2)	5,73E-144
MA1140.2	1007	1996	10,22231246	4,99	2,04855961	4,86E-144	JUNB(var.2)	6,20E-144
MA0825.1	1265	2738	12,8413359	6,845	1,876016933	2,21E-143	MNT	2,81E-143
MA1552.1	1249	2699	12,67891585	6,7475	1,879053849	3,99E-142	RARB(var.3)	5,07E-142
MA0890.1	1247	2698	12,65861334	6,745	1,876740302	2,30E-141	GBX2	2,92E-141
MA1136.1	1047	2125	10,6283626	5,3125	2,000632961	7,65E-141	FOSB::JUNB(var.2)	9,69E-141
MA0823.1	1118	2327	11,34910161	5,8175	1,950855456	8,87E-141	HEY1	1,12E-140
MA0876.1	1174	2489	11,91757182	6,2225	1,915238541	1,10E-140	BSX	1,39E-140
MA0666.1	1174	2489	11,91757182	6,2225	1,915238541	1,10E-140	MSX1	1,39E-140
MA0708.1	1174	2489	11,91757182	6,2225	1,915238541	1,10E-140	MSX2	1,39E-140
MA0629.1	1413	3201	14,34372145	8,0025	1,792405055	2,24E-140	Rhox11	2,81E-140
MA0854.1	1176	2505	11,93787433	6,2625	1,906247398	4,94E-139	Alx1	6,19E-139
MA0608.1	1285	2826	13,04436098	7,065	1,846335595	5,53E-139	Creb3l2	6,93E-139
MA0781.1	981	1952	9,95837986	4,88	2,040651611	9,94E-139	PAX9	1,24E-138
MA0891.1	1136	2397	11,53182418	5,9925	1,924376167	9,85E-138	GSC2	1,23E-137
MA0763.1	1576	3735	15,9983758	9,3375	1,713346806	3,45E-136	ETV3	4,29E-136
MA0834.1	992	2002	10,07004365	5,005	2,011996733	3,74E-135	ATF7	4,65E-135
MA1506.1	1000	2028	10,15125368	5,07	2,002219661	1,64E-134	HOXD10	2,04E-134
MA0644.1	1173	2529	11,90742057	6,3225	1,88334054	7,82E-134	ESX1	9,70E-134
MA1470.1	991	2008	10,0598924	5,02	2,003962629	1,47E-133	BACH2(var.2)	1,82E-133
MA0131.2	816	1533	8,283423003	3,8325	2,161362819	1,01E-132	HINFP	1,24E-132
MA0019.1	1234	2718	12,52664704	6,795	1,843509498	1,80E-132	Ddit3::Cebpa	2,22E-132
MA0879.1	1032	2132	10,4760938	5,33	1,965496022	3,92E-132	Dlx1	4,83E-132
MA1474.1	1071	2245	10,87199269	5,6125	1,937103375	6,96E-132	CREB3L4	8,56E-132

Supplementary Table 2

MA0616.2	1013	2081	10,28321998	5,2025	1,976592019	1,28E-131	HES2	1,57E-131
MA1129.1	928	1854	9,420363415	4,635	2,032440866	1,57E-129	FOSL1::JUN(var.2)	1,92E-129
MA1554.1	1099	2345	11,15622779	5,8625	1,902981287	9,00E-129	RFX7	1,10E-128
MA0794.1	953	1929	9,674144757	4,8225	2,006043495	1,41E-128	PROX1	1,72E-128
MA0874.1	1088	2323	11,044564	5,8075	1,90177598	3,53E-127	Arx	4,31E-127
MA0758.1	1188	2617	12,05968937	6,5425	1,843284581	3,61E-127	E2F7	4,39E-127
MA1480.1	1000	2075	10,15125368	5,1875	1,956868179	2,77E-126	DPRX	3,37E-126
MA1519.1	1167	2561	11,84651304	6,4025	1,850294892	3,12E-126	LHX5	3,77E-126
MA0674.1	1167	2561	11,84651304	6,4025	1,850294892	3,12E-126	NKX6-1	3,77E-126
MA0675.1	1167	2561	11,84651304	6,4025	1,850294892	3,12E-126	NKX6-2	3,77E-126
MA0885.1	1265	2857	12,8413359	7,1425	1,797876921	8,09E-126	Dlx2	9,76E-126
MA0649.1	976	2012	9,907623592	5,03	1,969706479	2,22E-125	HEY2	2,68E-125
MA0893.2	1110	2404	11,26789158	6,01	1,874857169	1,49E-124	GSX2	1,79E-124
MA0795.1	1042	2207	10,57760633	5,5175	1,917101284	2,20E-124	SMAD3	2,64E-124
MA0669.1	1106	2394	11,22728657	5,985	1,875904189	2,76E-124	NEUROG2	3,31E-124
MA1475.1	815	1571	8,273271749	3,9275	2,106498217	2,79E-124	CREB3L4(var.2)	3,34E-124
MA0779.1	919	1864	9,329002132	4,66	2,001931788	4,06E-123	PAX1	4,85E-123
MA0525.2	1114	2427	11,3084966	6,0675	1,863781887	7,82E-123	TP63	9,34E-123
MA0618.1	1082	2338	10,98365648	5,845	1,879154231	4,55E-122	LBX1	5,42E-122
MA1496.1	900	1817	9,136128312	4,5425	2,011255545	5,34E-122	HOXA4	6,36E-122
MA0704.1	1134	2497	11,51152167	6,2425	1,844056335	3,37E-121	Lhx4	3,99E-121
MA0721.1	1134	2497	11,51152167	6,2425	1,844056335	3,37E-121	UNCX	3,99E-121
MA0709.1	923	1889	9,369607146	4,7225	1,984035394	1,11E-120	Msx3	1,31E-120
MA0661.1	1080	2344	10,96335397	5,86	1,870879518	3,06E-120	MEOX1	3,62E-120
MA1568.1	1385	3269	14,05948635	8,1725	1,720340942	6,06E-120	TCF21(var.2)	7,15E-120
MA0647.1	1197	2695	12,15105065	6,7375	1,803495459	8,63E-120	GRHL1	1,02E-119
MA1466.1	894	1820	9,07522079	4,55	1,99455402	1,91E-118	ATF6	2,25E-118
MA0710.1	1015	2168	10,30352249	5,42	1,901018909	4,36E-118	NOTO	5,12E-118
MA0911.1	893	1824	9,065069536	4,56	1,987953846	3,05E-117	Hoxa11	3,58E-117
MA0883.1	1070	2338	10,86184144	5,845	1,858313334	1,10E-116	Dmbx1	1,29E-116
MA0461.2	1020	2192	10,35427875	5,48	1,889466926	1,35E-116	Atoh1	1,58E-116
MA0702.2	1091	2402	11,07501776	6,005	1,844299378	2,15E-116	LMX1A	2,51E-116
MA0895.1	1117	2482	11,33895036	6,205	1,82738926	5,39E-116	HMBOX1	6,29E-116
MA0853.1	1106	2456	11,22728657	6,14	1,828548301	5,17E-115	Alx4	6,01E-115
MA0881.1	1110	2473	11,26789158	6,1825	1,822546152	2,79E-114	Dlx4	3,24E-114
MA1503.1	955	2019	9,694447264	5,0475	1,920643341	3,84E-114	HOXB9	4,45E-114
MA0083.3	1011	2182	10,26291747	5,455	1,881378088	4,59E-114	SRF	5,31E-114
MA0827.1	1010	2203	10,25276622	5,5075	1,861600766	2,08E-110	OLIG3	2,40E-110

Supplementary Table 2

MA0817.1	974	2100	9,887321084	5,25	1,883299254	4,93E-110	BHLHE23	5,69E-110
MA1464.1	1101	2480	11,1765303	6,2	1,802666178	1,80E-109	ARNT2	2,08E-109
MA0894.1	932	1982	9,46096843	4,955	1,909378089	2,08E-109	HESX1	2,40E-109
MA0748.2	1084	2430	11,00395899	6,075	1,811351274	2,48E-109	YY2	2,85E-109
MA0125.1	874	1821	8,872195716	4,5525	1,948862321	1,66E-108	Nobox	1,91E-108
MA0910.2	1199	2785	12,17135316	6,9625	1,748129718	1,87E-108	HOXD8	2,14E-108
MA0906.1	834	1718	8,466145569	4,295	1,971163113	1,04E-106	HOXC12	1,18E-106
MA0658.1	878	1844	8,912800731	4,61	1,933362415	1,39E-106	LHX6	1,58E-106
MA0908.1	950	2058	9,643690996	5,145	1,874381146	1,05E-105	HOXD11	1,19E-105
MA0027.2	907	1933	9,207187088	4,8325	1,905263753	1,08E-105	EN1	1,23E-105
MA0735.1	998	2203	10,13095117	5,5075	1,839482737	3,78E-105	GLIS1	4,30E-105
MA0767.1	780	1581	7,91797787	3,9525	2,003283459	4,12E-104	GCM2	4,68E-104
MA0651.1	799	1635	8,11085169	4,0875	1,984306224	5,50E-104	HOXC11	6,24E-104
MA0828.1	1033	2316	10,48624505	5,79	1,811095864	6,99E-104	SREBF2(var.2)	7,91E-104
MA0818.1	862	1820	8,750380672	4,55	1,923160587	5,25E-103	BHLHE22	5,94E-103
MA0873.1	823	1713	8,354481778	4,2825	1,950842213	2,93E-102	HOXD12	3,30E-102
MA0888.1	753	1516	7,643894021	3,79	2,016858581	2,93E-102	EVX2	3,31E-102
MA0887.1	768	1560	7,796162826	3,9	1,999016109	7,11E-102	EVX1	8,00E-102
MA0638.1	811	1684	8,232666734	4,21	1,955502787	2,12E-101	CREB3	2,38E-101
MA0603.1	1137	2652	11,54197543	6,63	1,740871106	4,31E-101	Arntl	4,83E-101
MA1481.1	923	2018	9,369607146	5,045	1,85720657	8,66E-100	DRGX	9,68E-100
MA0722.1	923	2018	9,369607146	5,045	1,85720657	8,66E-100	VAX1	9,68E-100
MA0028.2	1272	3083	12,91239468	7,7075	1,675302586	1,05E-99	ELK1	1,17E-99
MA1560.1	836	1766	8,486448076	4,415	1,922185295	1,19E-99	SOHLH2	1,32E-99
MA0843.1	722	1451	7,329205157	3,6275	2,02045628	2,03E-98	TEF	2,26E-98
MA0889.1	879	1901	8,922951985	4,7525	1,877528035	5,35E-98	GBX1	5,95E-98
MA0705.1	849	1815	8,618414374	4,5375	1,899375069	8,15E-98	Lhx8	9,00E-98
MA0701.2	849	1815	8,618414374	4,5375	1,899375069	8,15E-98	LHX9	9,00E-98
MA0068.2	849	1815	8,618414374	4,5375	1,899375069	8,15E-98	PAX4	9,00E-98
MA0075.3	849	1815	8,618414374	4,5375	1,899375069	8,15E-98	PRRX2	9,00E-98
MA1577.1	849	1815	8,618414374	4,5375	1,899375069	8,15E-98	TLX2	9,00E-98
MA0623.2	910	1994	9,237640849	4,985	1,853087432	1,14E-97	NEUROG1	1,25E-97
MA1501.1	996	2252	10,11064867	5,63	1,795852338	2,38E-97	HOXB7	2,62E-97
MA0754.1	623	1194	6,324231043	2,985	2,118670366	8,22E-96	CUX1	9,03E-96
MA1557.1	780	1630	7,91797787	4,075	1,943062054	1,11E-95	SMAD5	1,22E-95
MA0485.2	824	1757	8,364633032	4,3925	1,904298926	1,46E-95	HOXC9	1,60E-95
MA1497.1	1044	2416	10,59790884	6,04	1,754620669	7,60E-95	HOXA6	8,31E-95
MA0635.1	827	1780	8,395086793	4,45	1,886536358	2,52E-93	BARHL2	2,75E-93

MA1476.1	911	2043	9,247792102	5,1075	1,810629878	4,89E-91	DLX5	5,34E-91
MA0826.1	864	1907	8,770683179	4,7675	1,839681842	1,55E-90	OLIG1	1,69E-90
MA1500.1	981	2260	9,95837986	5,65	1,762545108	3,44E-90	HOXB6	3,74E-90
MA0686.1	1056	2492	10,71972389	6,23	1,72066194	7,04E-90	SPDEF	7,64E-90
MA0639.1	660	1326	6,699827429	3,315	2,021064081	7,04E-90	DBP	7,64E-90
MA1505.1	774	1653	7,857070348	4,1325	1,901287441	3,43E-89	HOXC8	3,72E-89
MA0678.1	848	1875	8,60826312	4,6875	1,836429466	2,53E-88	OLIG2	2,74E-88
MA0668.1	801	1740	8,131154198	4,35	1,86923085	7,19E-88	NEUROD2	7,77E-88
MA0886.1	809	1766	8,212364227	4,415	1,860105148	1,77E-87	EMX2	1,90E-87
MA1561.1	849	1890	8,618414374	4,725	1,824003042	1,35E-86	SOX12	1,45E-86
MA1583.1	698	1457	7,085575069	3,6425	1,945250534	1,13E-85	ZFP57	1,22E-85
MA1498.1	829	1839	8,415389301	4,5975	1,830427254	2,04E-85	HOXA7	2,19E-85
MA0664.1	846	1890	8,587960613	4,725	1,817557802	2,46E-85	MLXIPL	2,63E-85
MA0642.1	785	1721	7,968734139	4,3025	1,852117173	1,02E-83	EN2	1,09E-83
MA0259.1	746	1609	7,572835245	4,0225	1,882619079	1,95E-83	ARNT::HIF1A	2,09E-83
MA0844.1	699	1475	7,095726322	3,6875	1,924264765	3,39E-83	XBP1	3,62E-83
MA0663.1	860	1950	8,730078165	4,875	1,790785265	8,03E-83	MLX	8,56E-83
MA0875.1	712	1515	7,22769262	3,7875	1,908301682	9,12E-83	BARX1	9,70E-83
MA0680.1	763	1665	7,745406558	4,1625	1,860758332	1,83E-82	PAX7	1,95E-82
MA1546.1	722	1557	7,329205157	3,8925	1,882904343	9,94E-81	PAX3(var.2)	1,05E-80
MA1518.1	874	2009	8,872195716	5,0225	1,766489939	1,40E-80	LHX1	1,49E-80
MA0725.1	684	1453	6,943457517	3,6325	1,911481767	7,80E-80	VSX1	8,24E-80
MA0726.1	684	1453	6,943457517	3,6325	1,911481767	7,80E-80	VSX2	8,24E-80
MA0145.3	807	1816	8,19206172	4,54	1,804418881	1,86E-79	TFCP2	1,96E-79
MA0780.1	766	1703	7,775860319	4,2575	1,826391149	3,21E-78	PAX3	3,38E-78
MA1106.1	678	1450	6,882549995	3,625	1,898634481	1,37E-77	HIF1A	1,44E-77
MA0755.1	581	1194	5,897878388	2,985	1,975838656	2,75E-74	CUX2	2,89E-74
MA0527.1	587	1222	5,95878591	3,055	1,950502753	2,03E-72	ZBTB33	2,13E-72
MA0464.2	783	1839	7,948431631	4,5975	1,728859517	5,48E-67	BHLHE40	5,74E-67
MA0636.1	768	1801	7,796162826	4,5025	1,731518673	5,12E-66	BHLHE41	5,35E-66
MA0612.2	581	1246	5,897878388	3,115	1,89337 99	7,77E-66	EMX1	8,07E-66
MA0900.2	581	1246	5,897878388	3,115	1,89337 99	7,77E-66	HOXA2	8,07E-66
MA0902.2	581	1246	5,897878388	3,115	1,89337 99	7,77E-66	HOXB2	8,07E-66
MA0912.2	581	1246	5,897878388	3,115	1,89337 99	7,77E-66	HOXD3	8,07E-66
MA0132.2	581	1246	5,897878388	3,115	1,89337	7,77E-66	PDX1	8,07E-66

Supplementary Table 2

					99		
MA0882.1	651	1473	6,608466146	3,6825	1,79455<br>9714	9,81E-63 DLX6	1,02E-62
MA0067.1	509	1062	5,166988123	2,655	1,94613<br>4886	2,29E-62 Pax2	2,37E-62
MA0724.1	570	1247	5,786214598	3,1175	1,85604<br>3175	6,93E-61 VENTX	7,17E-61
MA1495.1	553	1198	5,613643285	2,995	1,87433<br>8326	8,13E-61 HOXA1	8,38E-61
MA0158.2	553	1198	5,613643285	2,995	1,87433<br>8326	8,13E-61 HOXA5	8,38E-61
MA0904.2	553	1198	5,613643285	2,995	1,87433<br>8326	8,13E-61 HOXB5	8,38E-61
MA0604.1	423	860	4,293980307	2,15	1,99720<br>0143	1,06E-55 Atf1	1,09E-55
MA0777.1	420	872	4,263526546	2,18	1,95574<br>6122	3,12E-52 MYBL2	3,21E-52
MA0466.2	495	1088	5,024870572	2,72	1,84737<br>8887	3,84E-52 CEBPB	3,94E-52
MA0837.1	461	996	4,679727946	2,49	1,87940<br>8814	3,91E-51 CEBPE	4,01E-51
MA1509.1	473	1033	4,801542991	2,5825	1,85926<br>1565	8,29E-51 IRF6	8,48E-51
MA1502.1	494	1116	5,014719318	2,79	1,79739<br>0437	7,95E-48 HOXB8	8,12E-48
MA0877.2	371	773	3,766115115	1,9325	1,94883<br>059	8,98E-46 BARHL1	9,17E-46
MA0838.1	445	1002	4,517307888	2,505	1,80331<br>6522	1,40E-43 CEBPG	1,42E-43
MA1545.1	325	664	3,299157446	1,66	1,98744<br>4245	1,99E-42 OVOL2	2,03E-42
MA0615.1	263	505	2,669779718	1,2625	2,11467<br>7004	1,56E-40 Gmeb1	1,59E-40
MA1544.1	325	687	3,299157446	1,7175	1,92090<br>681	1,24E-38 OVOL1	1,25E-38
MA0637.1	390	884	3,958988935	2,21	1,79139<br>7708	1,64E-37 CENPB	1,66E-37
MA0776.1	419	988	4,253375292	2,47	1,72201<br>4288	2,12E-35 MYBL1	2,14E-35
MA0862.1	288	626	2,92356106	1,565	1,86809<br>0134	9,63E-32 GMEB2	9,73E-32

MA0749.1	198	405	2,009948229	1,0125	1,985134053	2,82E-26	ZBED1	2,85E-26
MA0594.2	228	502	2,314485839	1,255	1,844211824	1,76E-24	HOXA9	1,77E-24
MA0711.1	217	511	2,202822049	1,2775	1,724322543	4,92E-19	OTX1	4,95E-19
MA0469.3	207	481	2,101309512	1,2025	1,747450737	5,22E-19	E2F3	5,25E-19
MA0024.3	267	701	2,710384733	1,7525	1,546581873	1,17E-15	E2F1	1,17E-15
MA0470.2	281	790	2,852502284	1,975	1,444304954	2,75E-12	E2F4	2,76E-12
MA0864.2	278	811	2,822048523	2,0275	1,391885831	3,15E-10	E2F2	3,16E-10
MA0139.1	4209	16356	42,72662674	40,89	1,044916281	1,05E-05	CTCF	1,05E-05

Extended data 2: Enriched motifs of differential accessibility peaks

scATACseq data obtained from TGFβR-KO and TGFβR-WT mice were integrated. Differential accessibility (DA) in TGFβR-KO vs. TGFβR- WT was obtained using the 'FindMarkers' function of the Seurat package. After motif annotation, over-represented TF motifs in those DA peaks were identified with the 'FindMotifs' function of the Signac package.

Regulon	CellType	RelativeActivity
Ets2_extended (313g)	Tumorigenic	1,524655199
Myc (264g)	Tumorigenic	1,268489621
Atf6 (86g)	Tumorigenic	2,213832517
Xbp1 (180g)	Tumorigenic	1,762549374
Egr2 (79g)	Tumorigenic	0,846397939
Egr3 (52g)	Tumorigenic	0,845303261
Ddit3 (14g)	Tumorigenic	1,822275391
Taf7 (138g)	Tumorigenic	1,56852351
Cebpb (21g)	Tumorigenic	1,681009504
Erf_extended (50g)	Tumorigenic	1,916525402
Egr1 (351g)	Tumorigenic	1,443427117
Rel (209g)	Tumorigenic	1,871643064
Irf4 (68g)	Tumorigenic	1,90442161
Atf1 (20g)	Tumorigenic	0,127216529
Nr1d1 (46g)	Tumorigenic	0,908741524
Rorc (26g)	Tumorigenic	0,38230917
Fli1 (909g)	Tumorigenic	0,572249199
Klf2 (187g)	Tumorigenic	0,966881942
Elf4 (1429g)	Tumorigenic	0,216354839
Runx1 (12g)	Tumorigenic	0,857775487
Prdm1_extended (107g)	Tumorigenic	1,7065069
Zmiz1 (12g)	Tumorigenic	1,058438517
Rara_extended (333g)	Tumorigenic	1,56488953
Irf7 (115g)	Tumorigenic	2,007602832
Runx3 (79g)	Tumorigenic	1,256586378
Tbx21 (22g)	Tumorigenic	1,261126801
Tcf3_extended (64g)	Tumorigenic	1,554068499
Mlx_extended (52g)	Tumorigenic	0,775198185
Ywhaz (26g)	Tumorigenic	0,043188181
Srebf1_extended (743g)	Tumorigenic	0,203372578
Srf (239g)	Tumorigenic	1,00378792
Sp2 (374g)	Tumorigenic	1,017176289
Rora (28g)	Tumorigenic	1,717106662
Crem (481g)	Tumorigenic	1,973794721
Bhlhe40_extended (990g)	Tumorigenic	1,850871848
Fosl2 (148g)	Tumorigenic	1,886135025
Nfil3 (146g)	Tumorigenic	1,802691215
Fosb (269g)	Tumorigenic	1,782804469
Jund (309g)	Tumorigenic	1,776316884
Junb (215g)	Tumorigenic	1,81885998
Jun (190g)	Tumorigenic	1,519403772
Fos (212g)	Tumorigenic	1,658817229
Atf4 (199g)	Tumorigenic	1,758400413
Maff_extended (350g)	Tumorigenic	1,830381785
Tgif1 (126g)	Tumorigenic	1,867519725
Klf4_extended (26g)	Tumorigenic	1,807576117
Atf3 (266g)	Tumorigenic	1,757649677
Tgif2 (88g)	Tumorigenic	2,100804279
Sap30_extended (155g)	Tumorigenic	1,58868873
Hspa5 (11g)	Tumorigenic	2,006869028

Mxi1 (14g)	Tumorigenic	1,458038374
Hlf (54g)	Tumorigenic	1,001299105
Foxo1 (279g)	Tumorigenic	1,730788733
Klf6 (39g)	Tumorigenic	1,901433214
E4f1 (137g)	Tumorigenic	1,523671284
Ets1_extended (548g)	Tumorigenic	0,415940251
Elf1_extended (222g)	Tumorigenic	0,200190027
Chd2 (137g)	Tumorigenic	1,302543956
Etv3_extended (207g)	Tumorigenic	0,070874416
Batf_extended (22g)	Tumorigenic	0,213887513
Bcl3 (59g)	Tumorigenic	0,593004748
Relb (119g)	Tumorigenic	0,059224166
Nfkb2 (73g)	Tumorigenic	1,390504735
Elk1 (415g)	Tumorigenic	0,492106714
Mnt (10g)	Tumorigenic	1,454332745
Klf9_extended (27g)	Tumorigenic	1,718539646
Mafk_extended (216g)	Tumorigenic	2,120805441
Hivep1_extended (20g)	Tumorigenic	1,913411569
Klf16_extended (241g)	Tumorigenic	1,712906699
Klf10_extended (12g)	Tumorigenic	1,122221522
Stat3_extended (61g)	Tumorigenic	0,042911428
Cebpd_extended (39g)	Tumorigenic	0,642753517
Foxj3 (12g)	Tumorigenic	0,663516683
Brf1_extended (468g)	Tumorigenic	0,770755738
Klf13_extended (10g)	Tumorigenic	0,965253788
Nfe2l1_extended (22g)	Tumorigenic	0,136233471
Foxo3_extended (17g)	Tumorigenic	0,323646076
Polr3a_extended (15g)	Tumorigenic	0,238865815
Nfic_extended (19g)	Tumorigenic	0,246051831
Sirt6_extended (28g)	Tumorigenic	1,167981609
Rxrb_extended (23g)	Tumorigenic	0,097739052
Mta3_extended (11g)	Tumorigenic	0,06375643
Stat5b_extended (21g)	Tumorigenic	0,275856434
Atf6b (29g)	Tumorigenic	0,162254889
Sp3 (60g)	Tumorigenic	0,295310033
Arnt2 (43g)	Tumorigenic	0,15231658

Supplementary Table 3: Top Regulon list in tumorigenic cells

Gene regulatory network analysis was performed on scRNAseq data using the SCENIC package. All significant regulons specific to the "Tumorigenic" cluster in TGFβR-KO cells, and their relative activity, are listed.

Supplementary Table 4

motif	p_val	avg_diff	pct.1	pct.2	p_val_adj	gene
MA1653.1	3,13E-87	-0,442248094	0,33	0,59	2,34E-84	ZNF148
MA0688.1	3,69E-87	1,1870285	0,61	0,32	2,76E-84	TBX2
MA0690.1	1,67E-84	1,274055201	0,6	0,32	1,25E-81	TBX21
MA1567.1	7,65E-83	1,251458756	0,6	0,34	5,70E-80	TBX6
MA0800.1	2,38E-82	1,237346535	0,59	0,32	1,78E-79	EOMES
MA0502.2	1,88E-81	-0,682925727	0,34	0,6	1,40E-78	NFYB
MA0802.1	1,14E-76	1,203792955	0,6	0,34	8,49E-74	TBR1
MA0741.1	2,21E-76	-0,439382917	0,32	0,58	1,65E-73	KLF16
MA0689.1	8,36E-72	1,076096683	0,6	0,34	6,24E-69	TBX20
MA1627.1	5,20E-68	-0,507836668	0,31	0,56	3,88E-65	Wt1
MA0807.1	5,81E-68	1,122073128	0,59	0,35	4,34E-65	TBX5
MA0060.3	8,22E-68	-0,586756958	0,36	0,59	6,13E-65	NFYA
MA0801.1	4,48E-66	1,162903278	0,6	0,36	3,34E-63	MGA
MA0803.1	4,48E-66	1,162903278	0,6	0,36	3,34E-63	TBX15
MA0805.1	2,87E-65	1,045197475	0,6	0,35	2,14E-62	TBX1
MA0806.1	3,11E-65	1,026176235	0,59	0,35	2,32E-62	TBX4
MA1564.1	3,18E-63	-0,392095714	0,33	0,56	2,37E-60	SP9
MA0516.2	1,48E-60	-0,414548117	0,34	0,56	1,11E-57	SP2
MA1566.1	2,56E-59	1,041373595	0,58	0,36	1,91E-56	TBX3
MA1475.1	8,53E-59	0,649976112	0,62	0,39	6,37E-56	CREB3L4(var.2)
MA1644.1	5,77E-58	-0,568572981	0,36	0,59	4,31E-55	NFYC
MA0162.4	9,58E-55	-0,472704857	0,33	0,55	7,15E-52	EGR1
MA0732.1	1,24E-53	-0,533405321	0,33	0,55	9,27E-51	EGR3
MA0624.1	1,37E-46	-0,717879782	0,32	0,53	1,02E-43	NFATC1
MA0028.2	8,05E-46	0,564324863	0,6	0,41	6,00E-43	ELK1
MA0685.1	7,01E-45	-0,362471786	0,35	0,54	5,23E-42	SP4
MA0603.1	4,53E-44	-0,544468276	0,37	0,55	3,38E-41	Arntl
MA0759.1	1,40E-43	0,57329173	0,59	0,39	1,04E-40	ELK3
MA1140.2	2,32E-43	0,587148855	0,58	0,39	1,73E-40	JUNB(var.2)
MA0117.2	2,55E-43	-0,739502078	0,36	0,55	1,91E-40	Mafb
MA0652.1	1,10E-42	0,671905155	0,57	0,38	8,18E-40	IRF8
MA0620.3	1,71E-40	-0,632962729	0,38	0,57	1,28E-37	MITF
MA0828.1	1,87E-39	-0,526138439	0,39	0,57	1,39E-36	SREBF2(var.2)
MA0625.1	3,62E-39	-0,656806602	0,32	0,51	2,70E-36	NFATC3
MA1522.1	6,38E-38	-0,407811455	0,38	0,55	4,76E-35	MAZ
MA0472.2	1,22E-37	-0,517138436	0,35	0,54	9,09E-35	EGR2
MA0664.1	2,36E-36	-0,475658479	0,39	0,56	1,76E-33	MLXIPL
MA0871.2	5,64E-36	-0,525762548	0,4	0,56	4,21E-33	TFEC
MA1419.1	2,26E-35	0,613590206	0,56	0,38	1,68E-32	IRF4
MA1464.1	2,58E-35	-0,500808748	0,39	0,55	1,93E-32	ARNT2
MA1565.1	3,98E-35	0,715620917	0,56	0,38	2,97E-32	TBX18
MA1420.1	6,35E-35	0,515731364	0,55	0,39	4,74E-32	IRF5
MA0511.2	1,86E-34	0,74877878	0,56	0,39	1,39E-31	RUNX2
MA0908.1	2,09E-34	-0,450414964	0,36	0,55	1,56E-31	HOXD11
MA0692.1	2,45E-34	-0,530029612	0,39	0,56	1,83E-31	TFEB
MA0768.1	5,20E-34	-0,541377839	0,36	0,55	3,88E-31	LEF1
MA0076.2	5,58E-34	0,503095124	0,57	0,41	4,16E-31	ELK4
MA0609.2	3,75E-33	0,543946122	0,56	0,41	2,80E-30	CREM
MA0149.1	4,16E-33	-0,341783877	0,38	0,56	3,11E-30	EWSR1-FLI1
MA1503.1	8,75E-33	-0,460761023	0,36	0,55	6,53E-30	HOXB9
MA0604.1	2,67E-32	0,412288567	0,58	0,41	1,99E-29	Atf1
MA0464.2	4,45E-32	-0,467852907	0,39	0,55	3,32E-29	BHLHE40
MA0763.1	5,60E-32	0,497315233	0,57	0,41	4,17E-29	ETV3
MA0131.2	1,30E-31	0,446969035	0,58	0,4	9,69E-29	HINFP
MA0769.2	1,32E-31	-0,575867112	0,37	0,54	9,83E-29	TCF7
MA0772.1	2,42E-31	0,452961403	0,55	0,4	1,81E-28	IRF7
MA0002.2	3,48E-31	0,722033511	0,58	0,4	2,59E-28	RUNX1

MA1525.1	7,78E-31	-0,609877974	0,32	0,5	5,80E-28	NFATC4
MA0834.1	2,97E-30	0,485284705	0,57	0,4	2,22E-27	ATF7
MA0156.2	1,18E-29	0,48040467	0,58	0,42	8,79E-27	FEV
MA0663.1	1,41E-29	-0,419977459	0,4	0,55	1,05E-26	MLX
MA0471.2	1,69E-29	-0,298509733	0,37	0,54	1,26E-26	E2F6
MA1516.1	1,83E-29	0,350858308	0,53	0,38	1,36E-26	KLF3
MA0152.1	2,23E-29	-0,438842061	0,35	0,51	1,66E-26	NFATC2
MA1129.1	5,06E-29	0,459760914	0,57	0,4	3,77E-26	FOSL1::JUN(var.2)
MA0636.1	1,25E-28	-0,428489394	0,39	0,55	9,35E-26	BHLHE41
MA0842.2	1,85E-28	-0,590970322	0,38	0,53	1,38E-25	NRL
MA0844.1	4,86E-28	0,425379263	0,58	0,41	3,63E-25	XBP1
MA0523.1	6,35E-28	-0,51332045	0,37	0,55	4,74E-25	TCF7L2
MA1127.1	2,18E-27	0,433187497	0,58	0,41	1,63E-24	FOSB::JUN
MA0733.1	3,70E-27	-0,394882642	0,37	0,53	2,76E-24	EGR4
MA0653.1	4,58E-27	0,486662398	0,57	0,4	3,42E-24	IRF9
MA0656.1	6,11E-27	0,483477736	0,56	0,41	4,55E-24	JDP2(var.2)
MA0475.2	5,80E-26	0,445316354	0,58	0,41	4,33E-23	FLI1
MA0495.3	2,39E-25	-0,522611729	0,38	0,53	1,78E-22	MAFF
MA0747.1	3,00E-25	-0,264462017	0,38	0,52	2,24E-22	SP8
MA0485.2	3,91E-25	-0,398625828	0,39	0,54	2,91E-22	HOXC9
MA1116.1	4,47E-25	-0,42582957	0,39	0,52	3,34E-22	RBPJ
MA0660.1	8,26E-25	-0,408042239	0,4	0,55	6,16E-22	MEF2B
MA0765.2	6,12E-24	0,437150229	0,57	0,43	4,56E-21	ETV5
MA1136.1	2,78E-23	0,406224568	0,56	0,41	2,07E-20	FOSB::JUNB(var.2)
MA0493.1	3,05E-23	0,456761246	0,52	0,38	2,28E-20	Klf1
MA0740.1	3,82E-23	-0,271686625	0,39	0,51	2,85E-20	KLF14
MA1421.1	7,27E-23	-0,444603414	0,39	0,53	5,43E-20	TCF7L1
MA0645.1	8,93E-23	0,452722663	0,55	0,42	6,67E-20	ETV6
MA0606.1	1,50E-22	-0,461302179	0,34	0,5	1,12E-19	NFAT5
MA0750.2	6,37E-22	0,368586376	0,55	0,42	4,75E-19	ZBTB7A
MA1517.1	7,38E-22	0,312448979	0,51	0,37	5,50E-19	KLF6
MA0831.2	7,39E-22	-0,455470416	0,4	0,54	5,52E-19	TFE3
MA0051.1	8,74E-22	0,390238446	0,53	0,39	6,52E-19	IRF2
MA1143.1	1,31E-21	0,351192833	0,55	0,42	9,80E-19	FOSL1::JUND(var.2)
MA0867.2	3,29E-21	-0,408836261	0,41	0,53	2,46E-18	SOX4
MA1506.1	5,21E-21	-0,364063512	0,4	0,53	3,88E-18	HOXD10
MA0006.1	8,22E-21	-0,31135687	0,41	0,54	6,13E-18	Ahr::Arnt
MA0474.2	9,24E-21	0,422556532	0,56	0,41	6,89E-18	ERG
MA0098.3	9,33E-21	0,43376427	0,55	0,42	6,96E-18	ETS1
MA0760.1	1,35E-20	0,439542147	0,55	0,43	1,01E-17	ERF
MA0703.2	1,51E-20	-0,322413559	0,39	0,53	1,13E-17	LMX1B
MA0668.1	5,20E-20	-0,39132587	0,39	0,52	3,88E-17	NEUROD2
MA0795.1	2,73E-19	-0,430384949	0,39	0,53	2,04E-16	SMAD3
MA1131.1	3,41E-19	0,36263937	0,55	0,43	2,54E-16	FOSL2::JUN(var.2)
MA0684.2	5,29E-19	0,476156757	0,52	0,4	3,95E-16	RUNX3
MA1559.1	7,11E-19	-0,414735534	0,41	0,52	5,30E-16	SNAI3
MA1484.1	1,00E-18	0,383721288	0,56	0,42	7,48E-16	ETS2
MA0038.2	1,75E-18	-0,347741176	0,4	0,53	1,31E-15	GFI1
MA1139.1	2,11E-18	0,367024493	0,55	0,42	1,58E-15	FOSL2::JUNB(var.2)
MA0442.2	4,38E-18	-0,343458758	0,4	0,52	3,27E-15	SOX10
MA0826.1	6,27E-18	-0,373869179	0,41	0,52	4,68E-15	OLIG1
MA0600.2	9,11E-18	0,390153476	0,54	0,43	6,79E-15	RFX2
MA0659.2	1,65E-17	-0,457440816	0,39	0,52	1,23E-14	MAFG
MA1115.1	2,23E-17	-0,376053406	0,38	0,51	1,67E-14	POU5F1
MA0605.2	4,42E-17	0,377296683	0,54	0,41	3,30E-14	ATF3

Supplementary Table 4

MA0461.2	7,13E-17	-0,36462912	0,41	0,52	5,32E-14	Atoh1
MA0465.2	7,46E-17	-0,30114055	0,4	0,53	5,56E-14	CDX2
MA0817.1	9,32E-17	-0,348349869	0,4	0,52	6,95E-14	BHLHE23
MA0093.3	1,22E-15	-0,445464	0,42	0,53	9,10E-13	USF1
MA0499.2	1,25E-15	-0,373665884	0,41	0,52	9,35E-13	MYOD1
MA0507.1	1,77E-15	-0,321287965	0,39	0,51	1,32E-12	POU2F2
MA1557.1	5,06E-15	-0,363476739	0,4	0,52	3,78E-12	SMAD5
MA1133.1	5,61E-15	0,337600186	0,53	0,43	4,18E-12	JUN::JUNB(var.2)
MA0601.1	7,89E-15	-0,267455891	0,41	0,53	5,89E-12	Arid3b
MA1128.1	8,52E-15	0,738988921	0,49	0,38	6,35E-12	FOSL1::JUN
MA0526.3	1,08E-14	-0,4269998	0,42	0,52	8,03E-12	USF2
MA0829.2	1,30E-14	-0,343526124	0,42	0,53	9,68E-12	SREBF1(var.2)
MA0035.4	1,79E-14	-0,31802711	0,43	0,52	1,33E-11	GATA1
MA1584.1	1,97E-14	0,277078005	0,54	0,43	1,47E-11	ZIC5
MA0525.2	2,39E-14	-0,356461104	0,41	0,52	1,79E-11	TP63
MA0905.1	3,35E-14	-0,288477683	0,41	0,52	2,50E-11	HOXC10
MA1558.1	5,47E-14	-0,337675168	0,41	0,51	4,08E-11	SNAI1
MA1597.1	5,56E-14	-0,325559839	0,41	0,51	4,15E-11	ZNF528
MA1099.2	6,77E-14	0,255762602	0,54	0,43	5,05E-11	HES1
MA1130.1	8,59E-14	0,719362388	0,49	0,39	6,41E-11	FOSL2::JUN
MA1480.1	8,81E-14	-0,304024089	0,41	0,51	6,57E-11	DPRX
MA0745.2	9,52E-14	-0,360119383	0,43	0,53	7,10E-11	SNAI2
MA1135.1	9,93E-14	0,713343564	0,48	0,39	7,41E-11	FOSB::JUNB
MA1631.1	2,21E-13	-0,299999918	0,41	0,52	1,65E-10	ASCL1(var.2)
MA1468.1	2,71E-13	-0,386388704	0,42	0,52	2,02E-10	ATOH7
MA0521.1	3,21E-13	-0,343738427	0,41	0,53	2,40E-10	Tcf12
MA1137.1	3,24E-13	0,696376188	0,49	0,38	2,42E-10	FOSL1::JUNB
MA0748.2	7,67E-13	0,261539476	0,56	0,46	5,72E-10	YY2
MA1134.1	8,62E-13	0,71277236	0,48	0,39	6,43E-10	FOS::JUNB
MA1561.1	1,11E-12	-0,275106097	0,42	0,51	8,31E-10	SOX12
MA0155.1	1,33E-12	-0,304366278	0,42	0,52	9,92E-10	INSM1
MA0508.3	1,42E-12	-0,264094421	0,41	0,51	1,06E-09	PRDM1
MA0009.2	1,68E-12	0,341915257	0,52	0,42	1,25E-09	TBXT
MA0830.2	1,84E-12	-0,311334973	0,41	0,51	1,37E-09	TCF4
MA1145.1	2,19E-12	0,319707876	0,52	0,43	1,63E-09	FOSL2::JUND(var.2)
MA1138.1	2,32E-12	0,679454767	0,49	0,39	1,73E-09	FOSL2::JUNB
MA0029.1	2,69E-12	-0,269154273	0,41	0,52	2,01E-09	Mecom
MA1472.1	2,70E-12	-0,360913167	0,42	0,52	2,02E-09	BHLHA15(var.2)
MA0072.1	2,91E-12	-0,621059831	0,37	0,48	2,17E-09	RORA(var.2)
MA0057.1	4,96E-12	-0,265734995	0,4	0,51	3,70E-09	MZF1(var.2)
MA0853.1	4,99E-12	-0,262728886	0,42	0,52	3,72E-09	Alx4
MA0755.1	7,99E-12	-0,256702052	0,4	0,51	5,96E-09	CUX2
MA1141.1	8,53E-12	0,685423286	0,49	0,39	6,36E-09	FOS::JUND
MA0786.1	8,59E-12	-0,252585098	0,4	0,51	6,41E-09	POU3F1
MA1467.1	1,40E-11	-0,346982622	0,41	0,52	1,04E-08	ATOH1(var.2)
MA0478.1	1,69E-11	0,694571511	0,48	0,39	1,26E-08	FOSL2
MA0651.1	1,70E-11	-0,252138836	0,41	0,53	1,27E-08	HOXC11
MA0476.1	1,91E-11	0,656793217	0,47	0,39	1,42E-08	FOS
MA1144.1	1,93E-11	0,666254879	0,48	0,39	1,44E-08	FOSL2::JUND
MA0509.2	2,94E-11	0,330620663	0,51	0,43	2,19E-08	RFX1
MA0627.2	3,78E-11	-0,276780595	0,41	0,51	2,82E-08	POU2F3
MA0821.1	5,41E-11	0,310632155	0,52	0,42	4,04E-08	HES5
MA0858.1	5,64E-11	-0,326724626	0,43	0,52	4,21E-08	Rarb(var.2)
MA1647.1	6,64E-11	-0,281883932	0,42	0,52	4,96E-08	PRDM4
MA0623.2	7,40E-11	-0,300922381	0,44	0,52	5,52E-08	NEUROG1
MA0099.3	7,78E-11	0,66046699	0,48	0,4	5,81E-08	FOS::JUN

MA1648.1	9,58E-11	-0,268763454	0,43	0,51	7,15E-08	TCF12(var.2)
MA0522.3	1,11E-10	-0,268878063	0,43	0,51	8,26E-08	TCF3
MA1149.1	1,28E-10	-0,339942936	0,43	0,5	9,53E-08	RARA::RXRG
MA1632.1	2,01E-10	0,347871526	0,52	0,43	1,50E-07	ATF2
MA0019.1	4,97E-10	-0,252084808	0,42	0,51	3,70E-07	Ddit3::Cebpa
MA1520.1	6,13E-10	-0,380131019	0,42	0,5	4,57E-07	MAF
MA0258.2	6,95E-10	-0,318529152	0,42	0,5	5,18E-07	ESR2
MA0840.1	7,19E-10	0,252442825	0,53	0,45	5,37E-07	Creb5
MA1637.1	7,20E-10	-0,296726715	0,42	0,51	5,37E-07	EBF3
MA1153.1	1,19E-09	-0,304142168	0,42	0,51	8,84E-07	Smad4
MA0489.1	1,63E-09	0,6096156	0,48	0,39	1,22E-06	JUN(var.2)
MA0742.1	1,78E-09	0,253247049	0,49	0,41	1,33E-06	Klf12
MA1496.1	4,38E-09	-0,259840836	0,43	0,51	3,26E-06	HOXA4
MA1622.1	5,14E-09	0,619819963	0,47	0,4	3,83E-06	Smad2::Smad3
MA1554.1	6,15E-09	0,266805985	0,52	0,44	4,59E-06	RFX7
MA1604.1	6,61E-09	-0,27680147	0,43	0,52	4,93E-06	Ebf2
MA0500.2	1,59E-08	-0,304938007	0,43	0,52	1,19E-05	MYOG
MA0669.1	1,60E-08	-0,27448399	0,43	0,51	1,20E-05	NEUROG2
MA1618.1	1,62E-08	-0,275468111	0,42	0,51	1,21E-05	Ptf1a
MA0159.1	1,68E-08	-0,297013463	0,43	0,5	1,26E-05	RARA::RXRA
MA0816.1	2,42E-08	-0,290902502	0,42	0,51	1,81E-05	Ascl2
MA0488.1	2,56E-08	0,304734859	0,51	0,43	1,91E-05	JUN
MA0091.1	2,58E-08	-0,265447376	0,44	0,5	1,93E-05	TAL1::TCF3
MA0667.1	2,59E-08	-0,266169493	0,43	0,5	1,93E-05	MYF6
MA0089.2	3,06E-08	0,4148842	0,49	0,43	2,28E-05	NFE2L1
MA0477.2	6,01E-08	0,574411797	0,47	0,4	4,48E-05	FOSL1
MA0018.4	9,15E-08	0,303559032	0,51	0,43	6,83E-05	CREB1
MA1142.1	1,03E-07	0,530985438	0,46	0,4	7,68E-05	FOSL1::JUND
MA1521.1	2,48E-07	-0,30826642	0,42	0,5	0,000185085	MAFA
MA0007.3	2,48E-07	-0,27200713	0,42	0,5	0,000185266	Ar
MA0059.1	3,36E-07	0,274405804	0,51	0,43	0,000250462	MAX::MYC
MA1132.1	4,75E-07	0,461705229	0,47	0,4	0,000354546	JUN::JUNB
MA0655.1	7,16E-07	0,464337424	0,47	0,4	0,000534053	JDP2
MA0841.1	8,84E-07	0,473182664	0,47	0,41	0,000659445	NFE2
MA0150.2	1,03E-06	0,34511413	0,51	0,43	0,00077091	Nfe2l2
MA0835.2	5,22E-06	0,494630366	0,45	0,41	0,00389131	BATF3
MA1634.1	1,26E-05	0,484984452	0,45	0,41	0,009394712	BATF
MA0071.1	1,86E-05	-0,333713859	0,42	0,48	0,013883881	RORA
MA0491.2	1,88E-05	0,463364081	0,45	0,4	0,014040915	JUND
MA1633.1	2,30E-05	0,370308951	0,47	0,42	0,017173977	BACH1
MA0462.2	3,08E-05	0,468512597	0,45	0,41	0,022989893	BATF::JUN

Supplementary Table 4: RunChromVAR analysis

As an alternative approach to the enriched motif analysis on DA peaks, the Signac's 'RunChromVAR' function was used to directly calculate TF motif enrichments. The resulting ChromVAR assay was used to compare differential enrichment in TGFβR-KO vs. TGFβR-WT cells using Seurat's 'FindMarkers' function. All differentially enriched motifs are shown.

Th17 cells

RORC
IL17A
IL17F
IL22
CCR6
IL23R
TMEM176A

Supplementary Table 5: Human T_H17 cell signature

List of genes whose the expression was used to characterize T_H17 clusters for human sample analysis.
