# Supplementary material for: An intestinal TH17 cell-derived subset can initiate cancer
Source: Nat Immunol. 2024 Jul 26;25(9):1637–49. doi: 10.1038/s41590-024-01909-7 (PMC11362008; doi:10.1038/s41590-024-01909-7)
Supplement: Supplementary file 2 — Reporting Summary [file 41590_2024_1909_MOESM2_ESM.pdf]

nature portfolio

Corresponding author(s): Julien C Marie

Last updated by author(s): Jun 18, 2024

Reporting Summary

Nature Portfolio wishes to improve the reproducibility of the work that we publish. This form provides structure for consistency and transparency in reporting. For further information on Nature Portfolio policies, see our Editorial Policies and the Editorial Policy Checklist.

Statistics

For all statistical analyses, confirm that the following items are present in the figure legend, table legend, main text, or Methods section.

n/a	Confirmed	
☐	☒	The exact sample size (*n*) for each experimental group/condition, given as a discrete number and unit of measurement
☐	☒	A statement on whether measurements were taken from distinct samples or whether the same sample was measured repeatedly
☐	☒	The statistical test(s) used AND whether they are one- or two-sided<br>*Only common tests should be described solely by name; describe more complex techniques in the Methods section.*
☒	☐	A description of all covariates tested
☒	☐	A description of any assumptions or corrections, such as tests of normality and adjustment for multiple comparisons
☐	☒	A full description of the statistical parameters including central tendency (e.g. means) or other basic estimates (e.g. regression coefficient) AND variation (e.g. standard deviation) or associated estimates of uncertainty (e.g. confidence intervals)
☐	☒	For null hypothesis testing, the test statistic (e.g. *F*, *t*, *r*) with confidence intervals, effect sizes, degrees of freedom and *P* value noted<br>*Give P values as exact values whenever suitable.*
☒	☐	For Bayesian analysis, information on the choice of priors and Markov chain Monte Carlo settings
☒	☐	For hierarchical and complex designs, identification of the appropriate level for tests and full reporting of outcomes
☒	☐	Estimates of effect sizes (e.g. Cohen's *d*, Pearson's *r*), indicating how they were calculated

Our web collection on statistics for biologists contains articles on many of the points above.

Software and code

Policy information about availability of computer code

Data collection	*Provide a description of all commercial, open source and custom code used to collect the data in this study, specifying the version used OR state that no software was used.*
Data analysis	flow cytometry was analysed with Flowjo V10.6., histology and immuno staining with NanoZoomerslide NDP.view 2.5, Image J 153f51, single cell RNAseq and ATAC seq with CellRanger, CellRanger ATAC, R with AddModduleScore Ucell Packages, S16RNA sequencincing with r and Qiime2 2020.8. Statistics with prism V9.4. or Qiime2 2020.8

For manuscripts utilizing custom algorithms or software that are central to the research but not yet described in published literature, software must be made available to editors and reviewers. We strongly encourage code deposition in a community repository (e.g. GitHub). See the Nature Portfolio guidelines for submitting code & software for further information.

Data

Policy information about availability of data

All manuscripts must include a data availability statement. This statement should provide the following information, where applicable:

- Accession codes, unique identifiers, or web links for publicly available datasets
- A description of any restrictions on data availability
- For clinical datasets or third party data, please ensure that the statement adheres to our policy

ScRNAseq data accession number GSE 235513, bacteria sequencing

Research involving human participants, their data, or biological material

Policy information about studies with human participants or human data. See also policy information about sex, gender (identity/presentation), and sexual orientation and race, ethnicity and racism.

Reporting on sex and gender	We reanalyzed data from previous published studies mentioning these points ref 51-53.
Reporting on race, ethnicity, or other socially relevant groupings	We reanalyzed data from previous published studies mentioning these points ref 51-53.
Population characteristics	We reanalyzed data from previous published studies mentioning these points ref 51-53.
Recruitment	We reanalyzed data from previous published studies mentioning these points ref 51-53.
Ethics oversight	We reanalyzed data from previous published studies mentioning these points ref 51-53.

Note that full information on the approval of the study protocol must also be provided in the manuscript.

Field-specific reporting

Please select the one below that is the best fit for your research. If you are not sure, read the appropriate sections before making your selection.

☒ Life sciences ☐ Behavioural & social sciences ☐ Ecological, evolutionary & environmental sciences

For a reference copy of the document with all sections, see nature.com/documents/nr-reporting-summary-flat.pdf

Life sciences study design

All studies must disclose on these points even when the disclosure is negative.

Sample size	No statistical methods were used to predetermine sample size, but our sample sizes are similar to those reported in previous publications ref 54. The size of each groups was chosen according both statistical robustance for the type of experiment and ethic committee agreements. Data were routinely collected across independent replicates for each assay. Number of samples /mice and the number of independent experiments are indicated in the figure legends
Data exclusions	No data were excluded from the analysis
Replication	All experiments were repeated with complet independent set of mice. All attemps at replication were succesful. For scRNAseq and scATAC seq data were obtained from a pool of mice. For human data the numbers of patients are mentioned in figures
Randomization	No formal randomization was done as we compared mice with different genotypes
Blinding	Investigators were blinded to the mouse genotypes for all Facs staining process and acquisitiion as well as hsitology analysis. For scRNAseq and scATAC seq analysis as well microbiota composition the raw data were generated blind by the CRCL plateform, Active Motif , Belgium, and Genoscreen Lille, France, respectively. For the other experiments values were compared to standard our house keeping gene expression (ELISA, RT-PCR).

Reporting for specific materials, systems and methods

We require information from authors about some types of materials, experimental systems and methods used in many studies. Here, indicate whether each material, system or method listed is relevant to your study. If you are not sure if a list item applies to your research, read the appropriate section before selecting a response.

Materials & experimental systems

n/a	Involved in the study
☐	☒ Antibodies
☒	☐ Eukaryotic cell lines
☒	☐ Palaeontology and archaeology
☐	☒ Animals and other organisms
☒	☐ Clinical data
☒	☐ Dual use research of concern
☒	☐ Plants

Methods

n/a	Involved in the study
☒	☐ ChIP-seq
☐	☒ Flow cytometry
☒	☐ MRI-based neuroimaging

Antibodies

Antibodies used

CD45 APC-Cy7 (30-F11; BD biosciences), TCRb PE (H57-597; BD), CD3 BV650 (145-2C11, BD bioscience), CD4 BV711 (RM4-5; Biolegend), CD8 BV510 (53-6.7, BD Bioscience), CRTAM (11-5/CRTAM, Biolegend). IL-17A AlexaFluor700 (TC11-18H10; BD biosciences), IFN-g APC (XMG1.2; BD Biosciences), GM-CSF PerCP-Cy5.5 (MP1-22E9, Biolegend), TNFa PE-Cy7 (MP6-XT22, BD Bioscience), Granzyme B APC (GB11, Invitrogen). ROR-gt PE-CF594 (Q31-378; BD Bioscience), T-BET APC (eBio4B10, ebioscience), Granzyme B APC (GB11, Invitrogen). p-SMAD2/3 (D27F4, Cell Signaling) detected with a donkey anti-rabbit APC secondary antibody (A31573, Life Technology). For cell sorting, CD4 PE (GK1.5, eBioscience) TCRb APC (H57-597, BD Bioscience), CD45 APC-Cy7 (30-F11, BD biosciences). KLF6 PE (Santa cruz sc-365633). For immunofluorescence, γ-H2AX (Cell Signaling Cat #: 9718) Anti-Rabbit AlexaFluor 647 (A21245, Life Technologies), Anti-GFP (A-11122, Invitrogen), CD4 PE (GK1.5, eBioscience), anti-rabbit AlexaFluor488 (A32731, Invitrogen). All antibodies were used at the concentration of 1/200 except for ROR-gt PE-CF594 used at 1/400, and for p-SMAD2/3 and CRTAM, both used at 1/100. For IFNg depletion (XMG1.2, BioXCell) and IgG isotype control (HRPN-BioXCell)both at 200µg

Validation

Commercial Antibodies for flow cytometry and immuno staining were all validated by the fabricants. Except anti P SMAD2/3They were all directely conjugated. FMO was performed. Anti PSMAD2/3 was validated with or without TGF-b adding on the cells. For in vivo, treatement antibodies were valaidated by the fabricant, and IgG isotype control were used

Animals and other research organisms

Policy information about studies involving animals; ARRIVE guidelines recommended for reporting animal research, and Sex and Gender in Research

Laboratory animals

Mus musculus, on C57BL6 background, between 2-12 months of age. Except when mentionned, 6-8 month old animals were used. Both male and femal were used except for AOM DSS expertiments for which only were used

Wild animals

No wild animals were used in this study

Reporting on sex

No gender effects were reported and both sexs were used, except for AOM-DSS expriments for which it is estbalished that males are higher responder

Field-collected samples

No field collected animals were used in this study

Ethics oversight

Ethic committee agreement CECAPP#CLB 2017-017 APAFIS # 18685 from the French Ministery of research

Note that full information on the approval of the study protocol must also be provided in the manuscript.

Plants

Seed stocks

N/A

Novel plant genotypes

N/A

Authentication

N/A

Flow Cytometry

Plots

Confirm that:

☒ The axis labels state the marker and fluorochrome used (e.g. CD4-FITC).

☒ The axis scales are clearly visible. Include numbers along axes only for bottom left plot of group (a 'group' is an analysis of identical markers).

☒ All plots are contour plots with outliers or pseudocolor plots.

☒ A numerical value for number of cells or percentage (with statistics) is provided.

Methodology

Sample preparation	Sample preparation is described in the method section
Instrument	Data were acquired on BD Fortessa or AURORA Cytek machines Cells were purified on ARIA II (BD Biosciences)
Software	Data were analyzed using Flow jo v10.6.
Cell population abundance	In average 10 exp 5 YFP cells were analyzed .For very rare populations a minimum of 900 cells was analyzed
Gating strategy	Gating strategies are shown in extended data 2

☒ Tick this box to confirm that a figure exemplifying the gating strategy is provided in the Supplementary Information.
